# Synthesis and Antiproliferative Activity of Novel Heterocyclic Glycyrrhetinic Acid Derivatives

**DOI:** 10.3390/molecules24040766

**Published:** 2019-02-20

**Authors:** Daniela P. S. Alho, Jorge A. R. Salvador, Marta Cascante, Silvia Marin

**Affiliations:** 1 Laboratory of Pharmaceutical Chemistry, Faculty of Pharmacy, University of Coimbra, 3000-548 Coimbra, Portugal; dani.alho23@gmail.com; 2 Centre for Neuroscience and Cell Biology, 3004-504 Coimbra, Portugal; 3 Department of Biochemistry and Molecular Biomedicine, Faculty of Biology, University of Barcelona, Diagonal 643, 08028 Barcelona, Spain; martacascante@ub.edu; 4 Centro de Investigación Biomédica en Red de Enfermedades Hepáticas y Digestivas (CIBEREHD), Instituto de Salud Carlos III (ISCIII), 28029 Madrid, Spain

**Keywords:** pentacyclic triterpenoids, glycyrrhetinic acid, heterocyclic derivatives, antiproliferative activity, apoptosis

## Abstract

A new series of glycyrrhetinic acid derivatives has been synthesized via the introduction of different heterocyclic rings conjugated with an α,β-unsaturated ketone in its ring A. These new compounds were screened for their antiproliferative activity in a panel of nine human cancer cell lines. Compound **10** was the most active derivative, with an IC_50_ of 1.1 µM on Jurkat cells, which is 96-fold more potent than that of glycyrrhetinic acid, and was 4-fold more selective toward that cancer cell line. Further biological studies performed in Jurkat cells showed that compound **10** is a potent inducer of apoptosis that activates both the intrinsic and extrinsic pathways.

## 1. Introduction

Triterpenoids are a large group of natural compounds that are widely found in nature and are particularly prevalent in plants. They display a wide spectrum of biological and pharmacological activities. Within this group, the pentacyclic triterpenoids are the most potent compounds and their anti-inflammatory, antiviral, and anticancer effects have been extensively studied. Moreover, there is an increasing interest in these triterpenoids as lead compounds for the development of new anticancer agents, which is reflected in the large number of scientific papers and patents emerging in this field [1,2,3,4,5,6,7,8,9,10,11].

**Figure 1:**
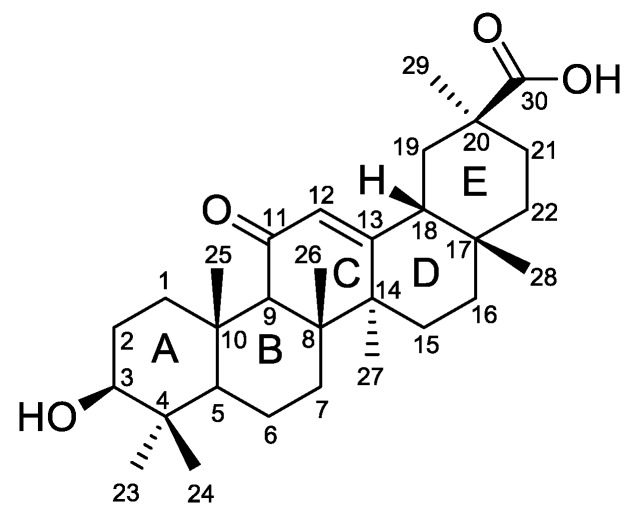
Chemical structure of glycyrrhetinic acid **1**.

Glycyrrhetinic acid (GA) **1** (Figure 1) is the aglycone of glycyrrhizin, a major pentacyclic triterpenoid saponin found in the roots of licorice (Glycyrrhiza species) [12]. This compound has a proven antiproliferative activity against several cancer cell lines and its anticancer effects were also observed in animal models [13,14,15,16]. Recent reports have shown that its cytotoxicity is mediated by the induction of apoptosis [16,17,18,19,20,21]. In addition, GA **1** can be extracted from the roots of licorice in high yields (up to 24%) [22]. These findings have increased its scientific interest as a scaffold for the development of new derivatives for potential cancer treatment [23,24,25,26,27].

In previous work performed by our group, introduction of imidazole, methyl-imidazole, and triazole heterocyclic rings was achieved in several positions of betulinic and ursolic acids, affording several derivatives with better antiproliferative activity compared with the parental compounds. The structure–activity relationship analysis revealed that the introduction of a heterocyclic ring in conjugation with an α,β-unsaturated ketone in ring A of the skeleton seems to provide the most potent derivatives [28,29].

Cycloaddition of azides and alkynes, commonly referred to as “click chemistry”, has acquired great interest in the development of novel heterocyclic compounds with varied biological activities [30,31,32,33]. This efficient and simple process has been successfully used in the synthesis of pentacyclic triterpenoid derivatives with improved anticancer activity [34,35,36,37].

The information provided above prompted us to synthesize new GA **1** derivatives via the introduction of different heterocyclic rings conjugated with an α,β-unsaturated ketone in its ring A. These novel semisynthetic derivatives were tested for their antiproliferative activity against a series of cancer cell lines. Further biological assays were performed for the most active compound **10** in the cancer cell line that yielded the best results (Jurkat cells), to elucidate its mechanism of action.

## 2. Results and Discussion

### 2.1. Chemistry

**Scheme 1:**
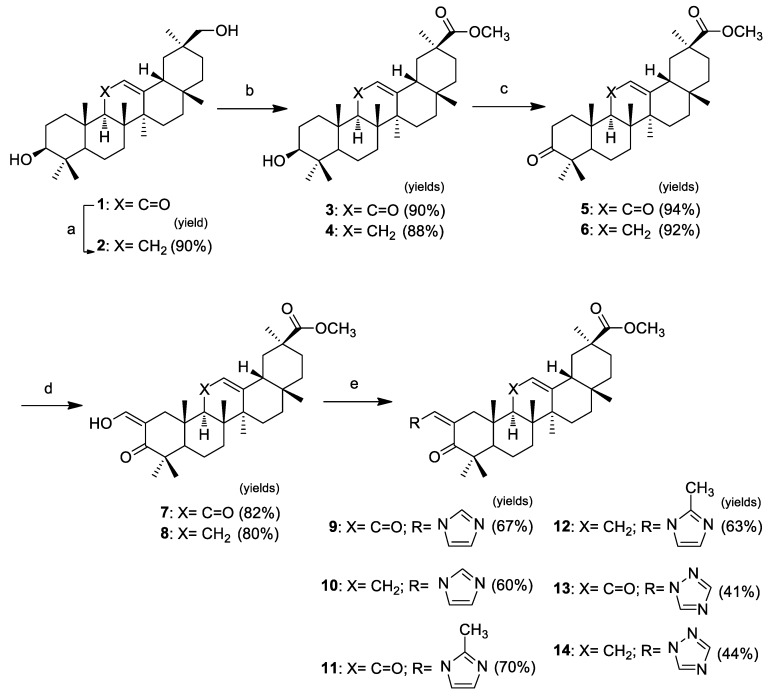
Reagents and conditions: (**a**) zinc dust, concentrated HCl, dioxane, r.t.; (**b**) CH_3_I, K_2_CO_3_, DMF, r.t.; (**c**) Jones reagent, acetone, 0 °C; (**d**) ethyl formate, NaOMe, benzene, r.t., N_2_; and (**e**) CDI, CBMI or CDT, dry THF, reflux, N_2_.

The synthesis of three pairs of novel GA 1 heterocyclic derivatives is summarized in Scheme 1. In addition to the intention to prepare heterocyclic derivatives with improved cytotoxicity, compounds **9**–**14** were synthesized to explore the effect of the keto group in position C-11 on the antiproliferative activity. Therefore, the first step performed in this sequence of reactions was the removal of the keto group via Clemmensen reduction with zinc dust and concentrated HCl in dioxane at room temperature, to afford **2** [38]. The following steps were executed in both original and reduced structures, providing successive pairs of derivatives. Methyl esters **3** and **4** were obtained from the reaction of compounds **1** and **2** with methyl iodide in the presence of potassium carbonate [39]. The 3β-hydroxyl group of these esters was oxidized using the Jones reagent [40], to give the 3-keto derivatives **5** and **6**. The reaction of these derivatives with ethyl formate and sodium methoxide allowed the preparation of the 2-hydroxyvinyl-3-keto compounds **7** and **8 [40]**. The heterocyclic derivatives **9**–**14** were prepared via the reaction of the vinyl alcohol at C-2 with the appropriate heterocyclic reagent, 1,1′-carbonyldiimidazole (CDI), 1,1′-carbonylbis(2′-methylimidazole) (CBMI), or 1,1′-carbonyl-di(1,2,4-triazole) (CDT) in reflux of THF under inert atmosphere [29], in yields ranging from 41% to 70%.

**Scheme 2:**
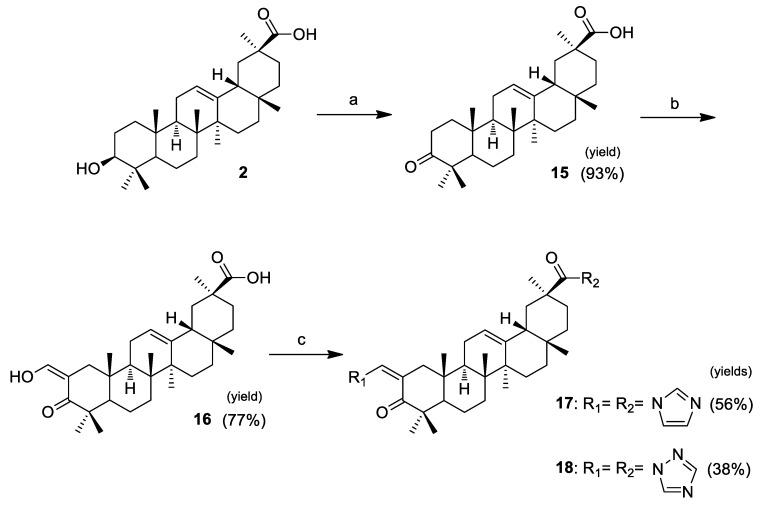
Reagents and conditions: (**a**) Jones reagent, acetone, 0 °C; (**b**) ethyl formate, NaOMe, benzene, r.t., N_2_; and (**c**) CDI or CDT, dry THF, reflux, N_2_.

Compounds **17** and **18** were synthesized as depicted in Scheme 2. These derivatives were prepared to evaluate if the presence of another heterocyclic ring at C-30 would benefit the antiproliferative activity. The steps of this sequence of reactions were performed using the same methods of oxidation, reaction with ethyl formate, and introduction of heterocyclic rings described in Scheme 1. These novel heterocyclic derivatives, **17** and **18**, were obtained in yields of 56% and 38%, respectively.

**Scheme 3:**
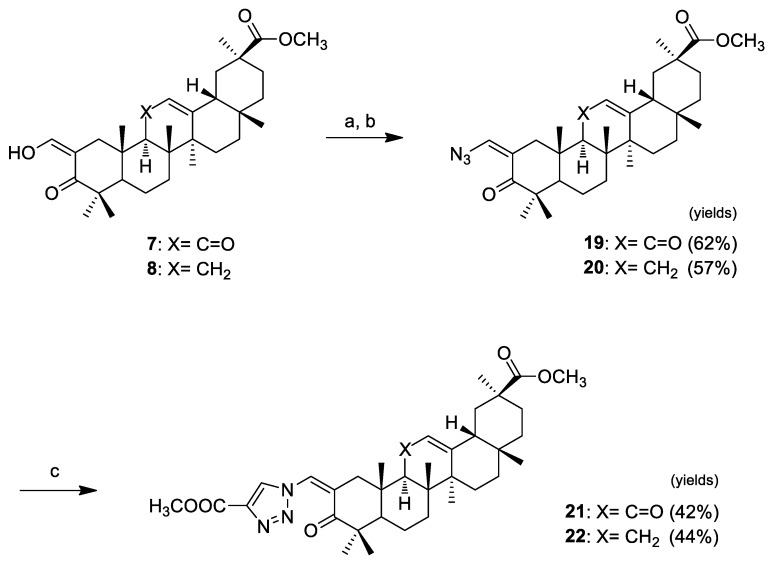
Reagents and conditions: (**a**) TsCl, Et_3_N, CH_2_Cl_2_, r.t.; (**b**) NaN_3_, acetone, r.t.; and (**c**) methyl propiolate, CuI, THF, 65 °C.

Click chemistry was handled as shown in Scheme 3. We decided to synthesize 1,4-substituted-triazolyl derivatives in conjugation with an α,β-unsaturated ketone in ring A of pentacyclic triterpenoids via Cu(I)-catalyzed azide-alkyne cycloaddition (CuAAc) with terminal alkynes. We prepared the azido derivatives **19** (62%) and **20** (57%) by tosylation of the vinyl alcohol at C-2 and subsequent replacement by azide. The triazolyl derivatives **21** and **22** were obtained via the “click reaction” of those compounds with methyl propiolate, catalyzed by CuI, in THF, at 65 °C (which were the best reaction conditions observed here), in yields of 42% and 44%, respectively.

Full structural elucidation of the new glycyrrhetinic acid derivatives was achieved using infrared spectroscopy (IR), nuclear magnetic resonance (NMR), and mass spectrometry (MS). The data obtained from these analytical techniques, for the known compounds 2–8, were in agreement with those reported in the literature [38,39,40].

The removal of the keto group at position C-11, via Clemmensen reduction, was confirmed by the absence of a band around 1664 cm^−1^ on the IR spectrum, which is present in glycyrrhetinic acid, and corresponds to the carbonyl group in ring C. On the ^13^C NMR spectrum, a δ signal around 200 ppm, which corresponds to the presence of that carbonyl group, was absent, and the δ signals for carbons C-12 and C-13 were present at higher field than that observed in compounds with the carbonyl group.

The preparation of compounds **7**, **8**, and **16** was confirmed by the presence of a δ signal at ~8.6 ppm in the ^1^H NMR spectra, corresponding to the proton in the exocyclic double bond at C-2. On the ^13^C NMR spectrum, the presence of this moiety was confirmed by the observation of a δ signal at ~188 ppm, corresponding to the exocyclic carbon, and a δ signal at ~106 ppm, corresponding to the C-2 carbon.

The successful preparation of the heterocyclic derivatives **9**–**14**, **17** and **18**, and **21** and **22**, was confirmed by the presence of a δ signal at ~7.7–8.0 ppm, corresponding to the proton of the exocyclic double bond at C-2 in the ^1^H NMR spectrum. On the ^13^C NMR spectrum the presence of this moiety was confirmed by the presence of a δ signal at ~127–131 ppm, corresponding to the exocyclic carbon.

The presence of heterocyclic ring(s) can be detected by the presence of extra δ signals in ^1^H and ^13^C NMR spectra. On the ^1^H NMR spectra, the imidazole group had three specific protons that appeared at values higher than 7 ppm, the methyl-imidazole group had two protons ranging from 6.9 to 7.7 ppm, the triazole group had two proton signals with a δ higher than 8 ppm, and the triazole group on compounds **21** and **22** had only one proton at ~8.3–8.4 ppm. In the ^13^C NMR spectra, the δ signals of the carbons of heterocyclic rings were present at values ranging from 118 to 153 ppm, varying in accordance with the different heterocyclic rings.

### 2.2. Biological Activity

#### 2.2.1. Antiproliferative Activity

Several cancer lines were cultured and used in experiments aimed at evaluating the potential cytotoxicity of the synthesized compounds against human cancers. This evaluation was based on the determination of IC_50_ values using 3-(4,5-dimethylthiozol-2-yl)-3,5-dipheryl tetrazolium bromide (MTT) or triphenyl tetrazolium chloride (XTT) assays after 72 h of treatment with the compounds.

All compounds were screened for their antiproliferative activity on the HT-29 (colon adenocarcinoma) cell line. As shown in Table 1, most of the novel heterocyclic derivatives (**9**–**14**, **17**, **18**, **21**, and **22**) showed improved cytotoxicity compared with the parental compound GA **1** and the intermediate compounds. The methylation of compounds **1** and **2** provided derivatives (**3** and **4**) that exhibited an important increment in antiproliferative activity, but lacked selectivity towards tumor cells [39]. The intermediates **7**, **8**, **16**, **19**, and **20**, which preceded the final heterocyclic derivatives, were also tested on the A549 (lung adenocarcinoma) cell line (Table 1). Analysis of the IC_50_ values of the heterocyclic derivatives with respect to their substrates in the two cancer cell lines showed that the introduction of a heterocyclic ring provided more potent compounds, with the exception of compound **13**.

The novel compounds **9**–**14**, **17**, **18**, **21**, and **22**, and the parental compound GA **1** were further tested in seven additional human cancer cell lines: MIAPaCa 2 (pancreas adenocarcinoma), HeLa (cervix adenocarcinoma), A375 (melanoma), MCF7 (breast adenocarcinoma), HepG2 (hepatocellular carcinoma), SH-SY5Y (neuroblastoma), and Jurkat (acute T cell leukemia) cells (Table 2). The synthesis of compounds **17** and **18** was performed to investigate the effect of the combined introduction of heterocyclic rings at the vinyl alcohol at the C-2 and at the C-30 positions. This combination resulted in a loss of cytotoxicity in all tested lines compared with compounds **10** and **14**, respectively. The preparation of the four pairs of derivatives **9**–**14**, **21**, and **22** allowed us to evaluate the effect of the keto group at position C-11 on the antiproliferative activity. The results of the assays in all tested cell lines were consistent and revealed that the compounds from which the keto group was removed from ring C were more potent. The type of heterocyclic ring conjugated with the α,β-unsaturated ketone in ring A also influenced the antiproliferative activity. Within the group of heterocyclic derivatives with a reduced ring C, compound **10,** which bears an imidazole ring, was the most active derivative in all tested cell lines. This compound was 31- to 96-fold more potent than GA **1**, depending on the cancer cell line. Moreover, with the exception of SH-SY5Y cells, compound **10** showed a similar or slightly improved antiproliferative activity against all cancer cell lines compared to the chemotherapy agent cisplatin [41,42,43,44,45,46] (Table 1 and Table 2).

The selectivity towards cancer cells was studied for GA **1** and compound **10** by incubating them with a human nontumoral cell line (BJ) (Table 2). Compound **10** and GA **1** showed IC_50_ values that were 6.3 and 1.6 times lower on Jurkat cells than on the nontumoral BJ cells, respectively. Therefore, the heterocyclic derivative **10** was more selective towards malignant cells than its parental compound **1**.

#### 2.2.2. Cell Viability over Time

**Figure 2:**
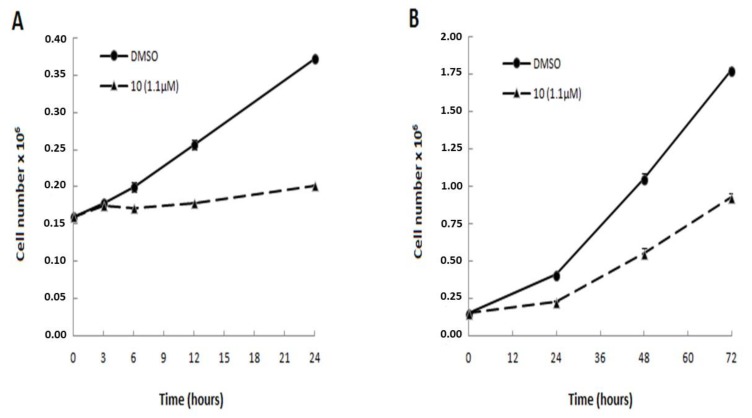
Effect of compound **10** on cell viability. Jurkat cells were treated with 1.1 μM compound **10** for different time periods ((**A**): 3, 6, 12, and 24 h; (**B**): 24, 48, and 72 h). Cell numbers were determined by Trypan Blue counting assays. Results are presented as means ± SD of three independent experiments.

The Jurkat cell line, which exhibited the best results regarding antiproliferative activity among the tested cancer cell lines, was used to study the mechanism of action of the most potent compound synthesized here, the heterocyclic derivative **10**. Trypan blue cell-counting assays were conducted to assess the antiproliferative profile of this compound over time. The counting of viable cells was performed after incubation of Jurkat cells with compound **10**, at a concentration corresponding to its IC_50_ value at 72 h of treatment, or with only the vehicle (control with DMSO). As shown in Figure 2, compound **10** affected the cell growth of Jurkat cells as early as at 6h of treatment. At this incubation time, a reduction of 18% of cell viability was observed in cells treated with compound **10** compared with cells that were not treated with the same compound. This effect on cell viability increased during next hours, until cell viability was 45% lower than it was in control cells at 24 h, and reached 50% at 72 h of treatment. Based on the antiproliferative profile obtained in these Trypan Blue counting experiments, we established the incubation times for the subsequent biological studies that were performed in this study.

#### 2.2.3. Analysis of Cell Cycle Distribution and Apoptosis

**Figure 3:**
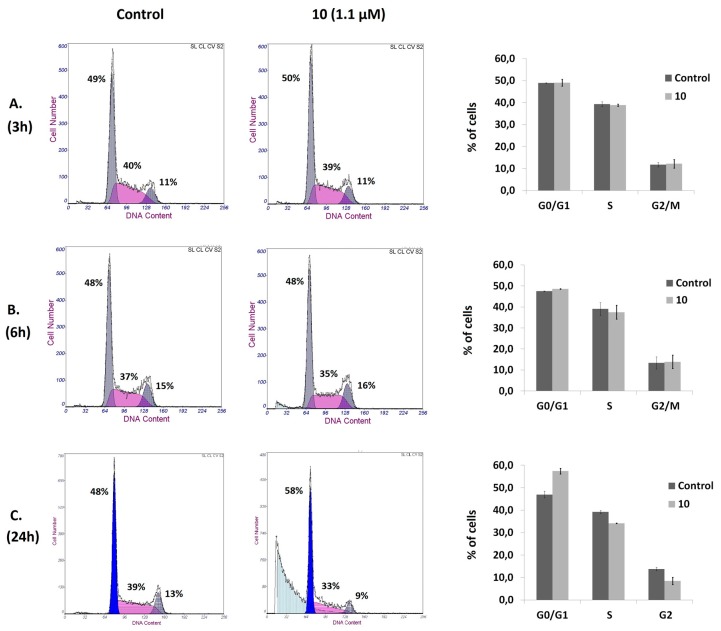
Effect of compound **10** on cell cycle distribution. Cell cycle analysis of Jurkat cells untreated (Control) or treated with 1.1 μM compound **10** for 3 (**A**), 6 (**B**), and 24h (**C**). After treatment, cells were stained with PI and DNA content analyzed by flow cytometry. A representative histogram is shown for each time of incubation and condition. Results are presented as means ± SD of three independent experiments.

Cell cycle and apoptosis assays were performed to elucidate the underlying antiproliferative mechanisms of compound **10**. To examine the effects on the cell cycle pattern, Jurkat cells were treated with compound **10,** at a concentration corresponding to its IC_50_ value at 72 h of treatment, for 3, 6, and 24 h. The study was performed using flow cytometry, and the calculation of the fraction of cells in phases G0/G1, S, and G2/M was executed using the fraction of live cells. No significant changes on the cell cycle distribution were detected after 3 and 6 h of exposure; treatment for 24 h increased the population at the G0/G1 phase, with a concomitant decrease in the populations of cells at the S and the G2/M phases with respect to the untreated cells (Figure 3). Hypodiploid sub-G0 peaks that correspond to cells with DNA fragmentation were observed after 6 and 24 h of incubation. These results suggest that compound **10** has the ability to induce cell death in Jurkat cells.

**Figure 4:**
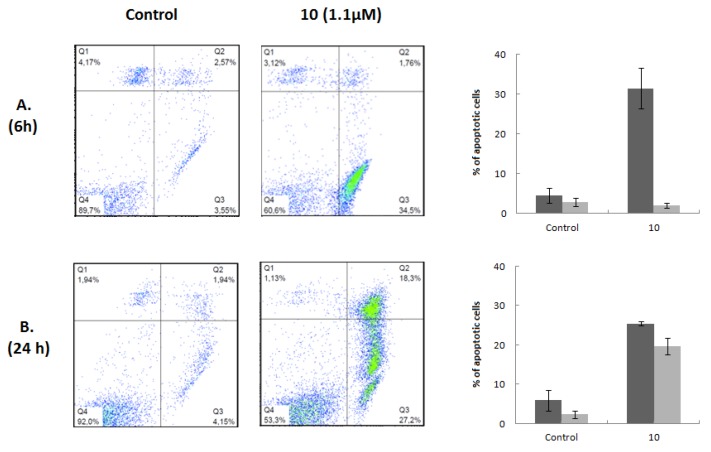
Induction of apoptosis by compound **10**. Flow cytometric quantification of apoptosis in Jurkat cells untreated (Control) or treated with 1.1 μM compound **10** for 6 (**A**) and 24 h (**B**). After treatment, cells were stained with annexin V-FICT/PI and analyzed by flow cytometry. A representative dot plot is shown for each time of incubation and condition; the right quadrants of each diagram (annexin^+^/PI^−^ and annexin^+^/PI^+^) represent apoptotic cells. The percentage of early (dark gray bar) and late (light gray bar) apoptotic cells in each condition is represented as a bars diagram, calculated from dot plots. Results are presented as means ± SD of three independent experiments.

Apoptosis assays were then conducted to elucidate the mechanism underlying the cytotoxic effect of this heterocyclic derivative. The Annexin V-FITC/PI flow cytometry assay employs the ability of Annexin V to bind to phosphatidylserine (PS) and the ability of propidium iodide (PI) to enter cells with damaged cell membranes and to bind to DNA. Early apoptosis is characterized by the loss of membrane asymmetry, with translocation of PS from the inner to the outer membrane, prior to the loss of membrane integrity. Therefore, this assay allows the discrimination of live cells (Annexin-V^−^/PI^−^) from early apoptotic (Annexin-V^+^/PI^−^), late apoptotic (Annexin-V^+^/PI^+^), or necrotic (Annexin-V^−^/PI^+^). Considering the results obtained in the cell viability and cell cycle experiments, we performed this assay on Jurkat cells after 6 and 24 h of treatment with compound **10** at a concentration corresponding to its IC_50_ value at 72 h of treatment. Flow cytometry dot plots representing Annexin V and PI staining are shown in Figure 4. Compound **10** induced 32% of cells to enter the early stage of apoptosis after 6 h of treatment. No significant changes were observed in the late apoptotic and necrotic populations. Exposure to the compound for 24 h increased the early apoptotic population by 25% and the late apoptotic population by 20%. The percentage of necrotic cells did not change significantly. We also performed this assay after 48 and 72 h of treatment; the Annexin V/PI profiles observed for these incubation times were similar to those obtained at 24 h (data not shown). The results of the Annexin V-FITC/PI assays are in good agreement with the hypodiploid sub-G0 peaks and their respective magnitudes, observed in the cell cycle experiments. Moreover, the fact that the appearance of G0/G1 arrest occurred after the beginning of the apoptotic events indicates that cell death was caused by a primary effect of compound **10** and not by the activation of apoptosis as a consequence of the inability of cells to overcome cell growth arrest and proceed through the cell cycle.

**Figure 5:**
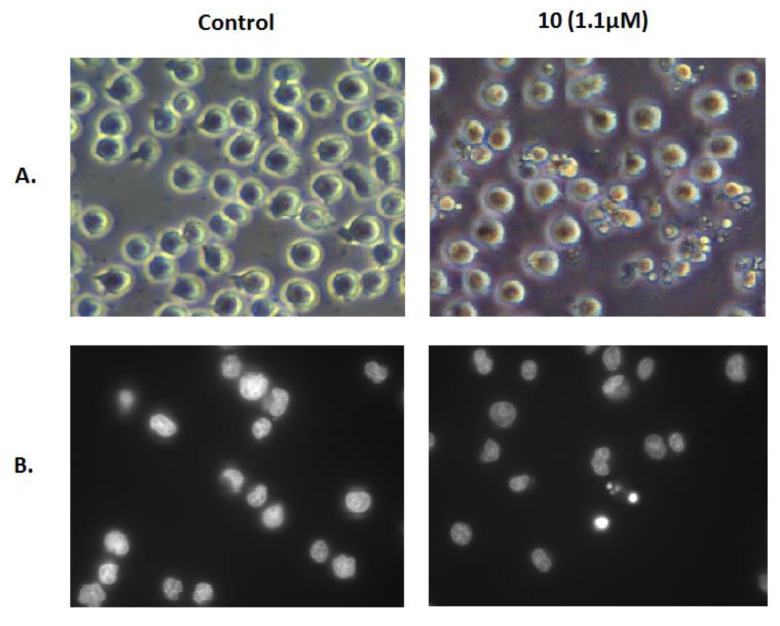
Morphological changes in Jurkat cells. Morphology of Jurkat cells untreated (Control) or treated with 1.1 μM compound **10** for 6 h was examined using a phase-contrast microscope (**A**) and fluorescence microscopy after Hoechst 33342 staining (**B**).

We further confirmed the induction of apoptosis by examining its characteristic morphological changes in Jurkat cells treated with the same concentration of compound **10** for 6 h. As shown in Figure 5, apoptotic bodies were observed in the treated cells using a phase-contrast microscope, and Hoechst 33342 staining showed that some of those cells exhibited a highly condensed nuclear morphology. In contrast, untreated cells presented intact plasma membranes and normal nuclear morphology.

Taken together, the results described above reveal that compound **10** is a potent inducer of apoptosis.

**Figure 6:**
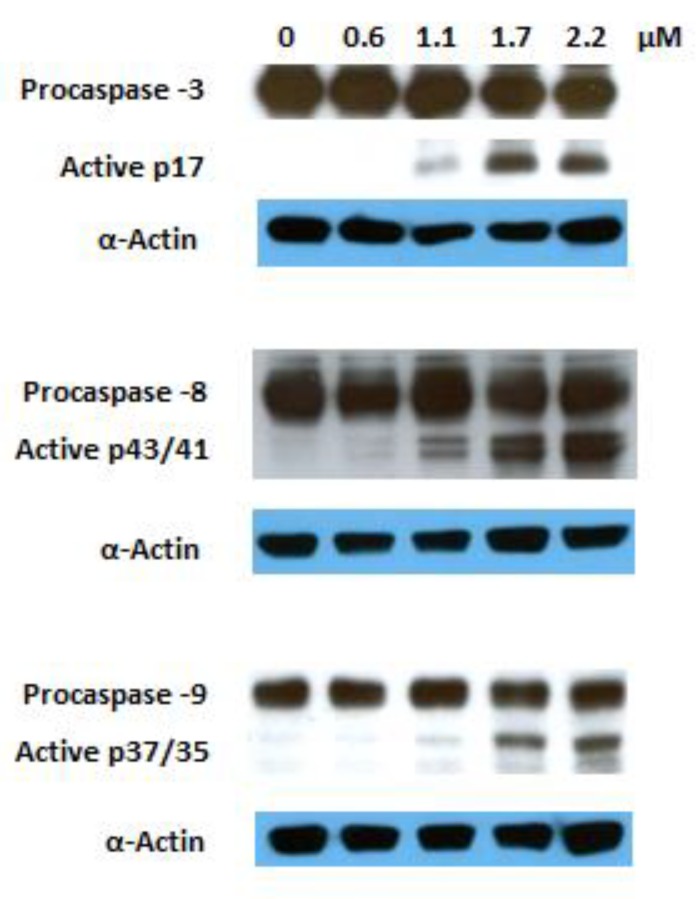
Effect of compound **10** on the activation of caspases 3, 8, and 9 in Jurkat cells after 6 h of treatment; α-actin was used as a loading control.

The activation of several caspases is involved in the execution phase of apoptosis. Caspase 8 is the upstream caspase for the extrinsic (death receptor) pathway, whereas caspase 9 is that of the intrinsic (mitochondrial) pathway. These pathways converge to caspase 3, the action of which is required for the morphological changes that are associated with apoptosis [47]. Previous reports have shown that GA **1** induces apoptosis involving both the extrinsic and intrinsic pathways [17,18,48,49,50,51]. To explore the mechanism via which the GA heterocyclic derivative **10** triggers this programmed cell death, we investigated its effects on the activation of caspases 3, 8, and 9 via Western blotting. The proteolytic cleavage of all mentioned caspases was observed after 6 h of treatment at a dose corresponding to the IC_50_ recorded at 72 h (Figure 6). These results indicate that the induced apoptosis is mediated by the activation of the both extrinsic and intrinsic pathways.

Apoptosis is a highly regulated process that occurs in physiological conditions and plays a major role in the pathogenesis of many diseases. In fact, acquired resistance towards apoptosis is a hallmark of most types of cancer. Despite its involvement in the carcinogenesis, apoptosis is a widespread target of many treatment strategies, including the development of novel chemotherapeutic molecules [47,52]. The p53 tumor suppressor protein is a critical component of the apoptotic signaling circuitry in both pathways. The loss of its function is the most common strategy employed by cancer cells to evade death. In fact, approximately 50% of human cancers bear *P53* gene mutations and, in the majority of the remaining cases, its function is compromised by different mechanisms [52,53,54]. The fact that Jurkat cells are a cancer p53-deficient cell line [55] indicates that compound **10** can activate the apoptotic machinery in a p53-independent manner.

Taken together, our results suggest that the induction of apoptosis involving both the intrinsic and extrinsic pathways is the main mechanism responsible for the antiproliferative activity of the GA heterocyclic derivative **10**. Efforts are currently underway to elucidate further its mechanism of action.

## 3. Materials and Methods

### 3.1. Chemistry

Glycyrrhetinic acid and all reagents were purchased from Sigma-Aldrich Co (St. Louis, MO, USA). The solvents used in the reactions were obtained from Merck Co (kenilworth, NJ, USA). and were purified and dried according to the literature procedures. The solvents used in workups were purchased from VWR Portugal (Radnor, PA, USA). Thin-layer chromatography (TLC) analysis was performed in Kieselgel 60HF254/Kieselgel 60G. Purification of compounds by flash column chromatography (FCC) was carried out using Kiesegel 60 (230–400 mesh, Merck) (kenilworth, NJ, USA). Melting points were determined using a BUCHI melting point B-540 apparatus and were uncorrected. IR spectra were obtained on a Fourier transform spectrometer. ^1^H and ^13^C NMR spectra (see Supplementary Materials) were recorded on a Bruker Avance-400 Digital NMR spectrometer, in CDCl_3_,_,_ with Me_4_Si as the internal standard. Chemical shifts values (δ) are given in parts per million (ppm) and coupling constants (*J*) are presented in hertz (Hz). Massa spectra were obtained using a Quadrupole/Ion Trap Mass Spectrometer (QIT-MS) (LCQ Advantage MAX, THERMO FINNINGAN). Elemental analysis was performed in an Analyzer Elemental Carlo Erba 1108 by chromatographic combustion.

*3β-hydroxy-olean-12-en-30-oic acid* (**2**): Compound **2** was prepared according to the literature [38], from **1** to give a white solid (90%). m.p.: 315–317 °C.

*Methyl 3β-hydroxy-11-oxo-olean-12-en-30-oate* (**3**): Compound **3** was prepared according to the literature [39], from **1** to give a colorless solid (90%). m.p.: 254–256 °C

*Methyl 3β-hydroxy-olean-12-en-30-oate* (**4**): Compound **4** was prepared from **2**, using the same method as for the preparation of **3**, with the obtention of a white solid (88%). m.p.: 239–242 °C.

*Methyl 3,11-dioxo-olean-12-en-30-oate* (**5**): Compound **5** was prepared according to the literature [40], from **3** to give a white solid (94%). m.p.: 248–250 °C.

*Methyl 3-oxo-olean-12-en-30-oate* (**6**): Compound **6** was prepared from **4**, using the same method as for the preparation of **5**, with the obtention of a white solid. (92%). m.p.: 185–188 °C

*Methyl 2-hydroxymethylene-3,11-dioxo-olean-12-en-30-oate* (**7**): Compound **7** was prepared according to the literature [40], from **5** to give a colorless solid (82%). m.p.: 231–234 °C.

*Methyl 2-hydroxymethylene-3-oxo-olean-12-en-30-oate* (**8**): Compound **8** was prepared from **6**, using the same method as for the preparation of **7**, with the obtention of a white solid (80%). m.p.: 136–139 °C.

*Methyl 2-(1H-imidazol-1-yl)-methylene-3,11-dioxo-olean-12-en-30-oate* (**9**): To a solution of compound **7** (300 mg, 0.59 mmol) in anhydrous THF (5 mL), CDI (191 mg, 1.18 mmol) was added. After 4 h under magnetic stirring at reflux temperature and N_2_ atmosphere, the reaction was completed. Water (50 mL) and ethyl acetate (50 mL) were added to the reaction mixture. The aqueous phase was further extracted with ethyl acetate (2 × 50 mL). The combined organic extract was then washed with water (2 × 50 mL) and brine (50 mL), dried over Na_2_SO_4_, filtered and evaporated to dryness. The resulting solid was subjected to FCC [petroleum ether/ ethyl acetate from (1:1) to (1:2)] to afford **9** as a white solid. (67%). m.p.: 249–251 °C. IR ν_max_/cm^−1^ (KBr): 3113, 2953, 1728, 1685, 1649, 1601, 1518, 1485, 1458, 1385, 1306, 1028. ^1^H NMR (400MHz, CDCl_3_): δ 7.76 (1H,s), 7.66 (1H, s), 7.33 (1H,s), 7.10 (1H, s), 5.75 (1H, s), 4.19 (1H, d, *J* = 16.5), 3.67 (3H, s), 2.52 (1H, s), 1.39 (3H, s), 1.18 (3H, s), 1.16 (3H, s), 1.13 (6H, s), 1.12 (3H, s), 0.81 (3H, s). ^13^C NMR (100MHz, CDCl_3_): δ 206.2, 199.2, 176.8, 170.6, 139.1, 130.7, 130.3, 128.4, 122.1, 119.0, 59.2, 52.8, 51.7, 48.4, 45.3, 44.8, 44.0, 43.3, 43.2, 41.2, 37.6, 35.9, 31.8, 31.3, 31.0, 29.7, 28.5, 28.2, 26.5, 26.3, 23.1, 22.3, 19.5, 17.9, 15.5. ESI-MS *m/z*: 561.71 ([M + H]^+^, 100%). Found C 74.45, H 8.80, N 5.05, calcd for C_35_H_48_N_2_O_4_^.^0: C 74.96, H 8.63, N 5.00%.

*Methyl 2-(1H-imidazol-1-yl)-methylene-3-oxo-olean-12-en-30-oate* (**10**): The method followed that described for compound **9** but using compound **8** (300 mg, 0.60 mmol) and CDI (195 mg, 1.20 mmol) in anhydrous THF (5 mL) at reflux for 5 h. The resulting solid was purified by FCC with petroleum ether/ethyl acetate (1:1) and afforded compound **10** as a white solid (60%). m.p.: 151–154 °C. IR ν_max_/cm^−1^ (KBr): 3118, 2951, 1730, 1687, 1610, 1518, 1487, 1458, 1383, 1306, 1028. ^1^H NMR (400MHz, CDCl_3_): δ 7.78 (1H,s), 7.70 (1H,s), 7.22 (1H,s), 7.15 (1H,s), 5.34 (1H, t, *J* = 3.4), 3.68 (3H, s), 2.90 (1H, d, *J* = 16.0), 1.18 (6H, s), 1.13 (6H, s), 1.02 (3H, s), 0.91 (3H, s), 0.78 (3H, s). ^13^C NMR (100MHz, CDCl_3_): δ 206.7, 177.6, 144.6, 138.7, 130.7, 130.5, 122.9, 122.0, 119.2, 52.6, 51.6, 48.4, 45.4, 45.2, 44.3, 43.2, 42.8, 41.9, 39.7, 38.3, 36.0, 32.0, 31.7, 31.3, 29.8, 28.5, 28.2, 27.0, 26.1, 25.7, 23.8, 22.5, 20.3, 16.4, 15.6. ESI-MS *m/z*: 547.73 ([M + H]^+^, 100%). Found C 76.72, H 9.43, N 4.98, calcd for C_35_H_50_N_2_O_3_: C 76.88, H 9.22, N 5.12%.

*Methyl 2-(2′-methyl-1H-imidazol-1-yl)-methylene-3,11-dioxo-olean-12-en-30-oate* (**11**): To a solution of compound **7** (300 mg, 0.59 mmol) in anhydrous THF (5 mL), CBMI (231 mg, 1.18 mmol) was added. After 2 h under magnetic stirring at reflux temperature and N_2_ atmosphere, the reaction was completed. The workup was performed according to the same method as for **9**. The resulting solid was submitted to FCC [petroleum ether/ ethyl acetate from (1:1) to (1:4)] to afford **11** as a white solid (70%). m.p.: 146–149 °C. IR ν_max_/cm^−1^ (KBr): 3116, 2953, 1730, 1686, 1657, 1608, 1541, 1502, 1458, 1412, 1385. ^1^H NMR (400MHz, CDCl_3_): δ 7.64 (1H,s), 7.29 (1H,s), 6.94 (1H,s), 5.75 (1H,s), 4.17 (1H, d, *J* = 16.3), 3.67 (3H, s), 2.52 (1H, s), 2.47 (3H, s), 1.39 (3H, s), 1.19 (3H, s), 1.17 (3H, s), 1.14 (9H, s), 0.82 (3H, s). ^13^C NMR (100MHz, CDCl_3_): δ 206.4, 199.3, 176.8, 170.5, 147.3, 129.9, 128.7, 128.5, 122.3, 118.1, 59.2, 53.0, 51.8, 48.5, 45.4, 44.9, 44.0, 43.4, 42.6, 41.2, 37.7, 36.0, 31.8, 31.3, 31.1, 29.7, 28.6, 28.3, 26.5, 26.3, 23.2, 22.4, 19.5, 18.0, 15.4, 13.7. ESI-MS *m/z*: 575.86 ([M + H]^+^, 100%). Found C 74.91, H 9.01, N 4.78, calcd for C_36_H_50_N_2_O_4_: C 75.22, H 8.77, N 4.87%.

*Methyl 2-(2′-methyl-1H-imidazol-1-yl)-methylene-3-oxo-olean-12-en-30-oate* (**12**): The method followed that described for compound **11** but using compound **8** (300 mg, 0.60 mmol) and CBMI (235 mg, 1.20 mmol) in anhydrous THF (5 mL) at reflux for 4 h. The resulting solid was subjected to FCC (petroleum ether/ ethyl acetate from (1:1) to (1:4)) to afford **12** as a white solid (63%). m.p.: 134–137 °C. IR ν_max_/cm^−1^ (KBr): 3118, 2951, 1730, 1687, 1606, 1541, 1502, 1456, 1412, 1383. ^1^H NMR (400MHz, CDCl_3_): δ 7.66 (1H,s), 7.14 (1H,s), 7.00 (1H,s), 5.33 (1H, t, *J* = 3.4), 3.67 (3H, s), 2.87 (1H, d, *J* = 15.9), 2.48 (3H, s), 1.18 (6H, s), 1.15 (3H, s), 1.12 (3H, s), 1.01 (3H, s), 0.90 (3H, s), 0.78 (3H, s). ^13^C NMR (100MHz, CDCl_3_): δ 206.7, 177.6, 147.3, 144.6, 130.2, 128.3, 123.8, 122.0, 118.2, 52.7, 51.6, 48.4, 45.4, 45.3, 44.3, 42.8, 42.5, 41.9, 39.7, 38.3, 36.1, 32.0, 31.7, 31.3, 29.7, 28.5, 28.2, 26.9, 26.1, 25.7, 23.7, 22.6, 20.3, 16.4, 15.5, 13.6. ESI-MS *m/z*: 561.77 ([M + H]^+^, 100%). Found C 76.85, H 9.67, N 4.64, calcd for C_36_H_52_N_2_O_3_: C 77.10, H 9.35, N 5.00%.

*Methyl 2-(1H-triazol-1-yl)-methylene-3,11-dioxo-olean-12-en-30-oate* (**13**): To a solution of compound **7** (300 mg, 0.59 mmol) in anhydrous THF (6 mL), CDT (581 mg, 3.54 mmol) was added. The workup was performed after 24 h of reaction, under magnetic stirring at reflux temperature and N_2_ atmosphere, using the same procedure as for compound **9**. The resulting solid was purified by FCC with petroleum ether/ ethyl acetate (1:2) and afforded **13** as a white solid (41%). m.p.: 148–151 °C. IR ν_max_/cm^−1^ (KBr): 3122, 2953, 1730, 1691, 1657, 1618, 1508, 1460, 1385. ^1^H NMR (400MHz, CDCl_3_): δ 8.40 (1H,s), 8.06 (1H,s), 7.80 (1H,s), 5.76 (1H,s), 4.42 (1H, d, *J* = 18.6), 3.69 (3H, s), 2.57, 1.40 (3H, s), 1.22 (3H, s), 1.17 (3H, s), 1.15 (3H, s), 1.14 (6H, s), 0.82 (3H, s). ^13^C NMR (100MHz, CDCl_3_): δ 206.4, 199.1, 176.9, 170.1, 152.7, 145.3, 128.7, 128.6, 125.1, 59.1, 53.0, 51.8, 48.5, 45.4, 44.9, 44.0, 43.7, 43.4, 41.2, 37.7, 35.7, 31.9, 31.4, 31.1, 29.8, 28.6, 28.3, 26.6, 26.4, 23.1, 22.4, 19.6, 18.0, 15.5. ESI-MS *m/z*: 274.48 (87%), 302.51 (15), 318.49 (100), 346.54 (22), 362.37 (37), 374.60 (11), 562.68 ([M + H]^+^, 93), 563.71 ([M + 2H] ^2+^, 26), 575.80 (24), 622.48 (15), 624.80 (21).

*Methyl 2-(1H-triazol-1-yl)-methylene-3-oxo-olean-12-en-30-oate* (**14**): The method followed that described for compound **13** but using compound **8** (300 mg, 0.60 mmol) and CDT (591 mg, 3.60 mmol) in anhydrous THF (6 mL) at reflux for 26 h. The resulting solid was purified by FCC with petroleum ether/ ethyl acetate (3:1) and afforded **14** as a white solid (44%). m.p.: 137–140 °C. IR ν_max_/cm^−1^ (KBr): 3122, 2951, 1732, 1689, 1622, 1512, 1456, 1383. ^1^H NMR (400MHz, CDCl_3_): δ 8.34 (1H,s), 8.07 (1H,s), 7.78 (1H,s), 5.36 (1H, t, *J* = 3.2), 3.69 (3H, s), 3.41 (1H, d, *J* = 17.6), 1.20 (3H, s), 1.19 (3H, s), 1.14 (3H, s), 1.13 (3H, s), 1.03 (3H, s), 0.92 (3H, s), 0.79 (3H, s). ^13^C NMR (100MHz, CDCl_3_): δ 207.4, 177.8, 153.1, 146.0, 144.5, 128.4, 125.6, 122.5, 52.9, 51.7, 48.6, 45.5, 45.3, 44.4, 43.5, 43.0, 42.0, 39.8, 38.5, 35.9, 32.2, 31.8, 31.4, 29.9, 28.7, 28.4, 27.1, 26.2, 25.8, 23.9, 22.6, 20.5, 16.5, 15.9. ESI-MS *m/z*: 274.53 (89%), 302.57 (23), 318.52 (100), 330.56 (14), 346.56 (27), 362.53 (25), 374.60 (16), 548.68 ([M + H]^+^, 93), 549.70 ([M + 2H] ^2+^, 28), 561.81 (23), 578.36 (20).

*3-Oxo-olean-12-en-30-oic acid* (**15**): Compound **15** was prepared from **2**, using the same method as for the preparation of **5**, with the obtention of a white solid (93%). m.p.: 245-248 °C. IR ν_max_/cm^−1^ (KBr): 3396, 2949, 1732, 1703, 1460, 1385. ^1^H NMR (400MHz, CDCl_3_): δ 5.31 (1H,t, *J* = 3.2), 2.33-2.59 (2H, m), 1.20 (3H, s), 1.14 (3H, s), 1.09 (3H, s), 1.06 (3H, s), 1.05 (3H, s), 1.01 (3H, s), 0.82 (3H, s). ^13^C NMR (100MHz, CDCl_3_): δ 217.9, 183.4, 144.2, 122.5, 55.3, 48.0, 47.4, 46.8, 44.0, 42.4, 41.6, 39.7, 39.2, 38.2, 36.6, 34.1, 32.1, 31.9, 31.0, 28.6, 28.1, 26.9, 26.4, 26.0, 25.8, 23.5, 21.4, 19.6, 16.6, 15.1. ESI-MS^2^ (25%) *m/z*: 274.98 ([M + H − C_12_H_20_O]^+^, 16%), 408.61 ([M + H − H_2_O − C_2_H_4_]^+^ or [M + H − HCOOH]^+^, 39); 437 ([M + H − H_2_O]^+^, 7), 455.42 ([M+H]^+^, 100).

*2-Hydroxymethylene-3-oxo-olean-12-en-30-oic acid* (**16**): The method followed that described for compound **7** but using compound **15** (850 mg, 1.87 mmol), ethyl formate (1.1 mL, 13.09 mmol) and sodium methoxide (1010 mg, 18.70 mmol), with the obtention of a white solid (77%). m.p.: 154–158 °C. IR ν_max_/cm^−1^ (KBr): 3442, 2953, 1731, 1699, 1643, 1587, 1456, 1383. ^1^H NMR (400MHz, CDCl_3_): δ 14.91 (1H, d, *J* = 3.0), 8.59 (1H, d, *J* = 2.7), 5.35 (1H, t, *J* = 3.4), 2.30 (1H, d, *J* = 14.4), 1.21 (3H, s), 1.20 (3H, s), 1.16 (3H, s), 1.13 (3H, s), 1.02 (3H, s), 0.93 (3H, s), 0.83 (3H, s). ^13^C NMR (100MHz, CDCl_3_): δ 190.8, 188.4, 183.4, 144.3, 122.6, 105.8, 52.1, 48.1, 45.6, 44.1, 42.6, 41.7, 40.2, 39.6, 39.4, 38.3, 36.2, 32.0, 31.9, 31.1, 28.7, 28.5, 28.2, 27.0, 26.1, 25.8, 23.5, 20.9, 19.6, 16.6, 14.7. ESI-MS^2^ (25%) *m/z*: 437.35 ([M + H − H_2_O − C_2_H_4_]^+^ or [M + H − HCOOH]+, 44%), 465.36 ([M + H − H_2_O]^+^, 100), 483.32 ([M + H]^+^, 56).

*30-(1H-Imidazol-1-yl)-3,30-dioxo-olean-12-en-2-(1H-imidazol-1-yl)-methylene* (**17**): Compound **17** was prepared using the same method as for the preparation of **9**, but using compound **16** (300 mg, 0.62 mmol) and CDI (302 mg, 1.86 mmol) in anhydrous THF (6 mL) at reflux for 6 h. The resulting solid was submitted to FCC (petroleum ether/ ethyl acetate from (1:2) to (1:8)) to afford **17** as a white solid (56%). m.p.: 162–165 °C. IR ν_max_/cm^−1^ (KBr): 3124, 2937, 1713, 1687, 1610, 1522, 1489, 1456, 1383, 1308, 1227, 1173, 1030. ^1^H NMR (400MHz, CDCl_3_): δ 8.30 (1H, s), 7.78 (1H, s), 7.70 (1H, s), 7.58 (1H, s), 7.24 (1H, s), 7.17 (1H, s), 7.08 (1H, s), 5.19 (1H, t, *J* = 3.4), 2.90 (1H, d, *J* = 16.0), 1.44 (3H, s), 1.19 (3H, s), 1.18 (3H, s), 1.13 (3H, s), 0.99 (3H, s), 0.90 (3H, s), 0.79 (3H, s). ^13^C NMR (100MHz, CDCl_3_): δ 206.6, 174.0, 143.8, 138.7, 137.2, 130.7, 130.6, 129.8, 122.8, 122.7, 119.2, 117.4, 52.5, 48.1, 46.6, 45.4, 45.2, 44.4, 43.2, 41.9, 39.6, 37.5, 35.9, 32.9, 32.2, 31.7, 29.8, 27.9, 27.9, 27.2, 25.9, 25.5, 23.7, 22.4, 20.3, 16.4, 15.6. ESI-MS^2^ (25%) *m/z*: 487.54 ([M + H − C_3_H_3_N_2_ − C_2_H_4_]^+^ or [M + H − C_4_H_3_N_2_O]^+^, 100%), 555.31 ([M + H − C_2_H_4_]^+^, 2), 583.44 ([M + H]^+^, 16).

*30-(1H-Triazol-1-yl)-3,30-dioxo-olean-12-en-2-(1H-triazol-1-yl)-methylene* (**18**): The method followed that described for compound **17** but using compound **16** (300 mg, 0.62 mmol) and CDT (1018 mg, 6.20 mmol) in anhydrous THF (6 mL) at reflux for 38 h. The resulting solid was submitted to FCC (petroleum ether/ ethyl acetate from (2:1) to (1:1)) to afford **18** as a white solid (38%). m.p.: 142–145 °C. IR ν_max_/cm^−1^ (KBr): 3126, 2954, 1713, 1695, 1618, 1512, 1456, 1383, 1279, 1161, 1132. ^1^H NMR (400MHz, CDCl_3_): δ 8.39 (1H, s), 8.25 (2H, s), 8.09 (1H, s), 7.80 (1H, s), 5.39 (1H, t, *J* = 3.4), 3.39 (1H, d, *J* = 17.5), 1.21 (3H, s), 1.20 (6H, s), 1.14 (3H, s), 1.03 (3H, s), 0.92 (3H, s), 0.82 (3H, s). ^13^C NMR (100MHz, CDCl_3_): δ 207.6, 182.0, 152.8, 146.9 (2), 146.0, 144.5, 128.4, 125.8, 122.6, 52.9, 48.4, 45.5, 45.3, 44.2, 43.5, 42.8, 42.0, 39.8, 38.4, 35.9, 32.2, 31.8, 31.3, 29.9, 28.9, 28.4, 27.1, 26.2, 25.8, 23.9, 22.6, 20.5, 16.5, 15.9. ESI-MS^2^ (35%) *m/z*: 488.42 ([M + H − C_2_H_3_N_3_ − C_2_H_4_]^+^ or [M + H − C_3_H_3_N_3_O]^+^, 100%), 514.64 ([M + H − C_4_H_6_O]^+^, 5), 584.67 (M^+^, 4), 585.77 ([M + H]^+^, 3).

*Methyl 2-azidomethylene-3,11-dioxo-olean-12-en-30-oate* (**19**): To a solution of compound **7** (700 mg, 1.37 mmol) in dichloromethane (14 mL), triethylamine (Et_3_N) (573 µL, 4.11 mmol) and tosyl chloride (TsCl) (654 mg, 3.43 mmol) were added. After 3.5 h, under magnetic stirring at room temperature, the reaction was completed. The solvent of the reaction mixture was removed under reduced pressure and the residue was suspended in acetone (14 mL); sodium azide (NaN_3_) (134 mg, 2.06 mmol) was added. After 5 h under magnetic stirring at room temperature, the reaction was completed. The acetone was removed under reduced pressure and water (60 mL) and ethyl acetate (60 mL) were added to the residue. The aqueous phase was further extracted with ethyl acetate (2 × 60 mL). The combined organic extract was then washed with water (3 × 50 mL), dried over Na_2_SO_4_, filtered and evaporated to the dryness, to afford a solid. The solid was subjected to flash FCC with petroleum ether/-ethyl acetate (15:1) to afford **19** as a white solid (62%). m.p.: 167–170 °C. IR ν_max_/cm^−1^ (KBr): 2951, 2127, 1730, 1659, 1460, 1387. ^1^H NMR (400MHz, CDCl_3_): δ 7.37 (1H, d, *J* = 1.5), 5.72 (1H, s), 3.76 (1H, d, *J* = 16.5), 3.69 (3H,s), 2.46 (1H,s), 1.37 (3H,s), 1.15 (3H,s), 1.14 (3H,s), 1.11 (3H,s), 1.09 (6H,s), 0.81 (3H,s). ^13^C NMR (100MHz, CDCl3): δ 205.0, 199.1, 176.9, 169.7, 137.2, 128.6, 123.0, 59.1, 53.4, 51.8, 48.3, 45.2, 44.9, 44.0, 43.3, 41.2, 40.6, 37.7, 35.7, 31.8, 31.6, 31.1, 29.3, 28.6, 28.3, 26.5, 26.4, 23.2, 22.5, 19.5, 18.0, 15.2. ESI-MS^2^ (35%) *m/z*: 462.38 ([M + H − C_3_H_6_O_2_]^+^, 100%), 508.46 ([M + H − CO]^+^ or [M + H − C_2_H_4_]^+^, 12), 518.37 ([M + H − H_2_O]^+^, 43), 536.61 ([M + H]^+^, 7).

*Methyl 2-azidomethylene-3-oxo-olean-12-en-30-oate* (**20**): Compound **20** was prepared using the same method as for the preparation of **19**, but using compound 8 (700 mg, 1.41 mmol) in dichloromethane (14 mL), Et_3_N (492 µL, 3.53 mmol), TsCl (538 mg, 2.82 mmol), and then NaN_3_ (138 mg, 2.12 mmol) in acetone (14 mL). The resulting solid was subjected to flash FCC with petroleum ether/ ethyl acetate (20:1) to afford **20** as a white solid (57%). m.p.: 186–189 °C. IR ν_max_/cm^−1^ (KBr): 2943, 2116, 1730, 1672, 1589, 1452, 1383. ^1^H NMR (400MHz, CDCl_3_): δ 7.36 (1H, d, *J* = 1.6), 5.33 (1H, t, *J* = 3.4), 3.68 (3H,s), 2.66 (1H, d, *J* = 16.6), 1.15 (3H,s), 1.13 (3H,s), 1.09 (6H,s), 1.01 (3H,s), 0.88 (3H,s), 0.79 (3H,s). ^13^C NMR (100MHz, CDCl_3_): δ 205.7, 177.6, 144.3, 137.1, 123.2, 122.4, 53.1, 51.6, 48.3, 45.2, 45.1, 44.3, 42.8, 41.8, 40.2, 39.6, 38.3, 35.7, 32.0, 31.8, 31.3, 29.2, 28.5, 28.2, 27.0, 26.0, 25.7, 23.6, 22.6, 20.3, 16.4, 15.4. ESI-MS^2^ (35%) *m/z*: 462.48 ([M + H − H_3_COH − C_2_H_4_]^+^ or [M + H − H_3_CCOOH]^+^, 32%), 504.31 ([M + H − H_2_O]^+^, 100), 522.38 ([M + H]^+^, 7).

Compound **21**: To a solution of compound **19** (350 mg, 0.65 mmol) in THF (7 mL), methyl propiolate (64.4 µL, 0.72 mmol), and CuI (12.4 mg, 0.065 mmol) were added. After 4 h under magnetic stirring at 65 °C, the reaction was completed. The reaction mixture was filtered and evaporated to dryness. The resulting solid was purified by FCC (petroleum ether/ ethyl acetate from (8:1) to (2:1)) to afford **21** as a white solid (42%). m.p.: 184–186 °C. IR ν_max_/cm^−1^ (KBr): 3136, 2951, 1747, 1726, 1691, 1651, 1620, 1554, 1458, 1385, 1209, 1034. ^1^H NMR (400MHz, CDCl_3_): δ 8.42 (1H, s), 7.99 (1H, s), 5.74 (1H,s), 4.17 (1H, d, *J* = 17.5), 3.96 (3H, s), 3.69 (3H,s), 2.53 (1H,s), 1.41 (3H, s), 1.22 (3H, s), 1.16 (3H,s), 1.14 (9H,s), 0.81 (3H,s). ^13^C NMR (100MHz, CDCl_3_): δ 205.8, 198.8, 176.9, 170.3, 160.7, 140.1, 128.8, 128.5, 128.2, 127.7, 59.0, 53.0, 52.4, 51.8, 48.5, 45.6, 44.9, 44.0, 43.6, 43.4, 41.2, 37.7, 35.9, 31.9, 31.3, 31.1, 29.7, 28.6, 28.3, 26.5, 26.4, 23.1, 22.3, 19.5, 17.9, 15.6. ESI-MS *m/z*: 620.59 ([M + H]^+^, 100%). Found C 69.39, H 8.21, N 6.66, calcd for C_36_H_49_N_3_O_6_: C 69.76, H 7.97, N 6.78 %.

Compound **22**: Compound **22** was prepared using the same method as for the preparation of **21**, but using compound **20** (350 mg, 0.67 mmol), methyl propiolate (66.0 µL, 0.74 mmol), and CuI (12.8 mg, 0.067 mmol). The resulting solid was purified by FCC [petroleum ether / ethyl acetate from (8:1) to (2:1)] to afford **22** as a white solid (44%). m.p.: 125–127 °C. IR ν_max_/cm^−1^ (KBr): 3143, 2953, 1730, 1699, 1620, 1556, 1456, 1385, 1221, 1034. ^1^H NMR (400MHz, CDCl_3_): δ 8.30 (1H, s), 7.90 (1H, s), 5.32 (1H, t, *J* = 3.4), 3.98 (3H, s), 3.68 (3H,s), 3.18 (1H, d, *J* = 18.5), 1.21 (3H, s), 1.18 (3H,s), 1.15 (3H, s), 1.13 (3H, s), 1.01 (3H,s), 0.92 (3H,s), 0.78 (3H, s). ^13^C NMR (100MHz, CDCl_3_): δ 206.6, 177.6, 160.7, 144.3, 139.8, 129.2, 128.5, 128.3, 122.2, 52.7, 52.5, 51.6, 48.4, 45.5, 45.2, 44.2, 43.6, 42.7, 41.8, 39.6, 38.3, 35.9, 32.0, 31.6, 31.3, 29.6, 28.5, 28.2, 26.9, 26.0, 25.6, 23.7, 22.4, 20.3, 16.3, 15.8. ESI-MS *m/z*: 606.33 ([M + H]^+^, 100%) Found C 71.58, H 8.58, N 6.88, calcd for C_36_H_51_N_3_O_5_: C 71.37, H 8.49, N 6.94 %.

### 3.2. Biology

HT-29, A549, MIA Paca 2, HeLa, A375, MCF7, HepG2, SH-SY5Y, Jurkat, and BJ cells were purchased from the American Type Culture Collection (ATCC, Rockville, MD). Dulbecco’s Modified Eagle’s Medium (DMEM), Roswell Park Memorial Institute (RPMI)-1640 medium, Phosphate Buffered Saline (PBS), glucose 45%, human insulin 10 mg/mL, dimethyl sulfoxide (DMSO), 3-(4,5-dimethylthiazol-2-yl)-2,5-diphenyl tetrazolium bromide (MTT) powder, Trypan Blue (TB) 0.4%, propidium iodide (PI), Hoescht 33342, and protease inhibitor cocktail were obtained from Sigma-Aldrich Co. (St. Luis, MO, USA). Minimum Essential Medium (MEM), penicillin/streptomycin (P/S) and L-glutamine were purchased from Gibco-BRL (Eggenstein, Germany). Sodium pyruvate, trypsin/EDTA (0.05%/0.02%), MEM-Eagle nonessential amino acids 100x and ECL Western Blotting Detection Kit Reagent were obtained from Biological Industries (Kibbutz Beit Haemek, Israel). Fetal Bovine Serum (FBS) was obtained from PAA Laboratories (Pasching, Austria), the Cell Proliferation Kit II (XTT kit) was purchased from Roche (Roche Molecular Biochemicals, Indianapolis, IN, USA), and annexin V-FITC was obtained from Bender MedSystems (Vienna, Austria). Primary antibodies against caspases 3 (#9662), 8 (#9746) and 9 (#9502) were purchased from Cell Signaling Technology (Beverly, MA, USA) and primary antibody against α-actin (#69100) was obtained from MP Biomedicals (Santa Ana, CA, USA). Secondary antibodies HRP-conjugated goat anti-rabbit (NA934) and HRP-conjugated rabbit anti-mouse (P02060) were purchased from Amersham Biosciences (Uppsala, Sweden) and DAKO (Copenhagen, Denmark), respectively.

Stock solutions of 20 mM in DMSO of the synthesized compounds were prepared and stored at −20 °C. Working solutions were prepared in culture medium and appropriate amounts of DMSO were included in controls; all solutions had a final concentration of 0.5% DMSO.

#### 3.2.1. Cell Culture

HT-29, A549, MIA Paca 2, HeLa, A375, and SH-SY5Y cells were cultured in DMEM supplemented with 10% heat-inactivated FBS and 1% P/S. HepG2 and BJ cells were grown in DMEM supplemented with 10% heat-inactivated FBS, 1% P/S and 1 mM sodium pyruvate. Jurkat cells were cultured in RPMI-1640 medium supplemented with 10% heat-inactivated FBS, 1% P/S, and 2 mM L-glutamine. MCF7 cells were maintained in MEM supplemented with 10% heat-inactivated FBS, 0.1% P/S, 1 mM sodium pyruvate, 2 mM L-glutamine, 1x MEM-Eagle nonessential amino acids, 0.01mg/mL insulin human and 10 mM glucose.

All cell cultures were incubated in a 5% CO_2_ humidified atmosphere at 37 °C.

#### 3.2.2. Antiproliferative Activity Assays

The antiproliferative activity of the synthesized compounds on HT-29, A549, MIA Paca 2, HeLa, A375, MCF7, HepG2, and BJ cells was determined by the MTT assay. Exponentially growing cells were plated in 96-well plates at a density of 1–8 × 10^3^ cells/ well. After 24 h of incubation, the growth medium was replaced with fresh medium containing either the compounds dissolved in DMSO at different concentrations or only with DMSO, in triplicate, and the cells were continued to culture for 72 h. After incubation with the compounds, the medium was removed and 100 µL of MTT solution (0.5 mg/mL) were added to each well and the plates were incubated for 1 h. MTT was removed and 100 µL of DMSO was added to dissolve the formazan crystals. The absorbance was immediately read at 550 nm on an ELISA plate reader (Tecan Sunrise MR20-301, TECAN, Austria). For SH-SY5Y and Jurkat cells, the antiproliferative activity was determined by XTT assay. SH-SY5Y cells were plated with 1.6 × 10^4^ cells/well in 96-well plates in 100 µL medium. The compounds at different concentrations or only the vehicle (medium with 0.5% DMSO), in 100 μL, were added 24 h after seeding, in triplicate, and incubated for 72 h. For Jurkat cells, 5.5 × 10^3^ cells/well were plated simultaneously with the addition of the different concentrations of compounds or vehicle, in triplicate, and were allowed to incubate for 72 h. In both cases, after that incubation period, 100 μL of the XTT labeling mixture was added to each well and the plates were incubated again for 4 h. Then, the absorbance was read at 450 nm on the ELISA plate reader.

Concentrations that inhibit cell proliferation by 50% (IC_50_) represent an average of a minimum of three independent experiments and were expressed as means ± standard deviation (SD).

#### 3.2.3. Cell Viability over Time Assays

The viability over time of Jurkat cells, treated with compound **10**, was assessed by TB cell counting experiments. For these assays, 1.6 × 10^5^ cells were plated per well in 6-well plates simultaneously with the addition of compound **10**, in the concentration of its IC_50_ value at 72 h, or only vehicle, in a total volume of 2 mL of medium. After each incubation time (3, 6, 12, 24, 48, and 72h), the suspension of cells of each well was collected. Equal amounts of TB solution and suspension were mixed and the mixture was placed in a Neubauer chamber in order to perform the counting. The number of cells determined for each condition, in each incubation time, represents an average of three independent experiments, with two replicates per experiment.

#### 3.2.4. Cell Cycle Analysis

Cell cycle was assessed by flow cytometry using a fluorescence-activated cell sorter (FACS). Jurkat cells were plated in 6-well plates at a density of 1.6 × 10^5^ cells, simultaneously with the addition of compound **10**, at a concentration corresponding to its IC_50_ value at 72 h of treatment, or with only the vehicle, in a total volume of 2 mL of medium. The cells were allowed to incubate for 3, 6, and 24 h. After incubation, cells were collected and centrifuged. The supernatant was removed and the pellet was resuspended in 1 mL of TBS containing 1mg/mL PI, 10mg/mL RNase free of DNase, and 0.1% Igepal CA-630, for 1 h, at 4 °C. FACS analysis was performed at 488 nm in an Epics XL flow cytometer (Coulter Corporation, Hialeah, FL, USA). Data were collected and analyzed using the Multicycle software (Phoenix Flow Systems, San Diego, CA, USA). Three independent experiments were performed, with two replicates per experiment.

#### 3.2.5. Annexin V-FITC/PI Flow Cytometry Assay

Apoptosis was assessed by flow cytometry using a FACS. The same number of Jurkat cells taken for the cell cycle assay was treated as described above. The cells were allowed to incubate for 6 and 24 h. After incubation, cells were collected and centrifuged. The supernatant was removed and the pellet was resuspended in 95 µL of binding buffer (10 mM HEPES/NaOH, pH 7.4, 140 mM NaCl, 2.5 mM CaCl_2_). Annexin V-FITC conjugate (3 µL) was added and cells were incubated for 30 min, at room temperature, in darkness. After incubation, 0.8 mL of binding buffer was added. Just before the FACS analysis, cells were stained with 20 µL of 1mg/mL PI solution.

Three independent experiments were performed, with two replicates per experiment.

#### 3.2.6. Observation of Morphological Changes

The morphological changes were observed by fluorescence microscopy using Hoechst staining. Jurkat cells were plated in 6-well plates at a density of 1.6 × 10^5^ cells, simultaneously with the addition of compound **10**, at a concentration corresponding to its IC_50_ value at 72 h of treatment, or with only the vehicle, in a total volume of 2 mL of medium (six wells per condition). The cells were incubated for 6 h. Before collecting, morphological changes were observed under a phase-contrast microscope. Cells were collected by centrifugation, washed twice with PBS and stained with 500 µL of Hoechst 33342 solution (2 µg/mL in PBS) for 15 min, at room temperature, in darkness. Finally, cells were washed and resuspended in 10 µL PBS. The samples were mounted on a slide and observed with a fluorescence microscope (DMRB, Leica Microssystems, Wetzlar, Germany) with a DAPI filter. Three independent experiments were conducted.

#### 3.2.7. Western Blot Analysis

Jurkat cells (1.6 × 10^6^ cells) were cultured in 25 cm^2^ flasks and treated for 6 h with compound **10** at different concentrations. After incubation, the cells were washed twice with ice-cold PBS and resuspended in lysis buffer containing 20 mM Tris/ acetate, pH 7.5, 270 mM sucrose, 1 mM EDTA, 1 mM EGTA, 1% Triton X-100, and 1% protease inhibitor cocktail. The samples were sonicated, incubated on ice for 20 min, and centrifuged at 13,200 rpm for 12 min at 4 °C, and the pellets were discarded. The supernatants were assayed for protein concentration using a BCA kit (Thermo fisher Scientific, Waltham, MA, USA). Protein extracts (50 μg) were separated on 15% sodium dodecyl sulfate (SDS)-polyacrylamide gels and transferred to a polyvinylnitrocellulose transfer membrane (Bio-Rad Laboratories, Hercules, CA, USA). The membranes were blocked by incubation in TBS buffer (20 mM Tris, pH = 7.5 and 132 mM NaCl) containing 0.1% of Tween and 5% of BSA or nonfat milk, for 1 h, at room temperature. Then, membranes were blotted with the primary antibodies anti-caspase 3, 8 and 9 (1:1000) overnight at 4 °C. The blots were washed three times with TBS-0.1% Tween and incubated with the appropriate secondary antibodies, for 1 h, at room temperature. Before protein detection, membranes were washed again five times with TBS-0.1% Tween. Immunocomplexes were visualized using the Immobilon ECL Western Blotting Detection Kit Reagent. Three independent experiments were performed.

## 4. Conclusions

In this study, we synthesized a series of novel GA derivatives via the introduction of different heterocyclic rings conjugated with an α,β-unsaturated ketone in its ring A. Screening for antiproliferative activity in a panel of nine human cancer cell lines showed that the most active compound **10** was 31- to 96-fold more potent than GA. This derivative was also more selective towards tumor cells. Further biological studies performed in Jurkat cells suggest that potent apoptosis induction involving both the intrinsic and extrinsic pathways is the main mechanism underlying the antiproliferative activity of compound **10**. The enhanced potency and selectivity, and the biological activity of this new GA heterocyclic derivative warrant further preclinical evaluation.

## Figures and Tables

**Table 1:** Antiproliferative activities of GA, its derivatives, and cisplatin against HT-29 and A549 cell lines.

Compound	Cell line (IC_50_, µM) ^1^	
	HT-29	A549
**1**	115.7 ± 1.6	110.5 ± 3.9
**2**	88.1 ± 1.4	N.D.
**3**	19.6 ± 0.6	N.D.
**4**	18.5 ± 0.9	N.D.
**5**	46.3 ± 2.3	N.D.
**6**	63.9 ± 1.1	N.D.
**7**	14.3 ± 0.3	18.5 ± 1.5
**8**	14.0 ± 0.2	17.0 ± 0.6
**9**	11.5 ± 0.5	11.1 ± 0.1
**10**	3.3 ± 0.2	2.8 ± 0.2
**11**	9.4 ± 0.7	10.3 ± 0.6
**12**	3.6 ± 0.1	3.1 ± 0.1
**13**	31.2 ± 1.5	24.7 ± 0.9
**14**	12.1 ± 0.2	12.3 ± 0.6
**15**	92.3 ± 1.5	N.D.
**16**	60.3 ± 2.0	54.4 ± 2.6
**17**	22.4 ± 0.5	23.1 ± 0.8
**18**	21.8 ± 1.6	24.5 ± 0.6
**19**	38.5 ± 0.6	48.5 ± 0.8
**20**	36.3 ± 1.4	44.3 ± 3.6
**21**	11.0 ± 0.8	10.6 ± 0.1
**22**	8.9 ± 0.5	7.9 ± 0.4
**Cisplatin**	6.1 [41]	12.6 ± 0.8 [42]

^1^ The cell lines were treated with different concentrations of each compound for 72 h. IC_50_ values were determined by 3-(4,5-dimethylthiozol-2-yl)-3,5-dipheryl tetrazolium bromide (MTT) assay and are expressed as means ± SD (standard deviation) of three independent experiments. IC_50_ is the concentration that inhibits 50% of cellular growth. N.D.: not determined.

**Table 2:** Antiproliferative activities of GA, its heterocyclic derivatives, and cisplatin against several cancer cell lines and the nontumoral BJ cell line.

Compound	Cell line (IC_50_, µM) ^1^							
	MIAPaca2	HeLa	A375	MCF7	HepG2	SH-SY5Y	Jurkat	BJ
**1**	101.6 ± 1.6	107.2 ± 2.5	112.2 ± 2.6	97.8 ± 3.9	125.1 ± 9.1	109.7 ± 2.5	105.8 ± 5.0	165.0 ± 7.1
**9**	14.2 ± 0.9	12.4 ± 1.2	10.4 ± 1.0	6.4 ± 0.3	14.3 ± 0.3	6.0 ± 0.2	3.2 ± 0.1	N.D.
**10**	3.3 ± 0.2	2.2 ± 0.1	2.0 ± 0.2	3.0 ± 0.2	3.1 ± 0.1	1.7 ± 0.15	1.1 ± 0.06	6.9 ± 0.07
**11**	10.8 ± 0.2	10.9 ± 1.0	7.1 ± 0.3	5.6 ± 0.3	13.5 ± 0.4	5.6 ± 0.2	2.4 ± 0.1	N.D.
**12**	3.3 ± 0.2	2.6 ± 0.2	2.3 ± 0.2	3.2 ± 0.3	3.5 ± 0.2	2.2 ± 0.2	1.3 ± 0.12	N.D.
**13**	28.7 ± 2.6	28.9 ± 1.7	26.5 ± 0.9	N.D.	N.D.	N.D.	N.D.	N.D.
**14**	13.4 ± 0.5	11.8 ± 0.7	10.3 ± 1.0	N.D.	N.D.	N.D.	N.D.	N.D.
**17**	17.8 ± 1.1	22.2 ± 0.8	17.9 ± 0.2	N.D.	N.D.	N.D.	N.D.	N.D.
**18**	15.2 ± 0.5	17.9 ± 0.6	13.4 ± 1.0	N.D.	N.D.	N.D.	N.D.	N.D.
**21**	12.0 ± 1.0	10.6 ± 0.1	7.2 ± 0.5	6.0 ± 0.3	11.8 ± 0.4	3.7 ± 0.1	1.7 ± 0.10	N.D.
**22**	6.9 ± 0.3	5.4 ± 0.3	4.9 ± 0.1	5.2 ± 0.2	9.0 ± 0.1	3.2 ± 0.1	1.5 ± 0.12	N.D.
**Cisplatin**	5.0 ± 1.0 [46]	2.3 ± 0.3 [43]	3.1 ± 1.0 [42]	19.1 ± 4.5 [43]	2.9 [41]	0.7 ± 0.1 [44]	1.9 [45]	10.1 ± 2.0 [43]

^1^ The cell lines were treated with different concentrations of each compound for 72 h. IC_50_ values were determined by XTT assay in Jurkat and SH-SY5Y cells and by MTT assay in all the other cell lines. Results are expressed as means ± SD of three independent experiments. IC_50_ is the concentration that inhibits 50% of cellular growth. N.D.: not determined.

## Supplementary Materials

^1^H-NMR and ^13^C-NMR spectra of selected compounds are available online.
