# Supplementary material for: Synthesis and Antiproliferative Activity of Novel Heterocyclic Glycyrrhetinic Acid Derivatives
Source: Molecules. 2019 Feb 20;24(4):766. doi: 10.3390/molecules24040766 (PMC6412232; doi:10.3390/molecules24040766)

Supplementary information

Synthesis and anticancer activity of novel heterocyclic glycyrrhetinic acid derivatives

Daniela P. S. Alho [1,2], Jorge A. R. Salvador [1,2,*], Marta Cascante [3] and Silvia Marín [3,*]

[1] Laboratory of Pharmaceutical Chemistry, Faculty of Pharmacy, University of Coimbra, 3000-548 Coimbra, Portugal.

[2] Center of Neuroscience and Cell Biology, Coimbra, Portugal.

[3] Department of Biochemistry and Molecular Biomedicine, Faculty of Biology, University of Barcelona, Diagonal 643, 08028 Barcelona.

Table of contents

1. NMR data for compound 8 4
    1.1. ^{1}H NMR spectrum for compound 8 recorded in $CDCl_3$ 4
    1.2. ^{13}C NMR spectrum for compound 8 recorded in $CDCl_3$ 5
    1.3. DEPT-135 spectrum for compound 8 recorded in $CDCl_3$ 6
2. NMR data for compound 9 7
    2.1. ^{1}H NMR spectrum for compound 9 recorded in $CDCl_3$ 7
    2.2. ^{13}C NMR spectrum for compound 9 recorded in $CDCl_3$ 8
    2.3. DEPT-135 spectrum for compound 8 recorded in $CDCl_3$ 9
3. NMR data for compound 10 10
    3.1. ^{1}H NMR spectrum for compound 10 recorded in $CDCl_3$ 10
    3.2. ^{13}C NMR spectrum for compound 10 recorded in $CDCl_3$ 11
    3.3. DEPT-135 spectrum for compound 10 recorded in $CDCl_3$ 12
4. NMR data for compound 12 13
    4.1. ^{1}H NMR spectrum for compound 12 recorded in $CDCl_3$ 13
    4.2. ^{13}C NMR spectrum for compound 12 recorded in $CDCl_3$ 14
    4.3. DEPT-135 spectrum for compound 12 recorded in $CDCl_3$ 15
    4.4. HMQC NMR spectrum for compound 12 recorded in $CDCl_3$ 16
    4.5. HMBC NMR spectrum for compound 12 recorded in $CDCl_3$ 17
5. NMR data for compound 14 18
    5.1. ^{1}H NMR spectrum for compound 14 recorded in $CDCl_3$ 18
    5.2. ^{13}C NMR spectrum for compound 14 recorded in $CDCl_3$ 19
    5.3. DEPT-135 spectrum for compound 14 recorded in $CDCl_3$ 20
6. NMR data for compound 16 21
    6.1. ^{1}H NMR spectrum for compound 16 recorded in $CDCl_3$ 21
    6.2. ^{13}C NMR spectrum for compound 16 recorded in $CDCl_3$ 22
    6.3. DEPT-135 spectrum for compound 16 recorded in $CDCl_3$ 23
7. NMR data for compound 17 24
    7.1. ^{1}H NMR spectrum for compound 17 recorded in $CDCl_3$ 24
    7.2. ^{13}C NMR spectrum for compound 17 recorded in $CDCl_3$ 25
    7.3. DEPT-135 spectrum for compound 17 recorded in $CDCl_3$ 26
    7.4. HMQC NMR spectrum for compound 17 recorded in $CDCl_3$ 27
    7.5. HMCB NMR spectrum for compound 17 in $CDCl_3$ 28
8. NMR data for compound 19 29
    8.1. ^{1}H NMR spectrum for compound 19 recorded in $CDCl_3$ 29
    8.2. ^{13}C NMR spectrum for compound 19 recorded in $CDCl_3$ 30

8.3. DEPT-135 spectrum for compound 19 recorded in $CDCl_3$ 31
9. NMR data for compound 20 32
9.1. ^{1}H NMR spectrum for compound 20 recorded in $CDCl_3$ 32
9.2. ^{13}C NMR spectrum for compound 20 recorded in $CDCl_3$ 33
9.3. DEPT-135 spectrum for compound 20 recorded in $CDCl_3$ 34
10. NMR data for compound 21 35
10.1. ^{1}H NMR spectrum for compound 21 recorded in $CDCl_3$ 35
10.2. ^{13}C NMR spectrum for compound 21 recorded in $CDCl_3$ 36
10.3. DEPT-135 spectrum for compound 21 recorded in $CDCl_3$ 37
11. NMR data for compound 22 38
11.1. ^{1}H NMR spectrum for compound 22 recorded in $CDCl_3$ 38
11.2. ^{13}C NMR spectrum for compound 22 recorded in $CDCl_3$ 39
11.3. DEPT-135 spectrum for compound 22 recorded in $CDCl_3$ 40
11.4. HMQC NMR spectrum for compound 22 recorded in $CDCl_3$ 41
11.5. HMBC NMR spectrum for compound 22 recorded in $CDCl_3$ 42

1. NMR data for compound 8

1.1. ^{1}H NMR spectrum for compound 8 recorded in $CDCl_3$

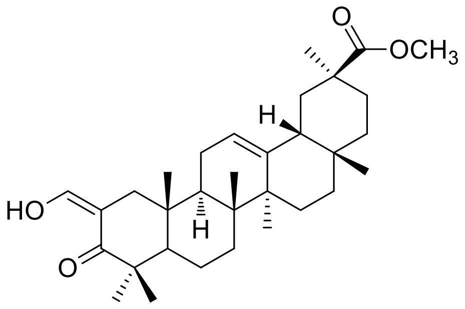

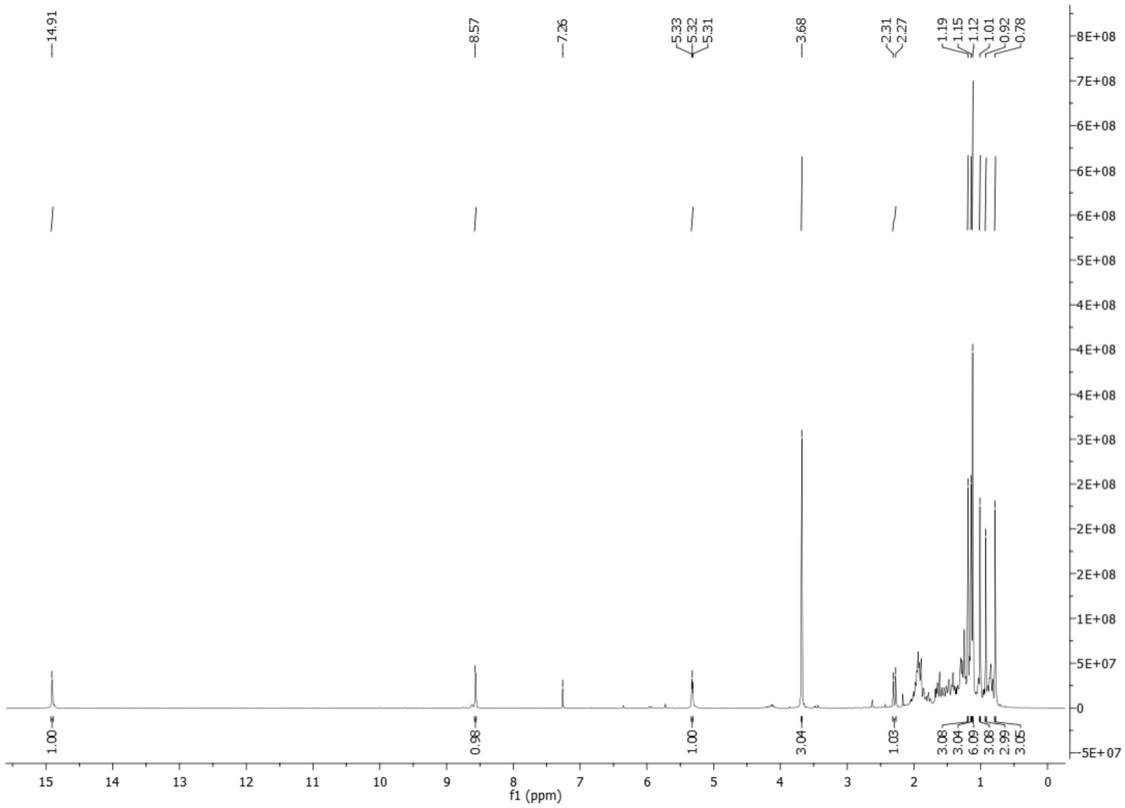

1.2. ^{13}C NMR spectrum for compound 8 recorded in $CDCl_3$

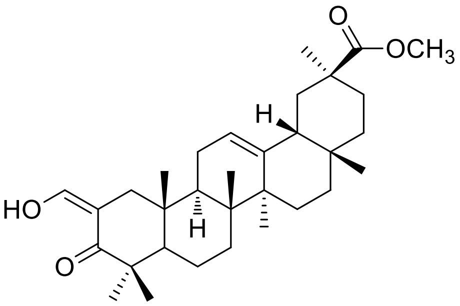

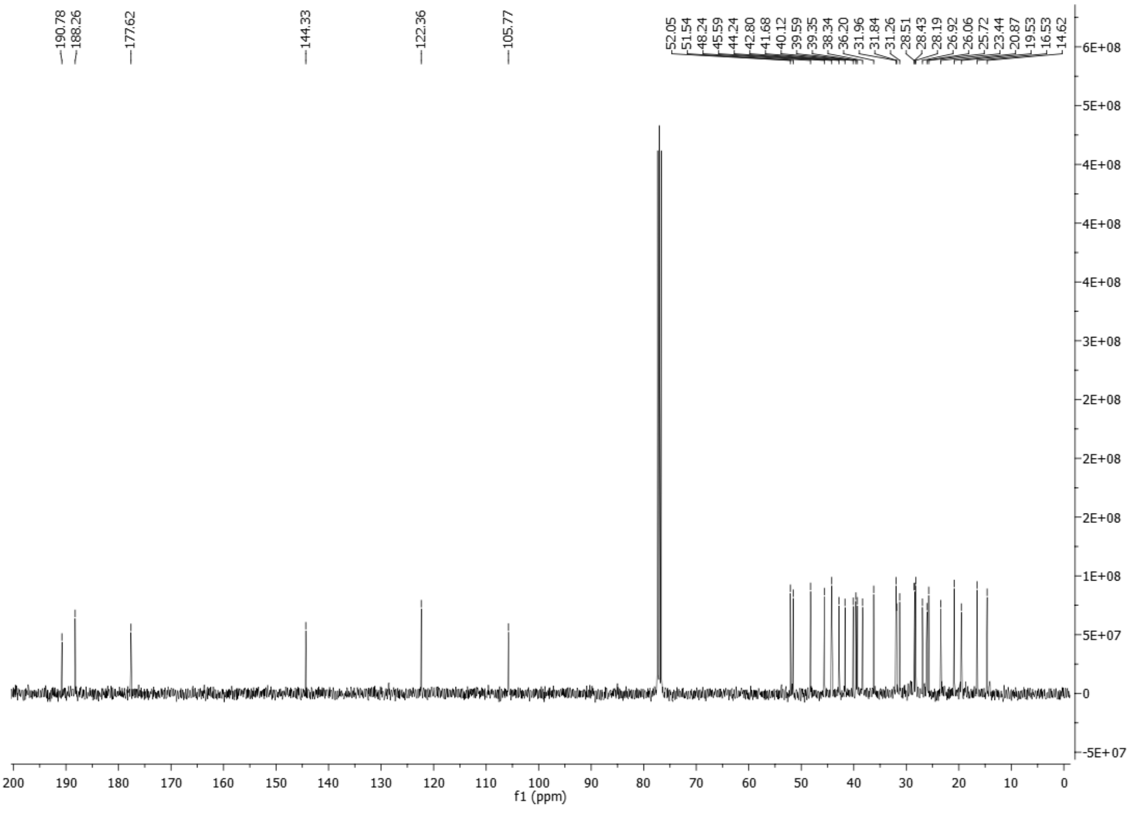

1.3. DEPT-135 spectrum for compound 8 recorded in $CDCl_3$

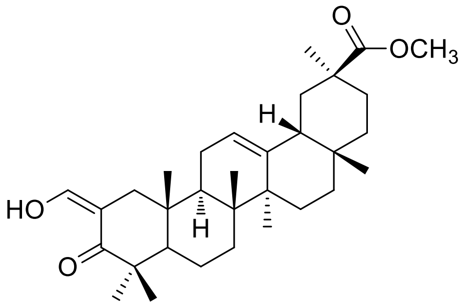

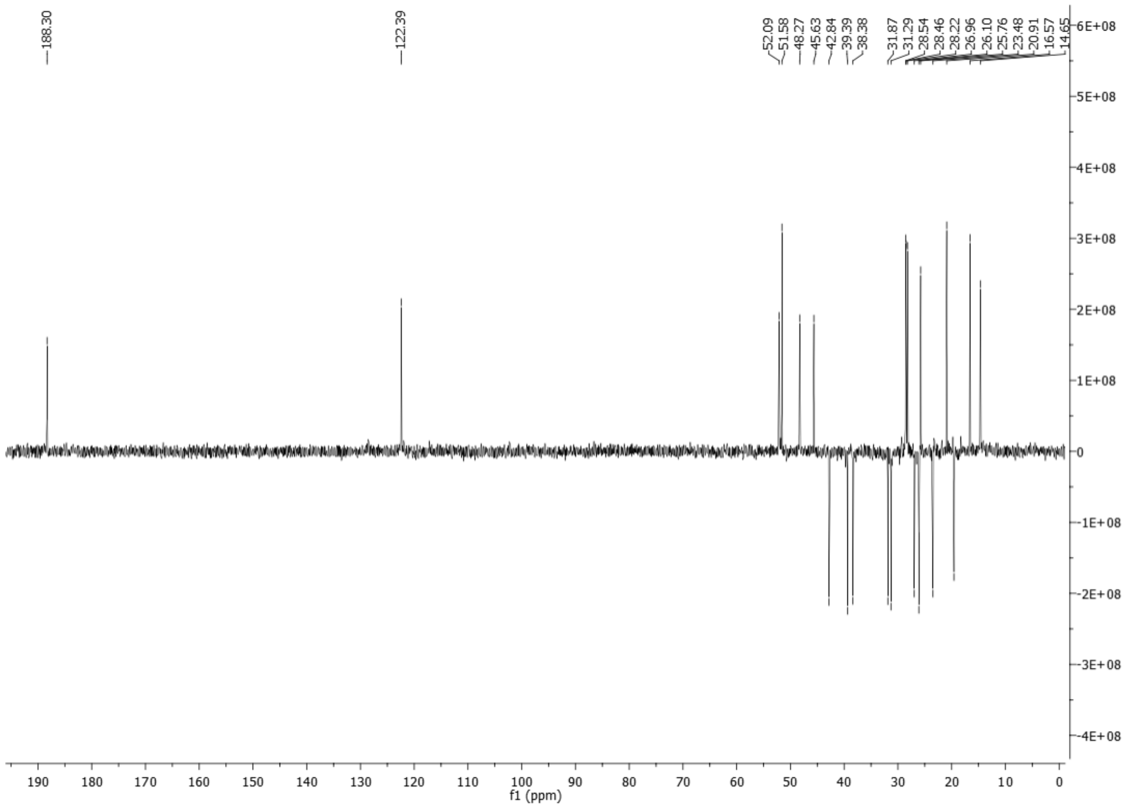

2. NMR data for compound 9

2.1. ^{1}H NMR spectrum for compound 9 recorded in $CDCl_3$

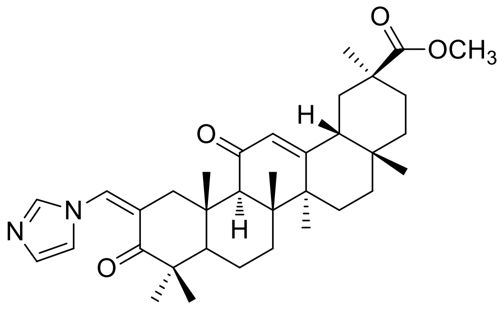

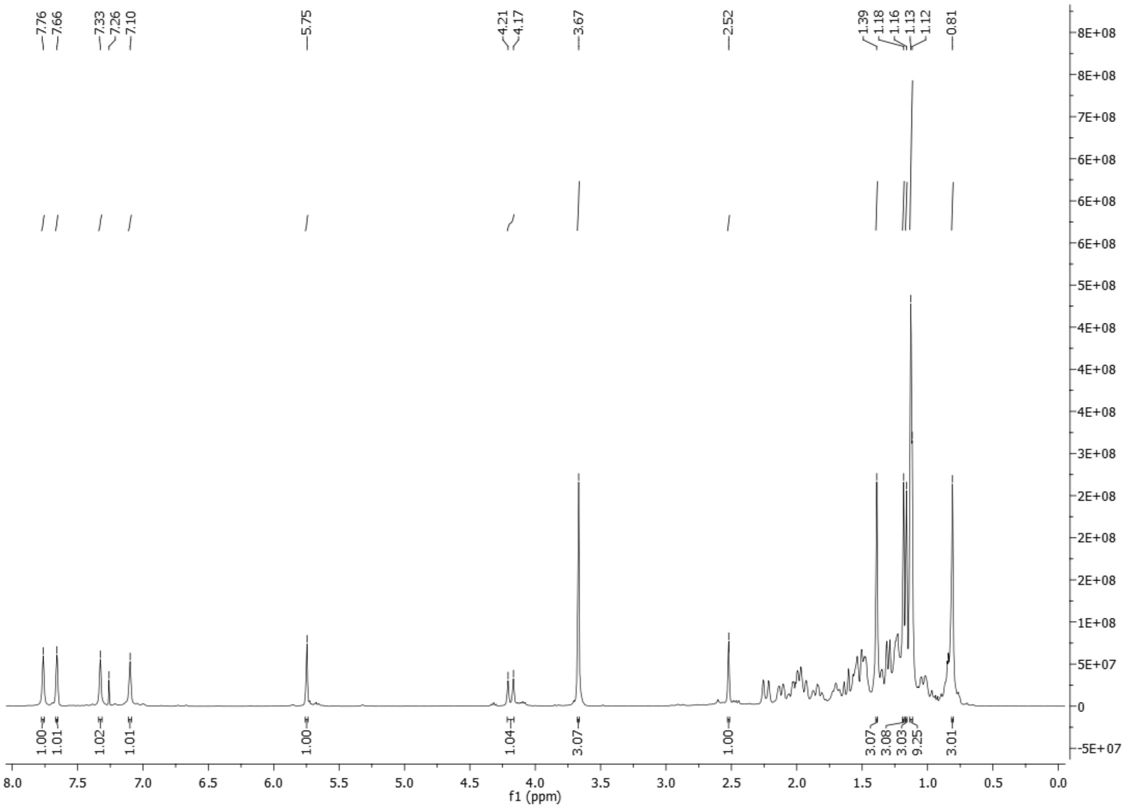

2.2. ^{13}C NMR spectrum for compound 9 recorded in $CDCl_3$

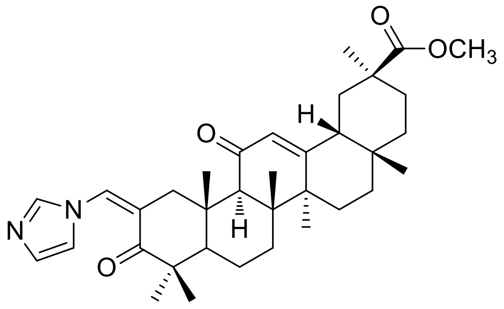

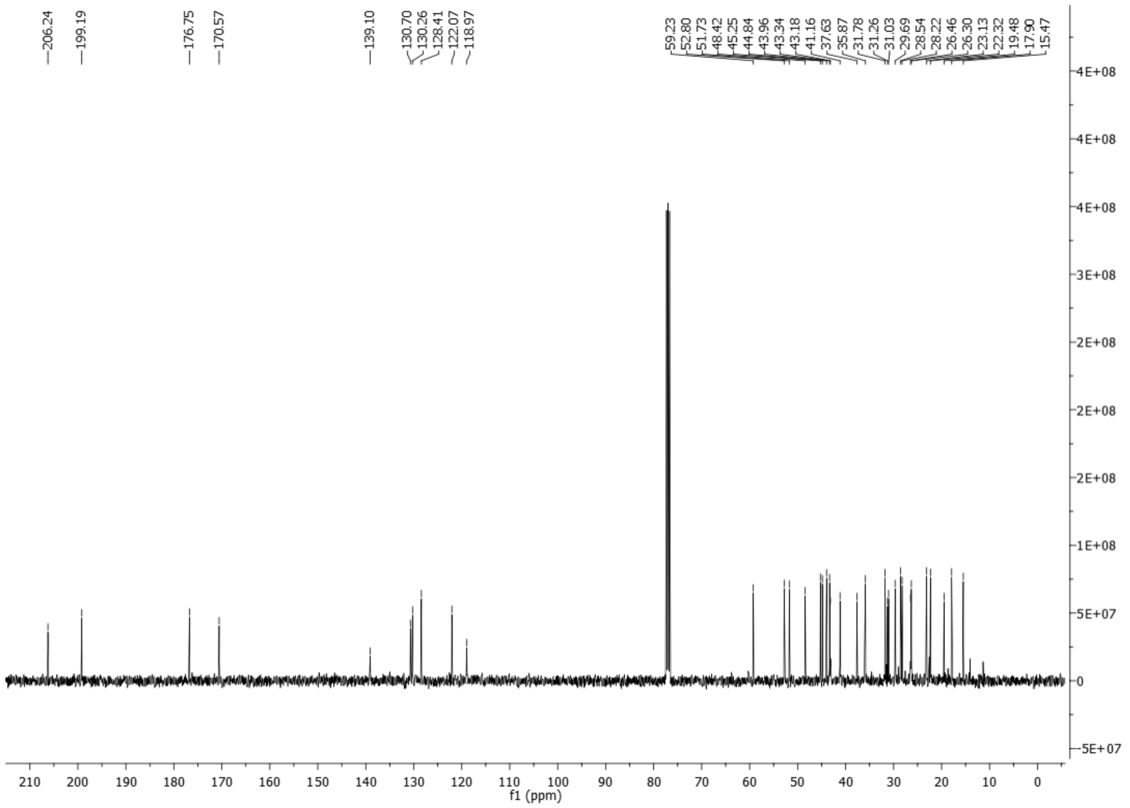

2.3. DEPT-135 spectrum for compound 9 recorded in $CDCl_3$

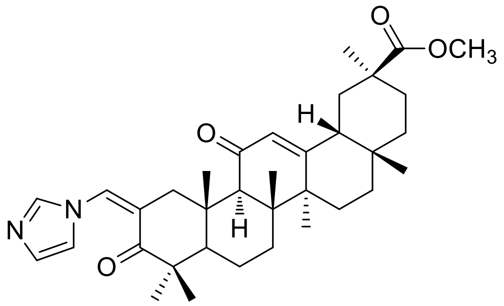

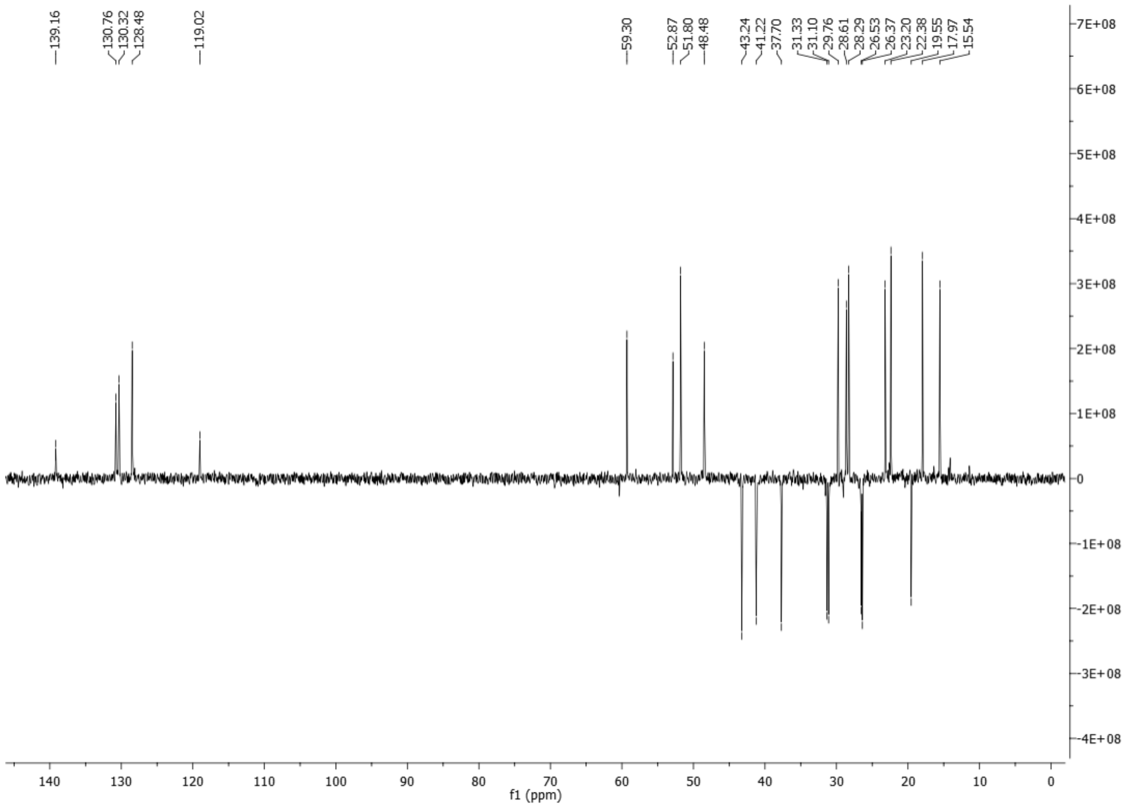

3. NMR data for compound 10

3.1. ^{1}H NMR spectrum for compound 10 recorded in $CDCl_3$

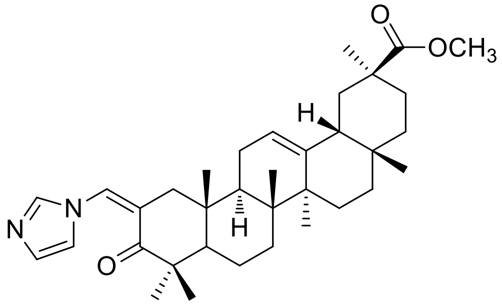

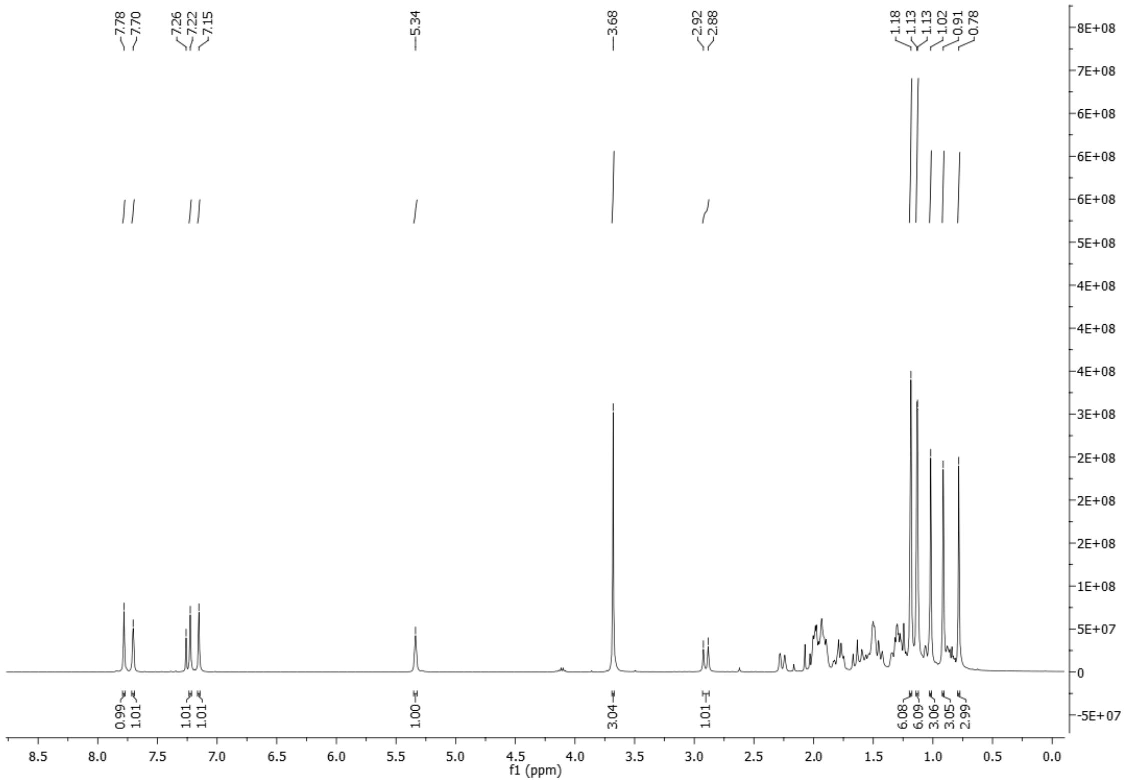

3.2. ^{13}C NMR spectrum for compound 10 recorded in $CDCl_3$

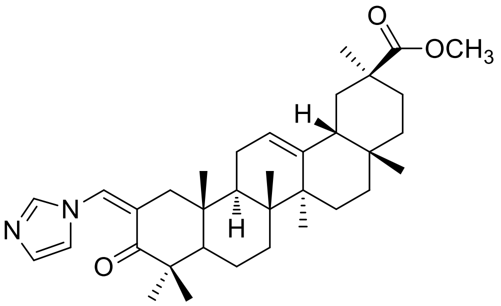

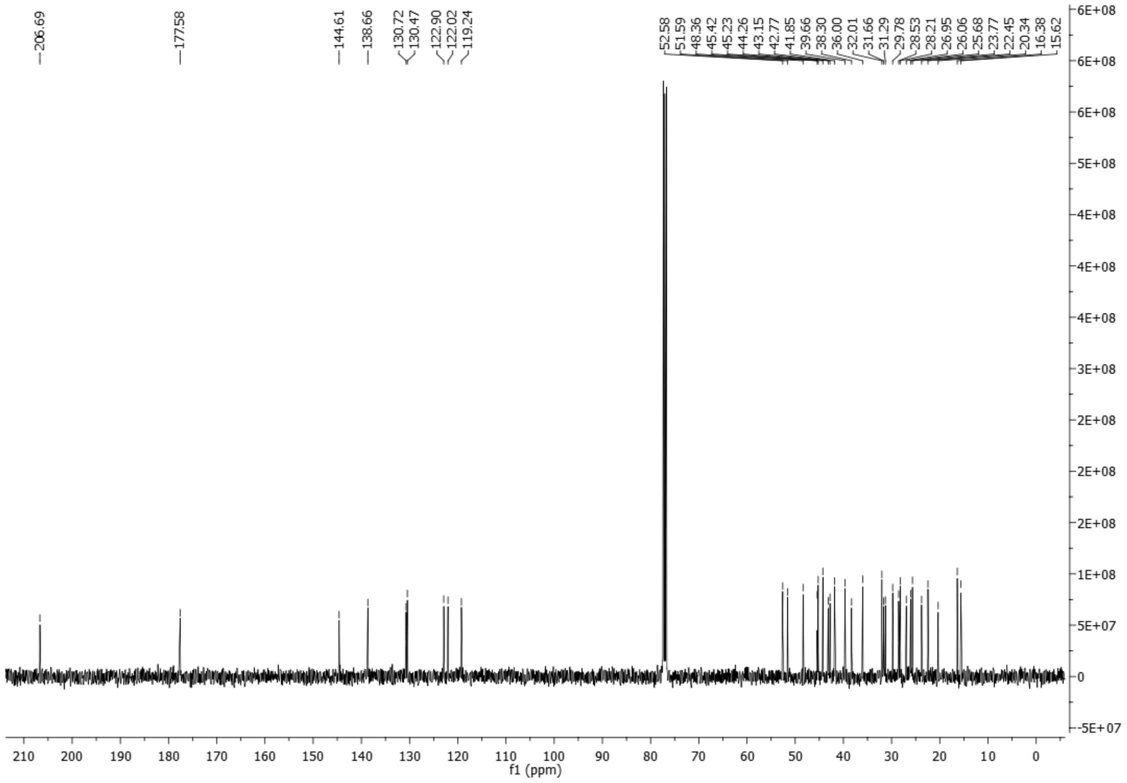

3.3.	DEPT-135 spectrum for compound 10 recorded in $CDCl_3$

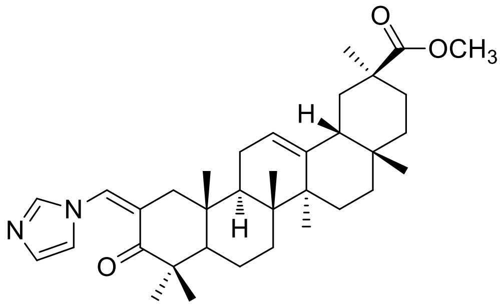

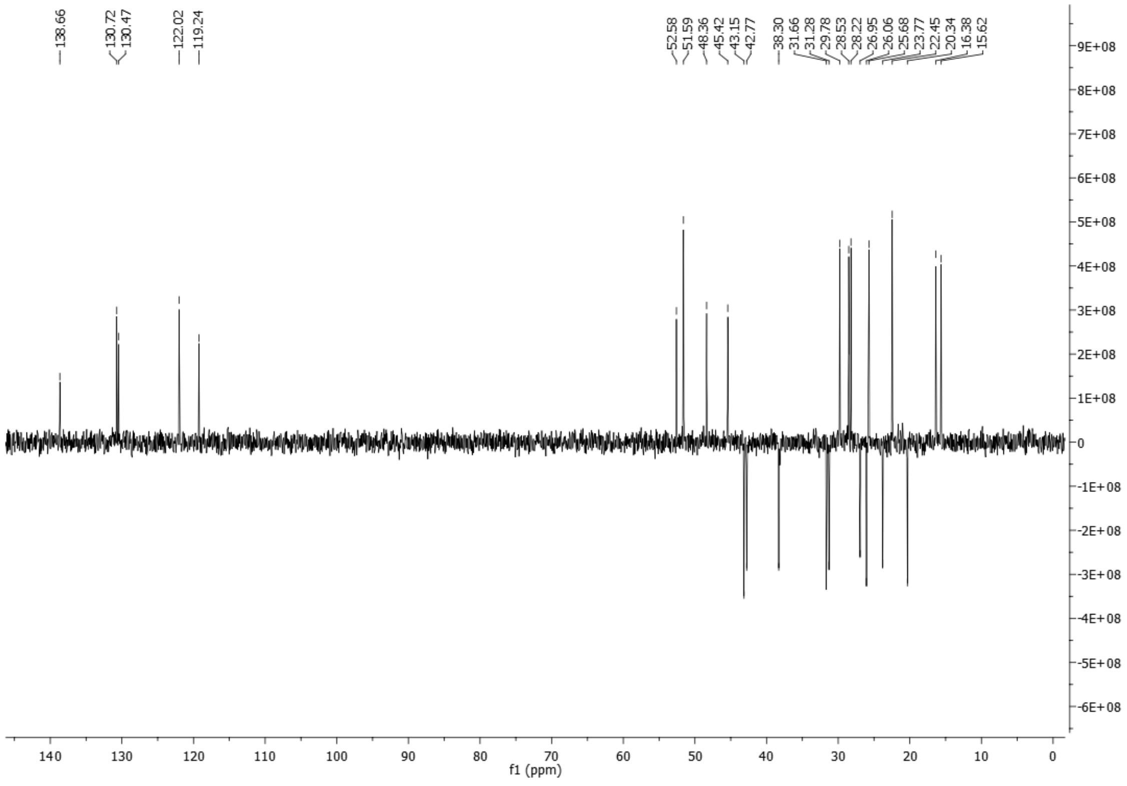

4. NMR data for compound 12

4.1. ^{1}H NMR spectrum for compound 12 recorded in $CDCl_3$

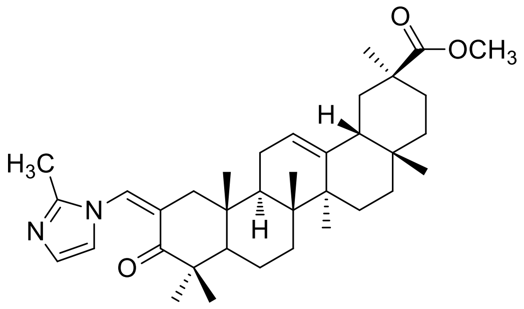

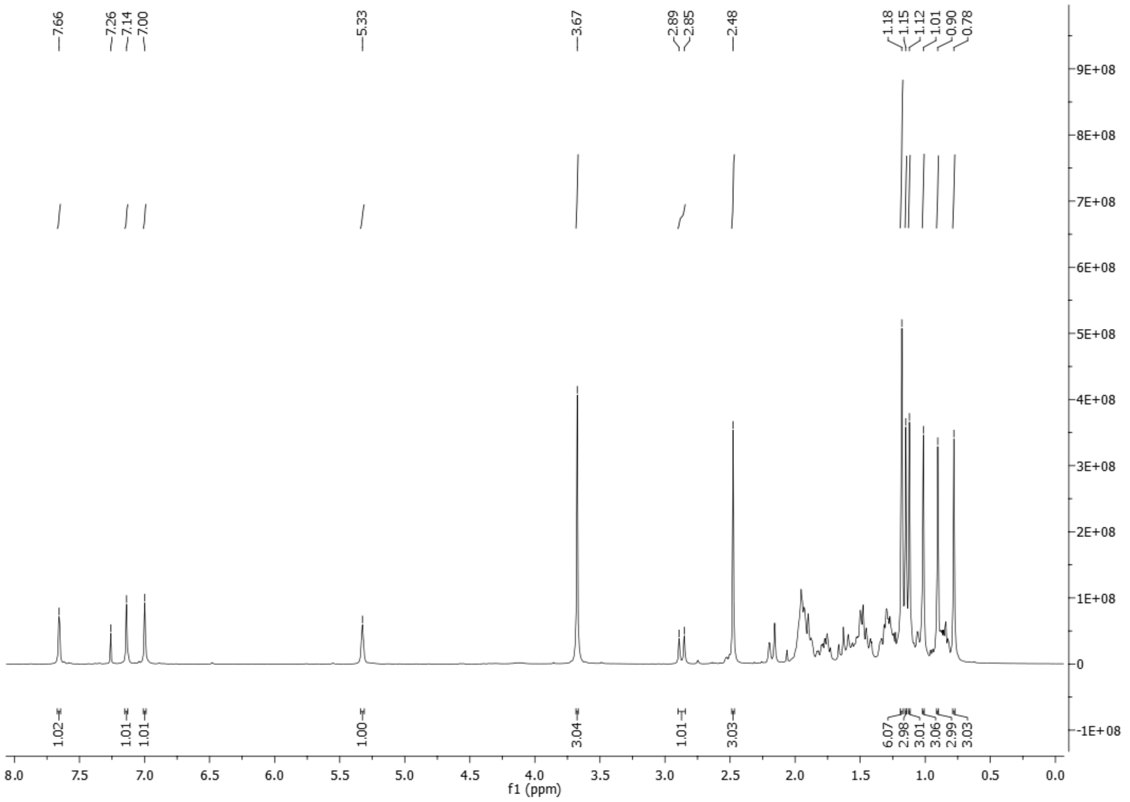

4.2. ^{13}C NMR spectrum for compound 12 recorded in $CDCl_3$

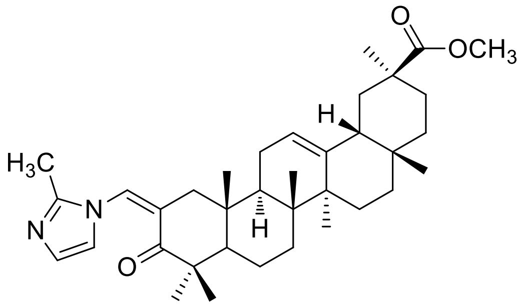

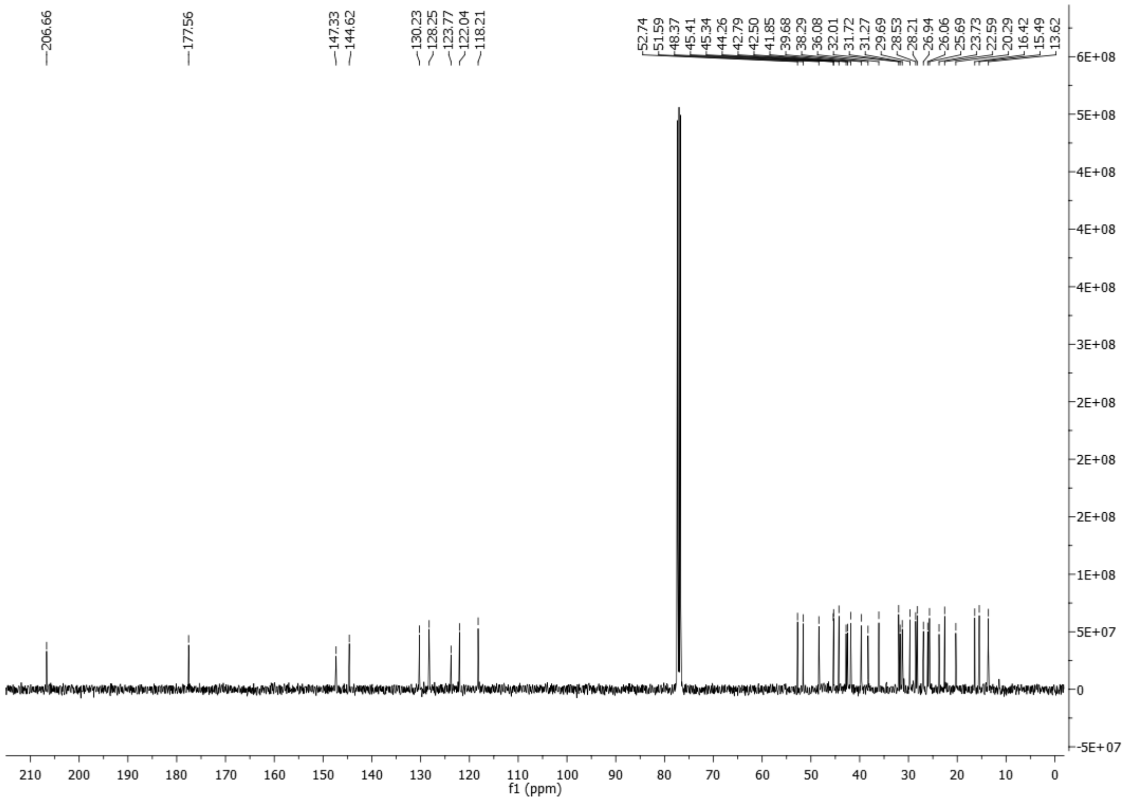

4.3. DEPT-135 spectrum for compound 12 recorded in $CDCl_3$

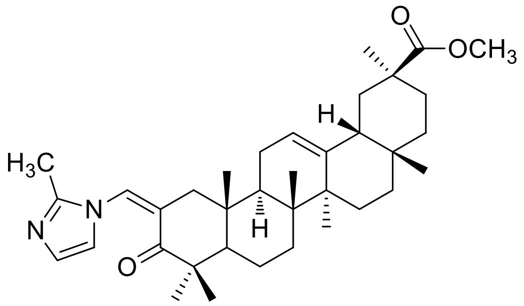

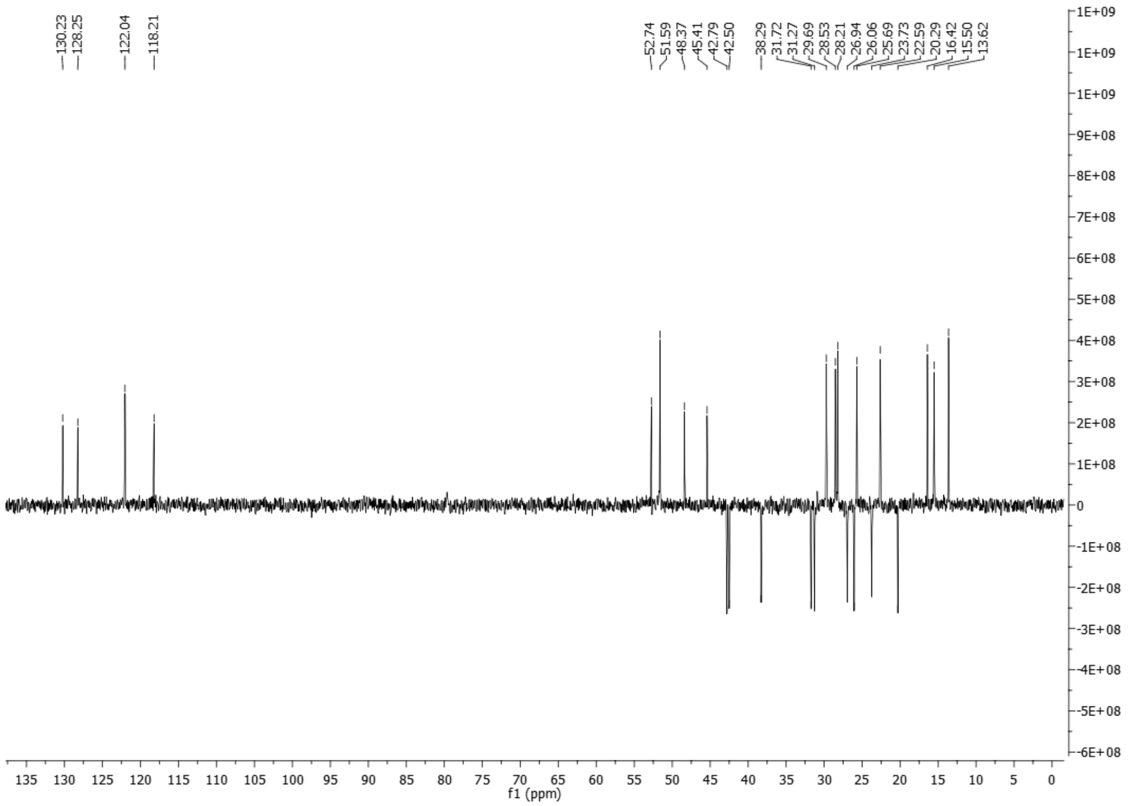

4.4. HMQC NMR spectrum for compound 12 recorded in $CDCl_3$

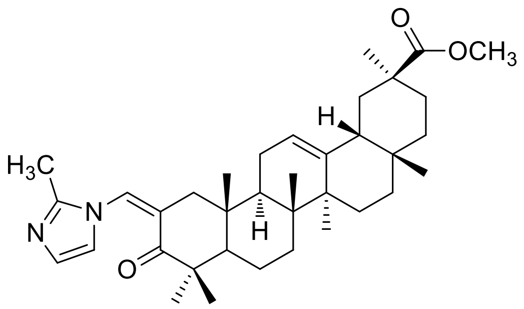

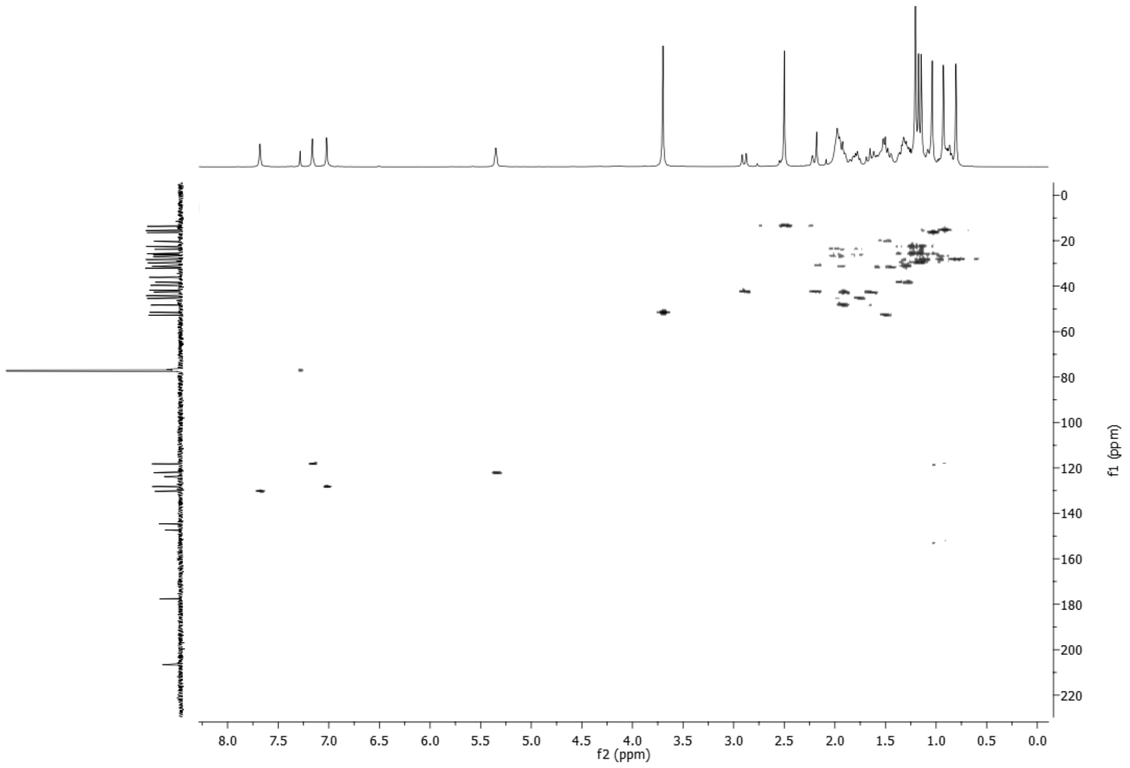

4.5. HMBC NMR spectrum for compound 12 recorded in $CDCl_3$

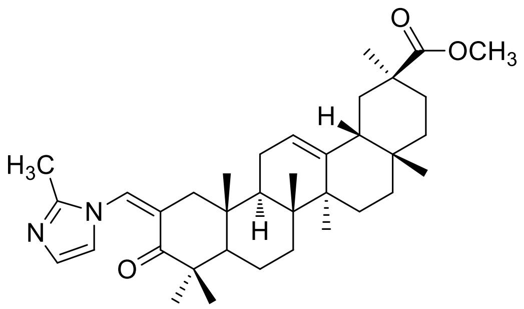

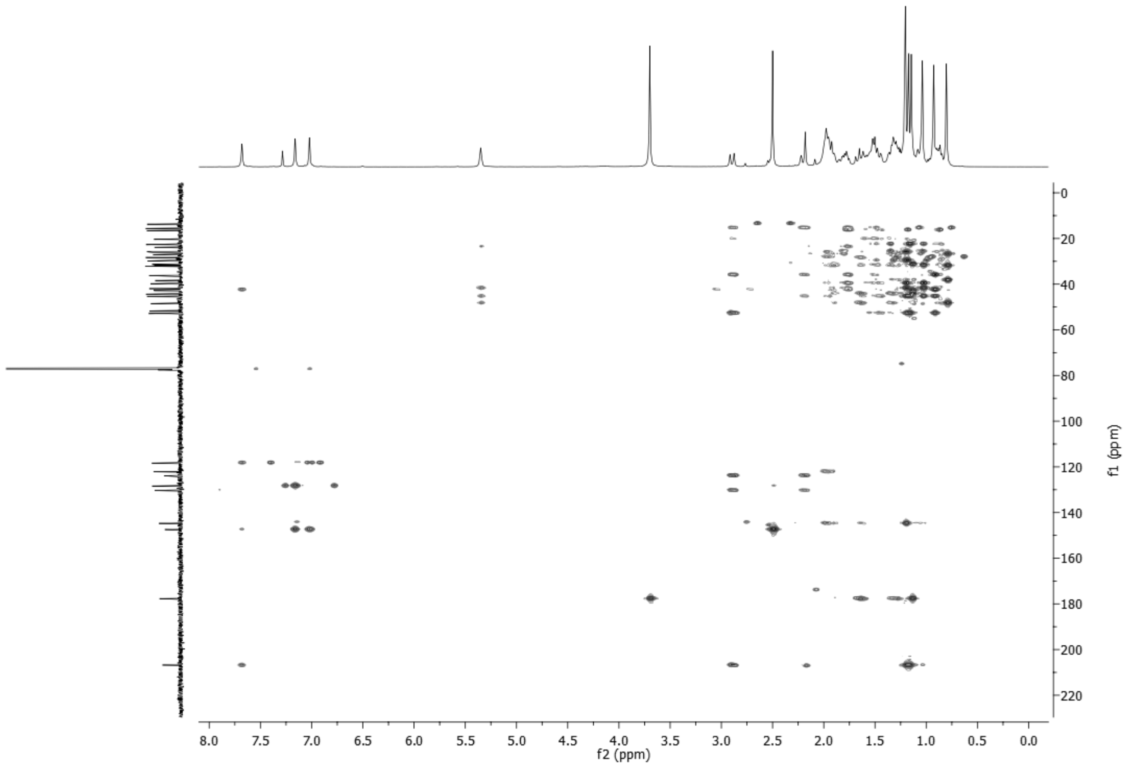

5. NMR data for compound 14

5.1. ^{1}H NMR spectrum for compound 14 recorded in $CDCl_3$

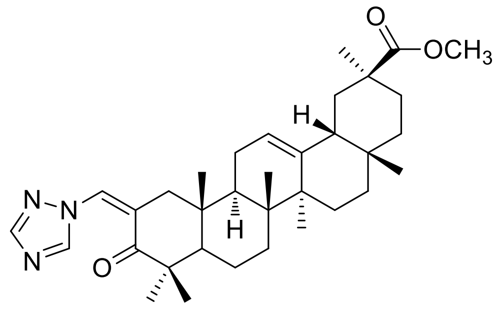

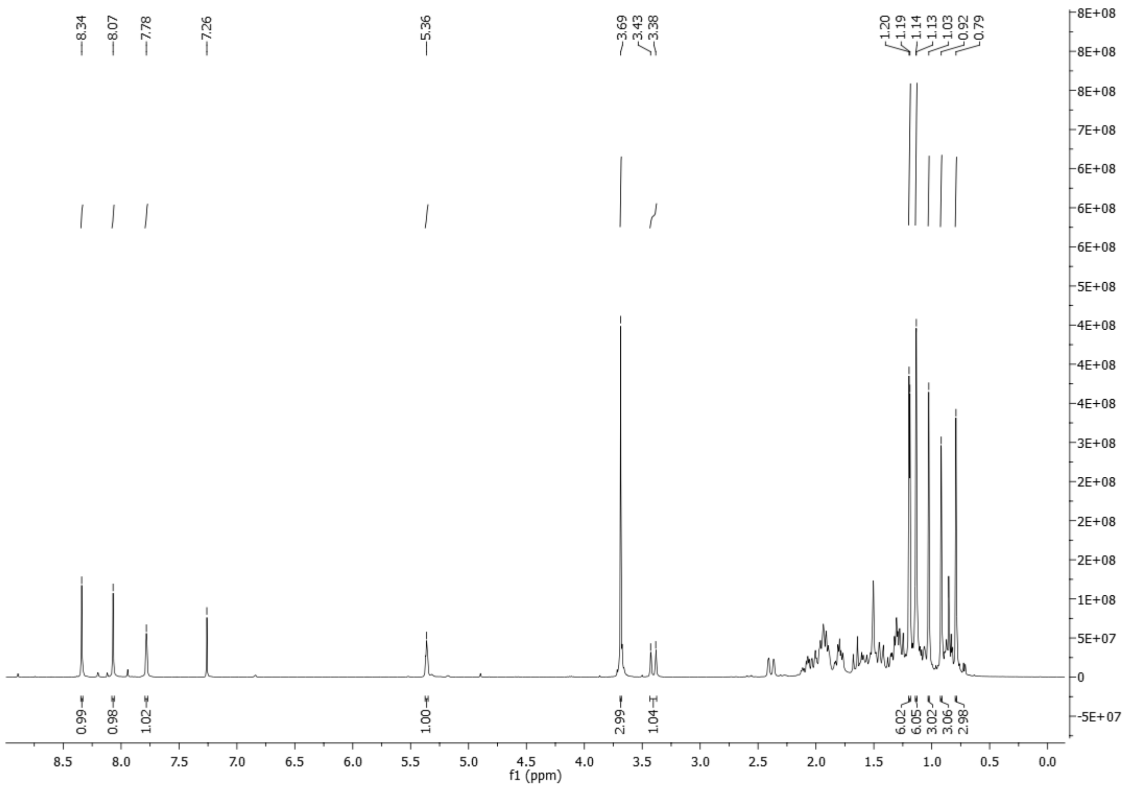

5.2. ^{13}C NMR spectrum for compound 14 recorded in $CDCl_3$

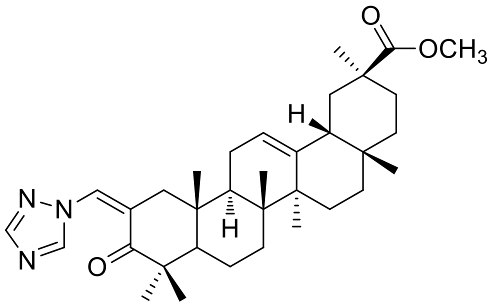

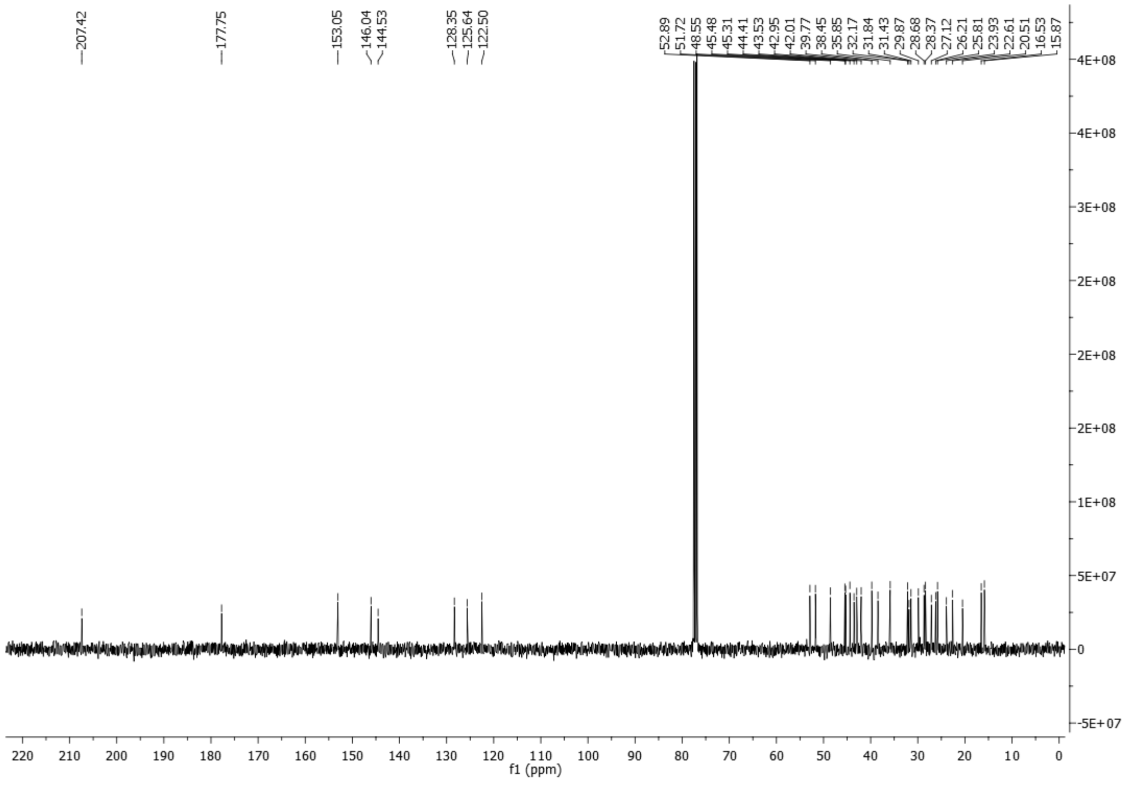

5.3. DEPT-135 spectrum for compound 14 recorded in $CDCl_3$

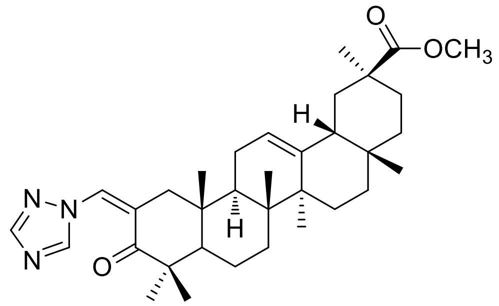

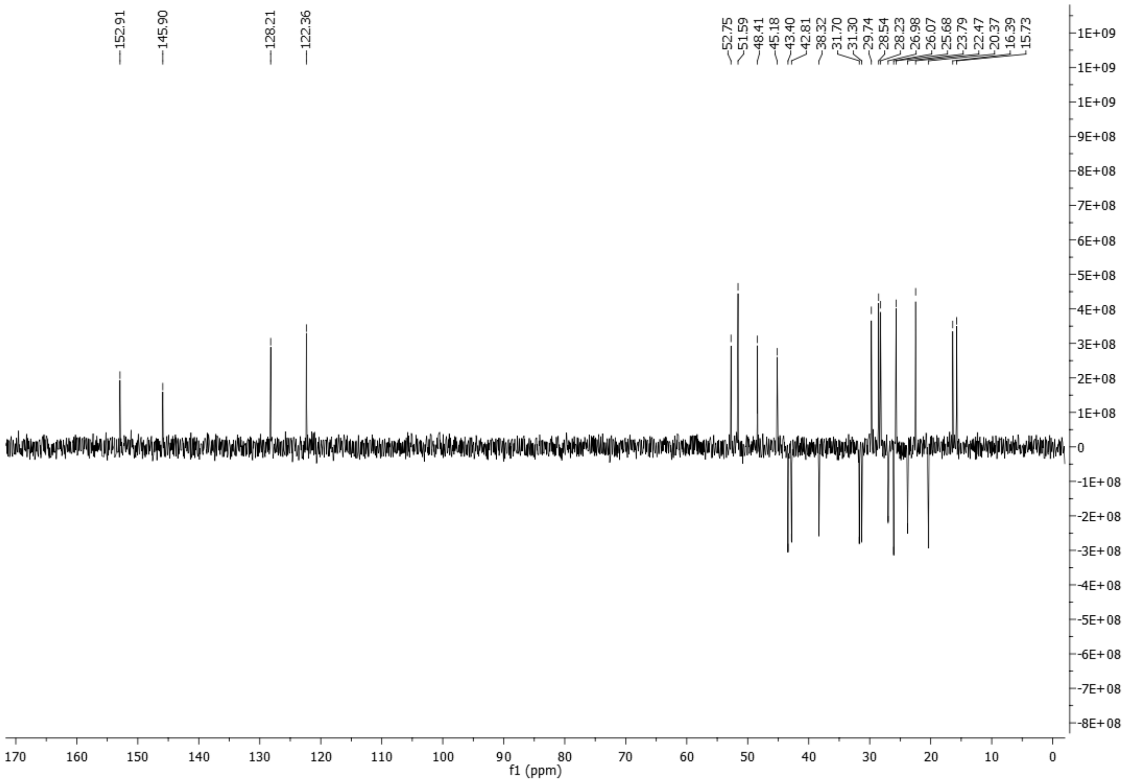

6. NMR data for compound 16

6.1. ^{1}H NMR spectrum for compound 16 recorded in $CDCl_3$

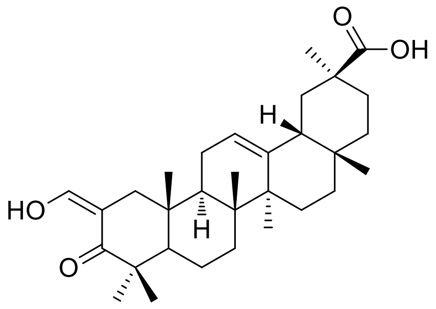

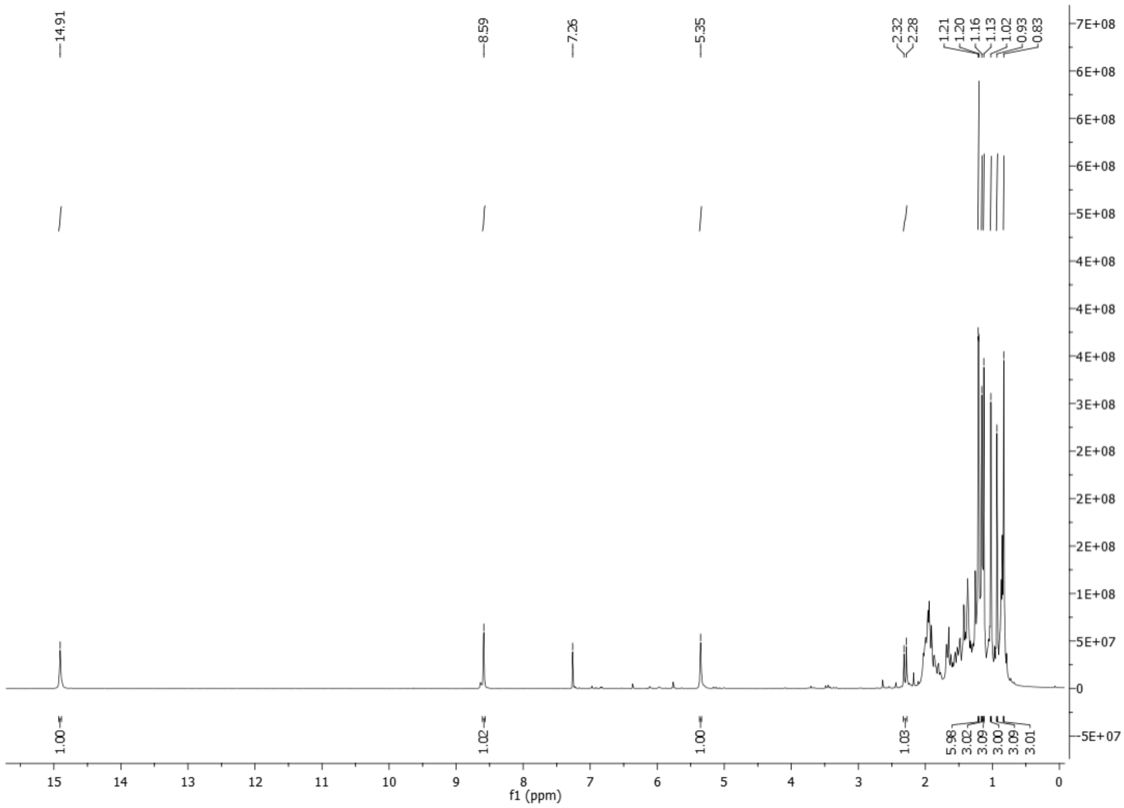

6.2. ^{13}C NMR spectrum for compound 16 recorded in $CDCl_3$

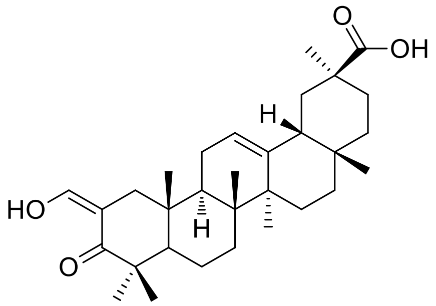

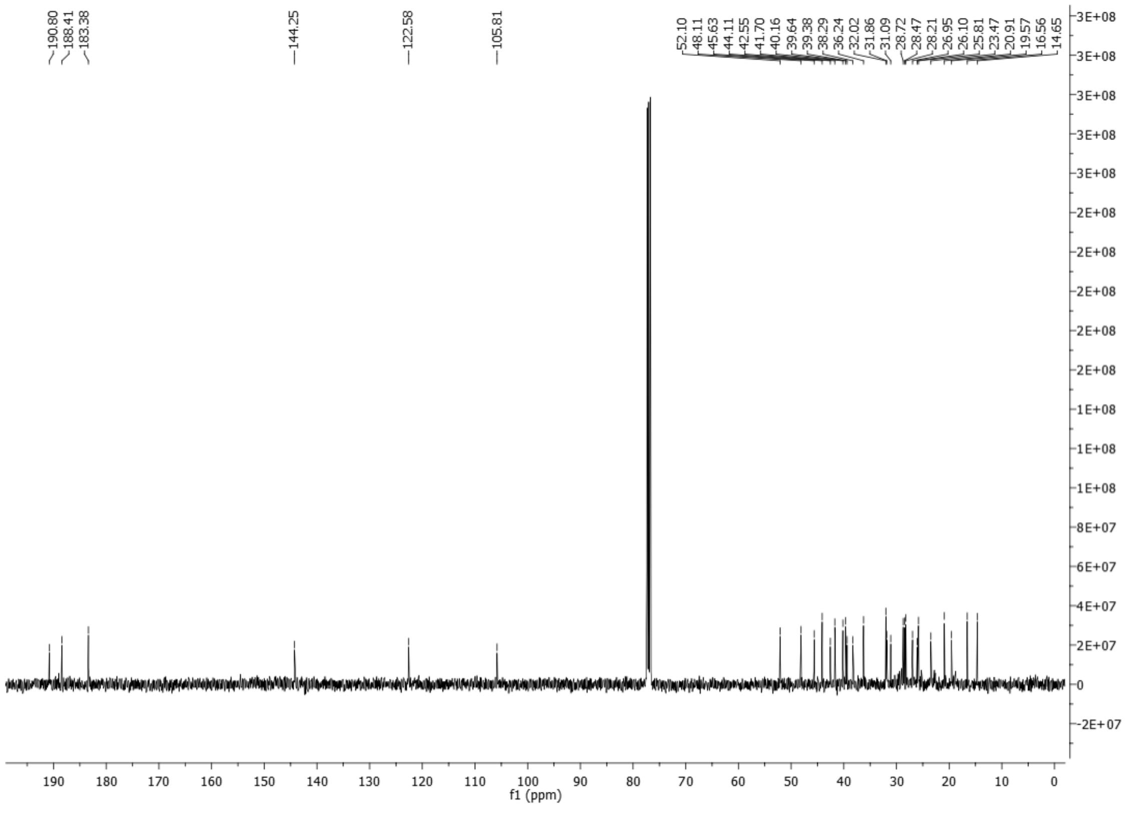

6.3. DEPT-135 spectrum for compound 16 recorded in $CDCl_3$

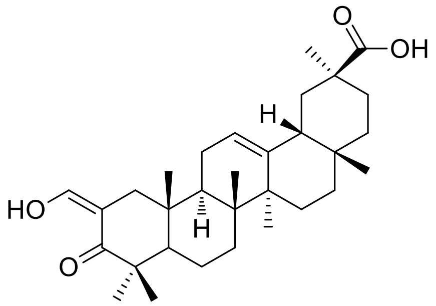

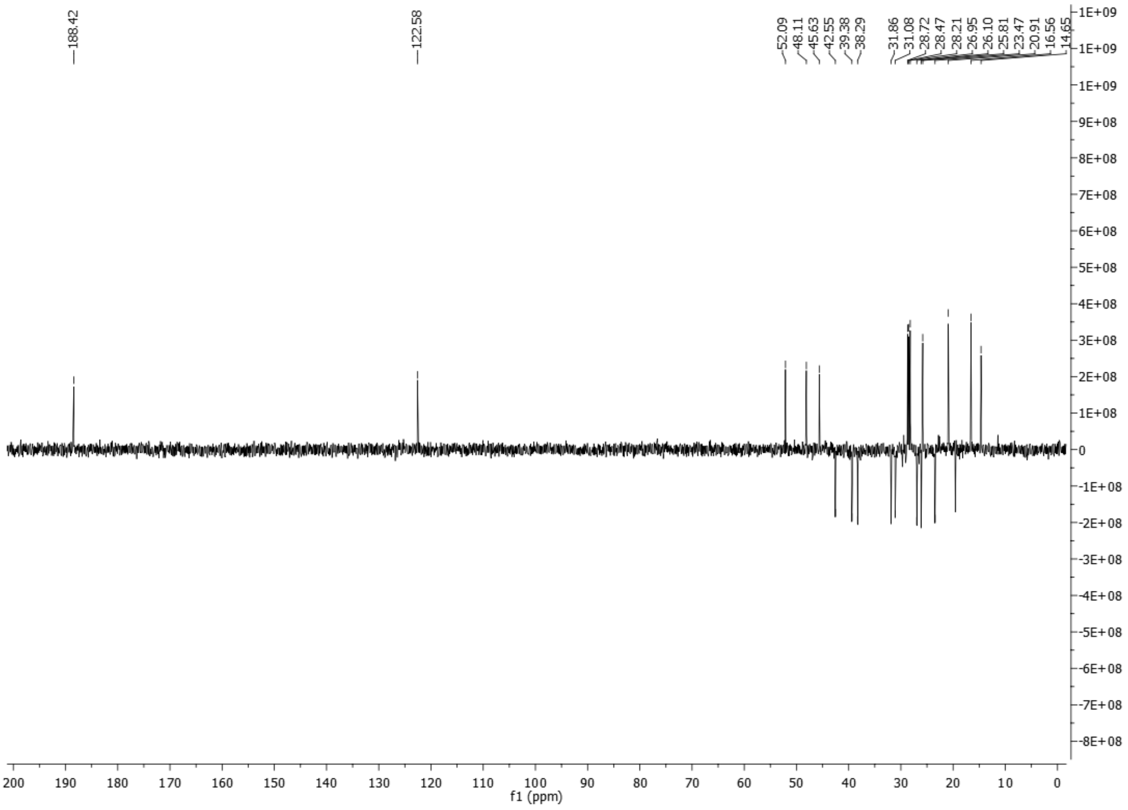

7. NMR data for compound 17

7.1. ^{1}H NMR spectrum for compound 17 recorded in $CDCl_3$

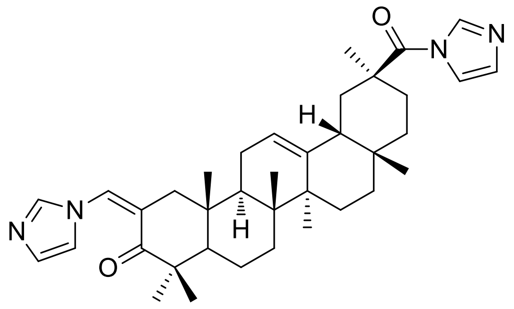

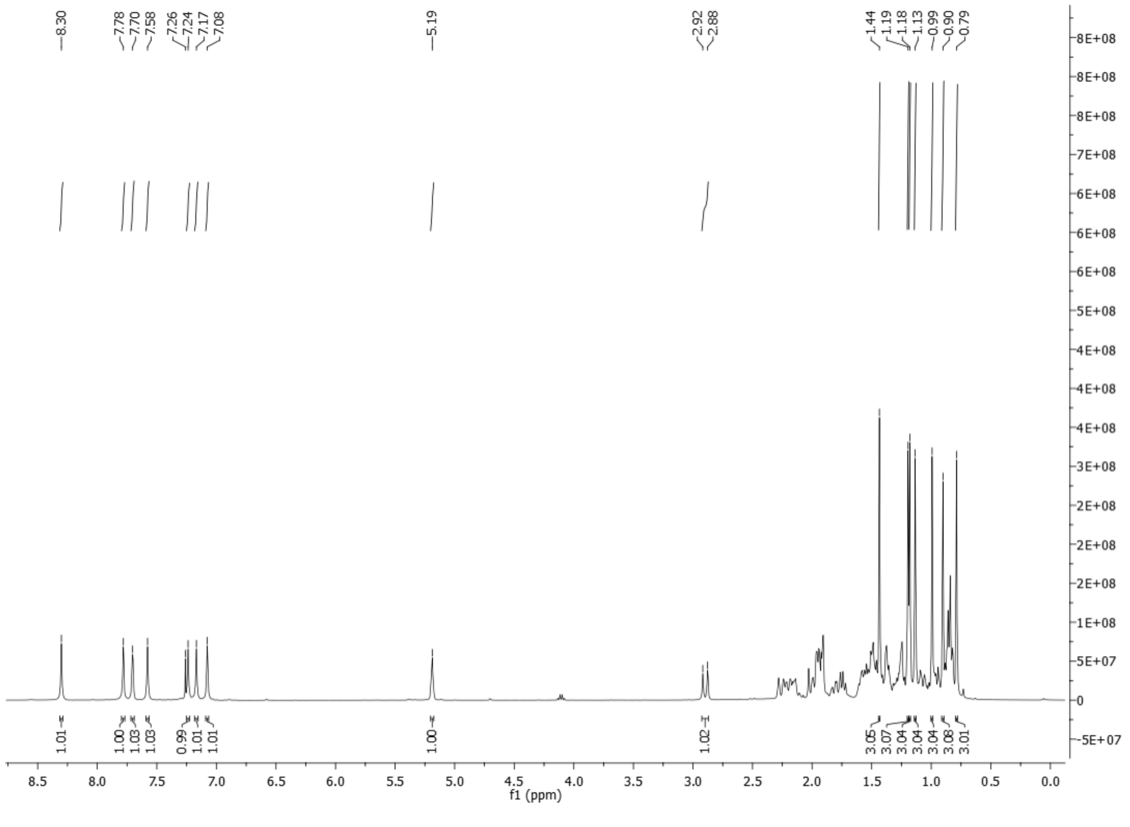

7.2. ^{13}C NMR spectrum for compound 17 recorded in $CDCl_3$

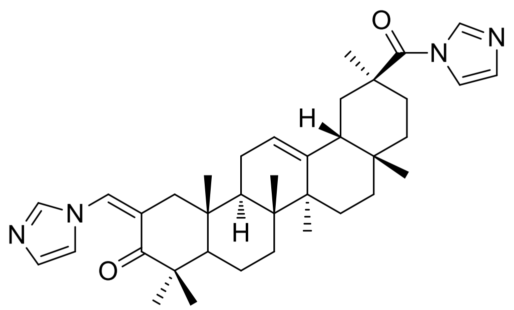

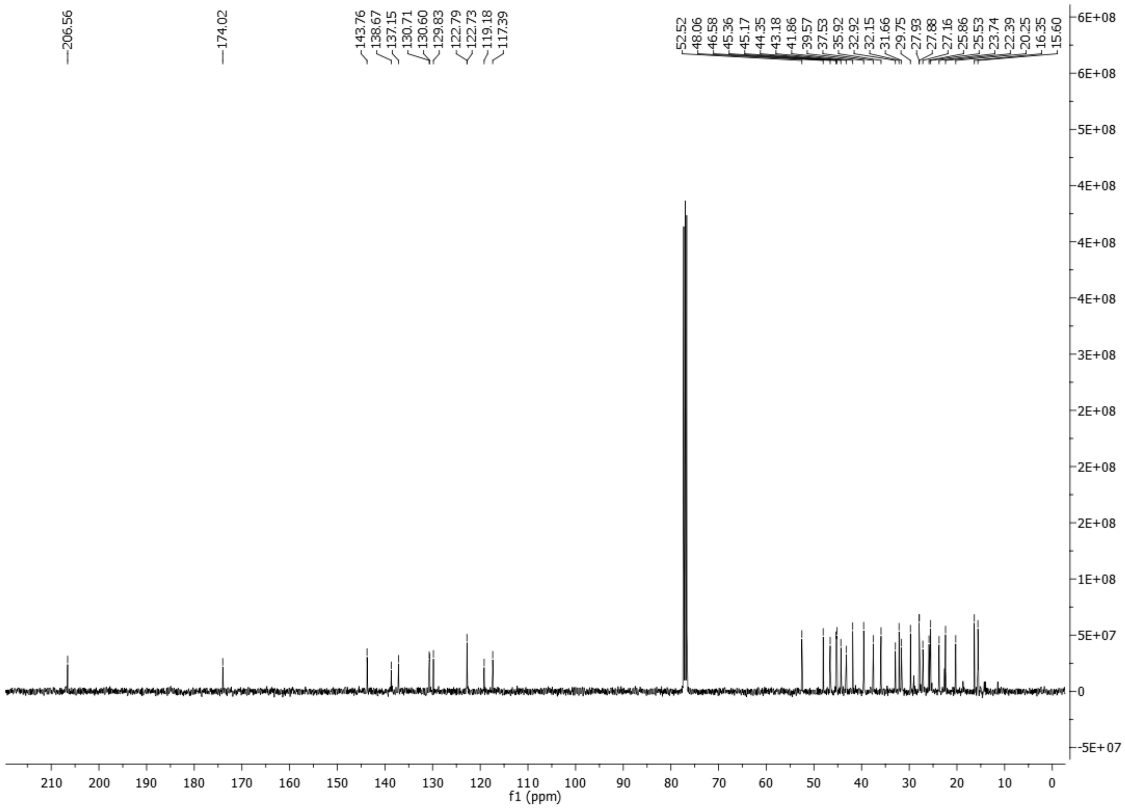

7.3. DEPT-135 spectrum for compound 17 recorded in $CDCl_3$

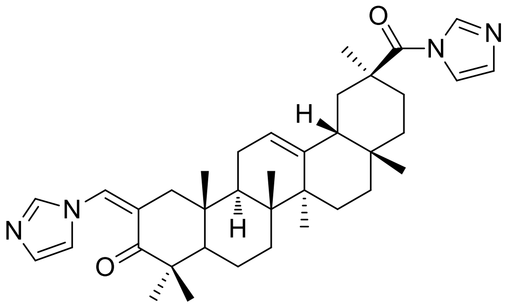

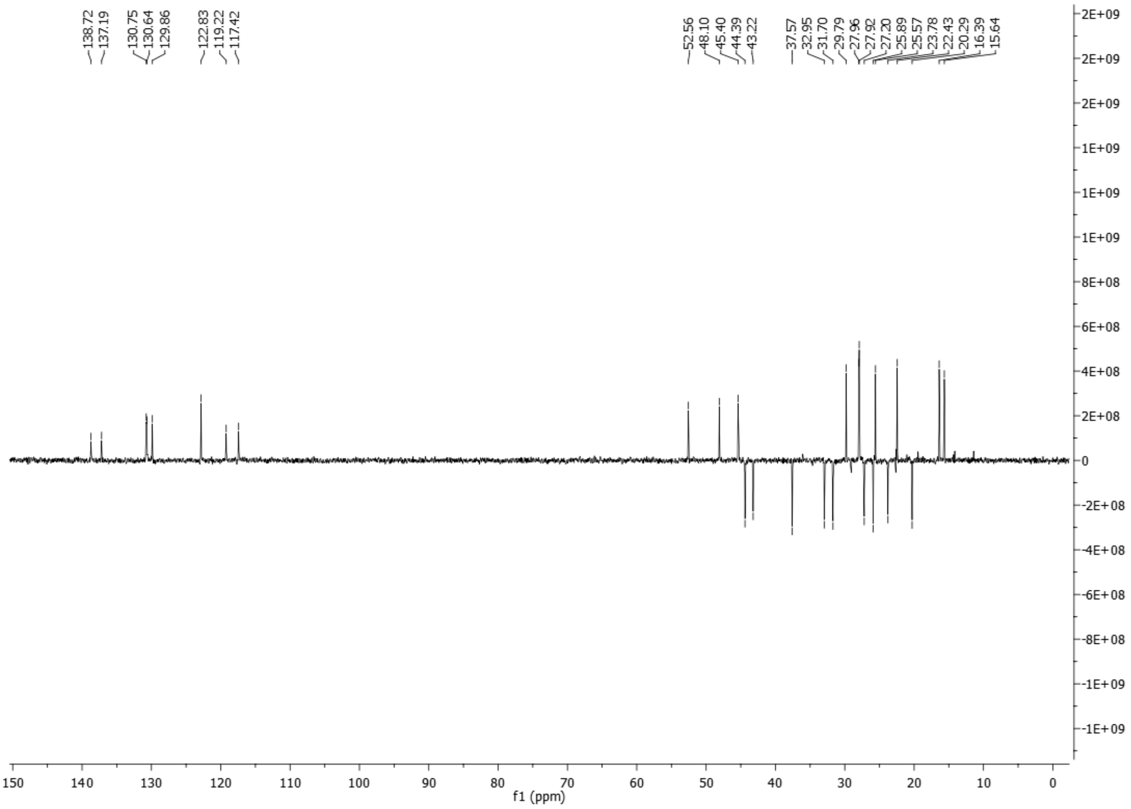

7.4. HMQC NMR spectrum for compound 17 recorded in $CDCl_3$

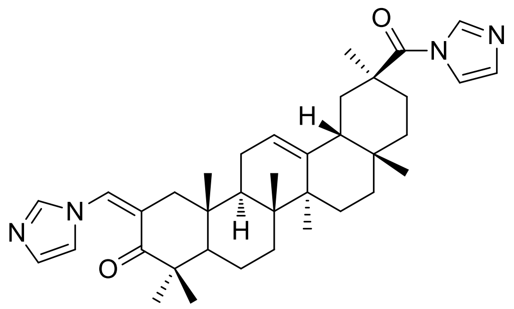

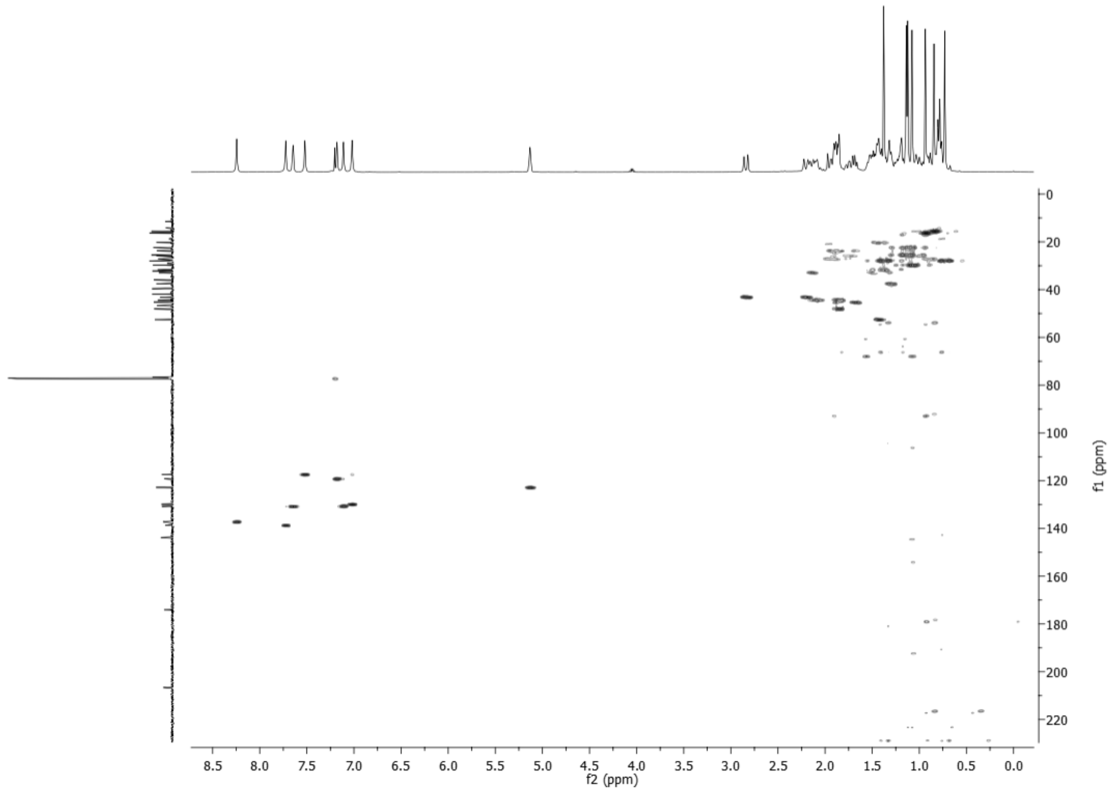

7.5. HMCB NMR spectrum for compound 17 in $CDCl_3$

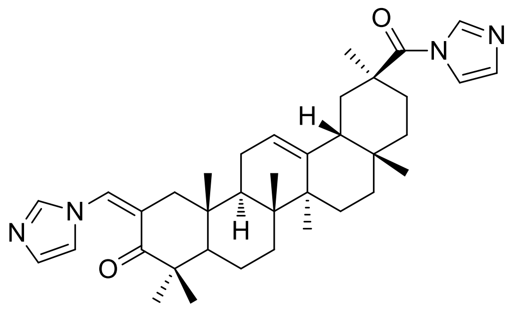

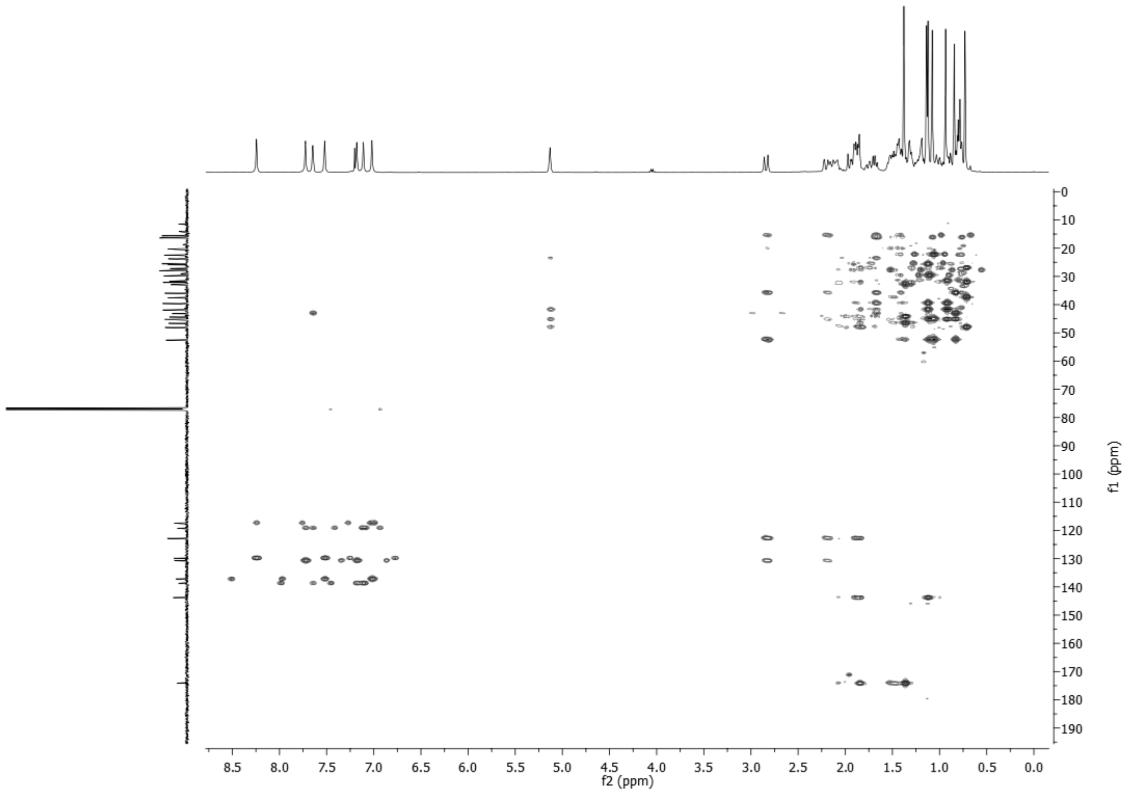

8. NMR data for compound 19

8.1. ^{1}H NMR spectrum for compound 19 recorded in $CDCl_3$

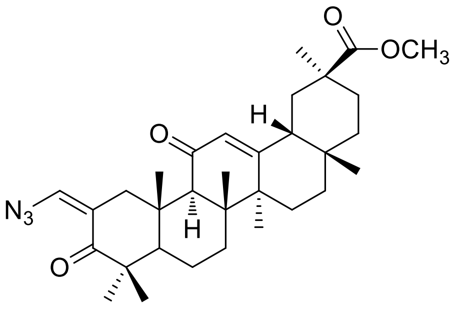

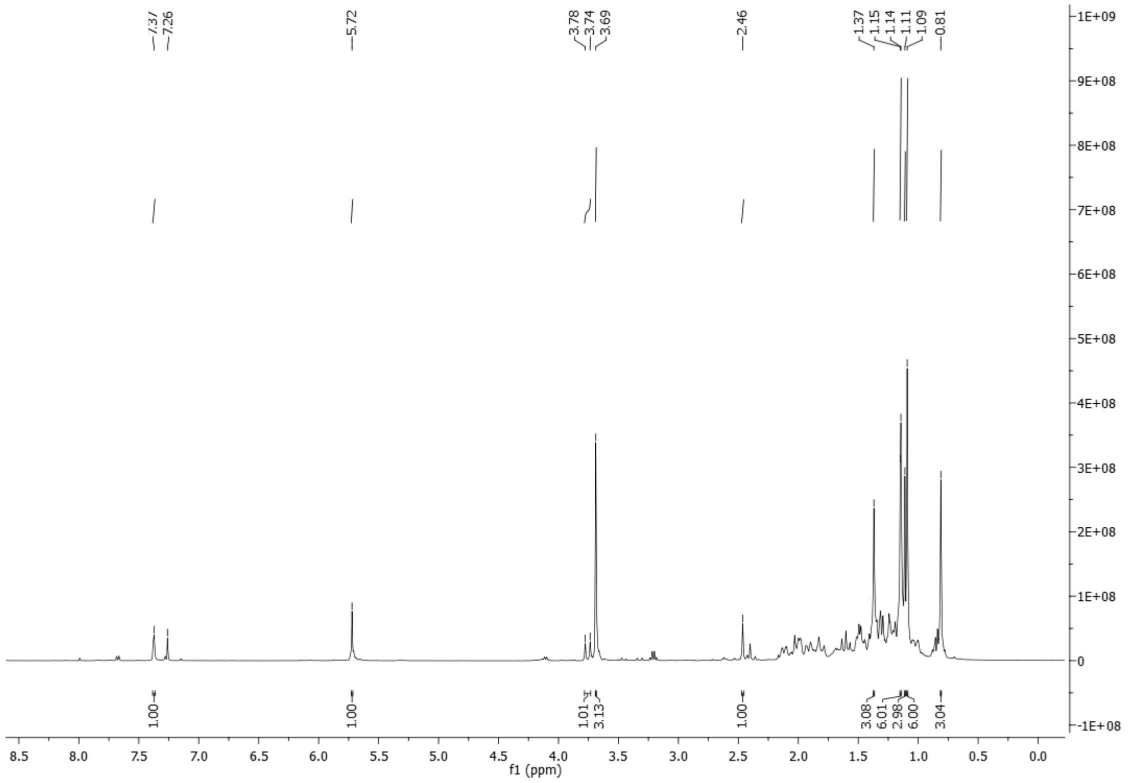

8.2. ^{13}C NMR spectrum for compound 19 recorded in $CDCl_3$

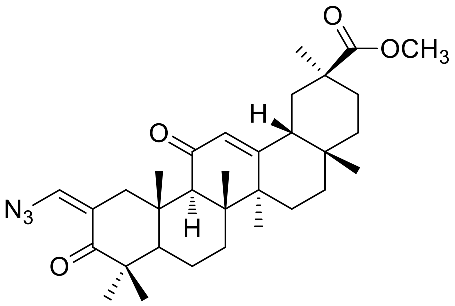

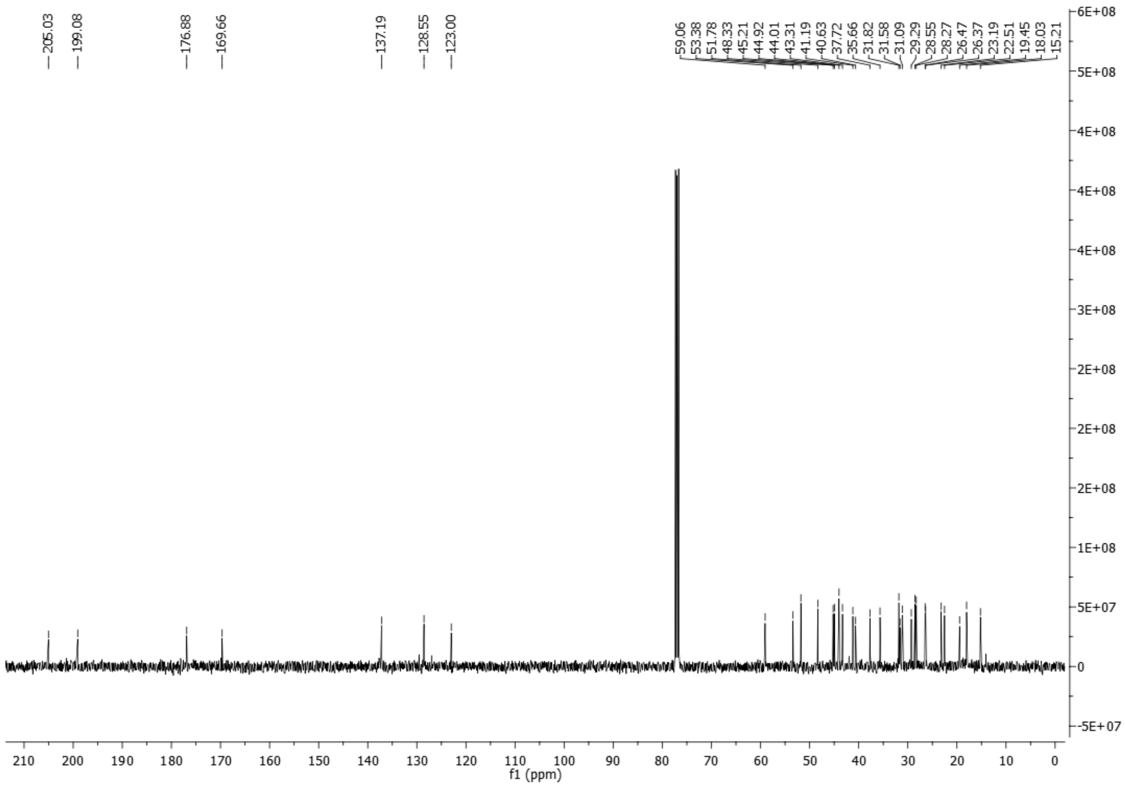

8.3. DEPT-135 spectrum for compound 19 recorded in $CDCl_3$

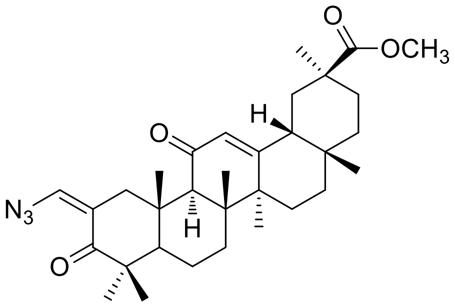

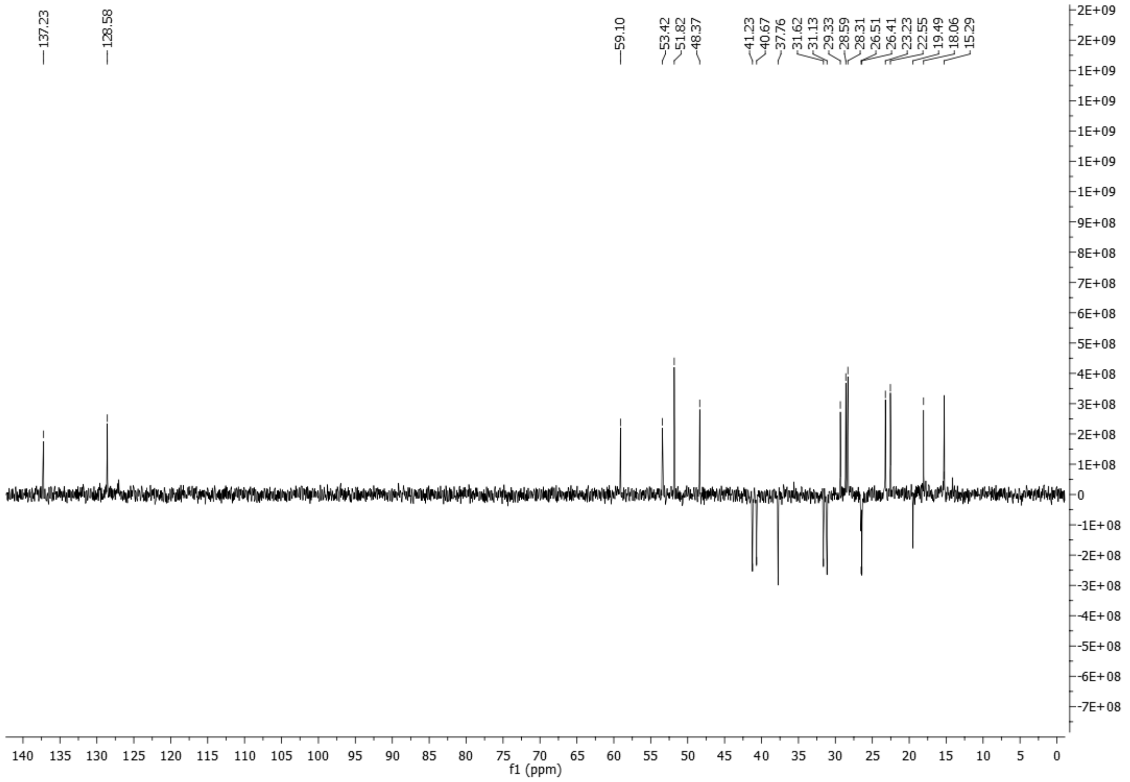

9. NMR data for compound 20

9.1. ^{1}H NMR spectrum for compound 20 recorded in $CDCl_3$

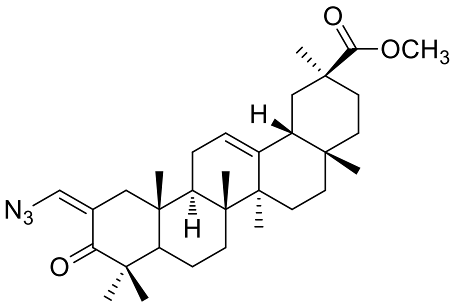

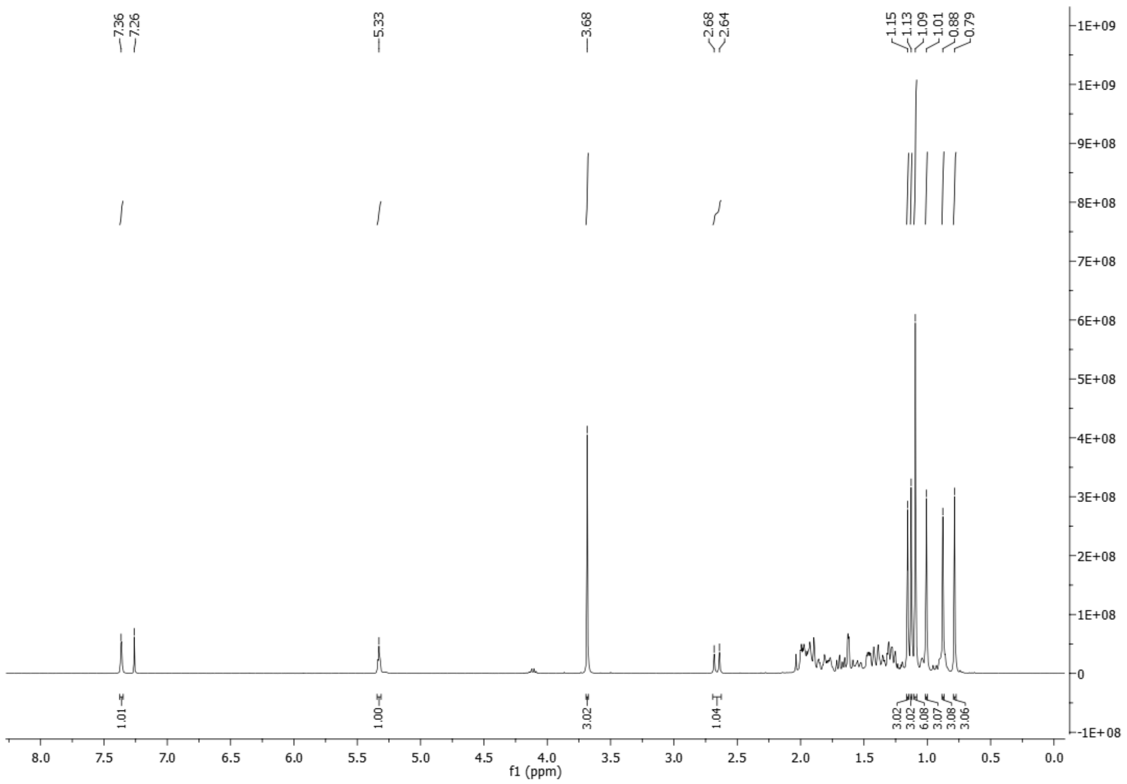

9.2. ^{13}C NMR spectrum for compound 20 recorded in $CDCl_3$

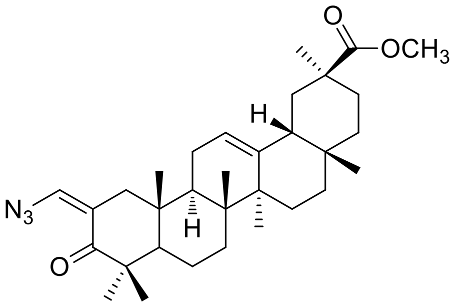

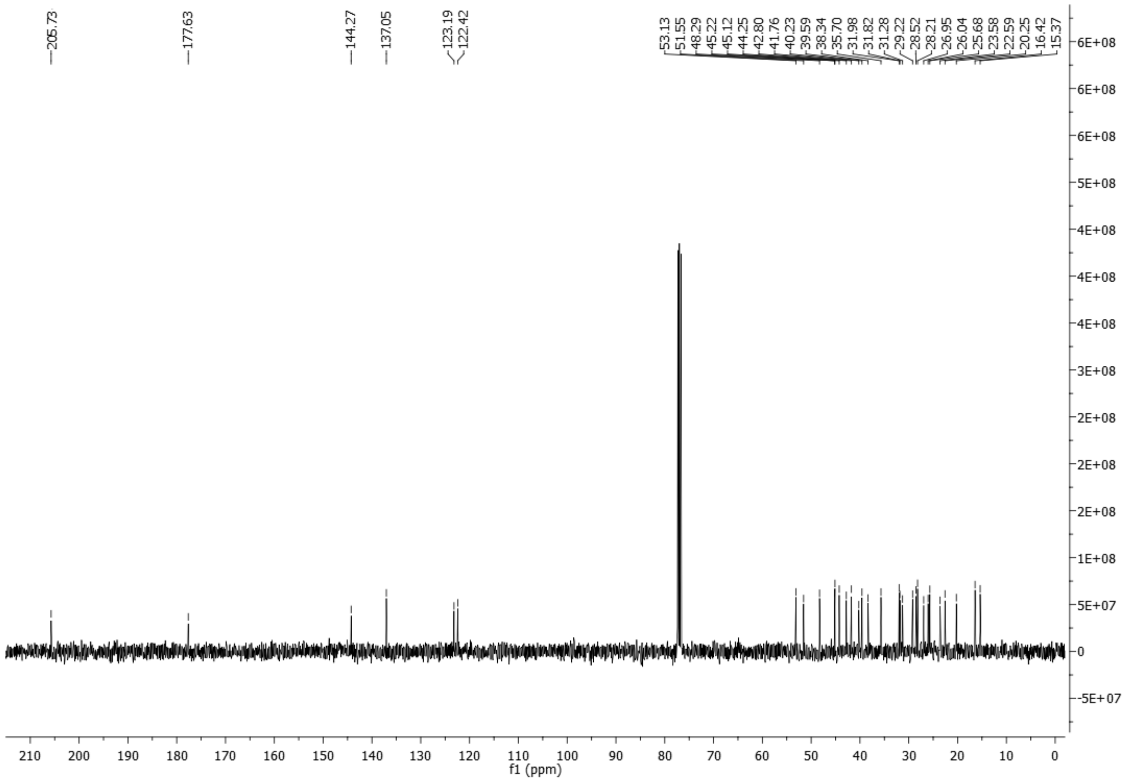

9.3. DEPT-135 spectrum for compound 20 recorded in $CDCl_3$

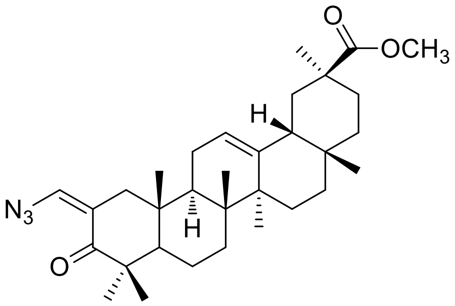

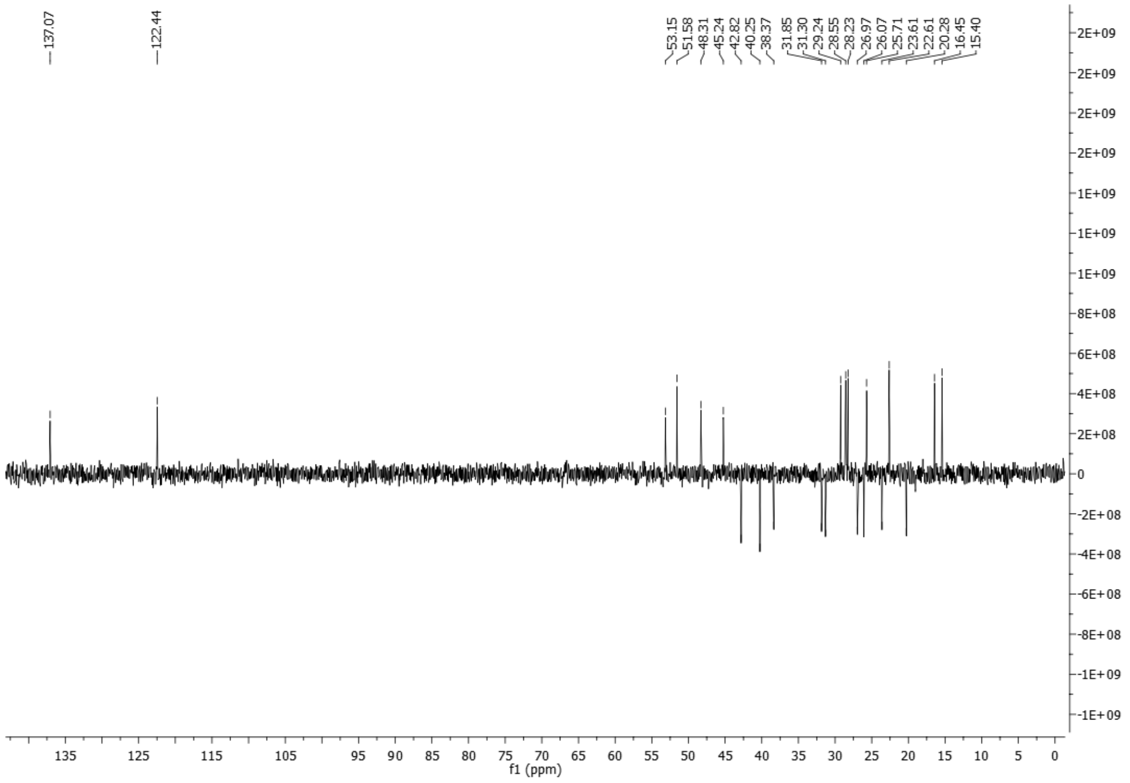

10. NMR data for compound 21

10.1. ^{1}H NMR spectrum for compound 21 recorded in $CDCl_3$

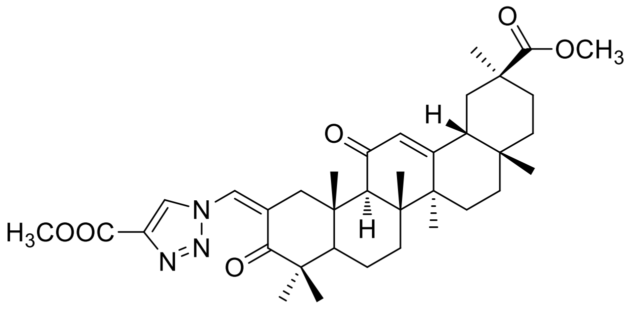

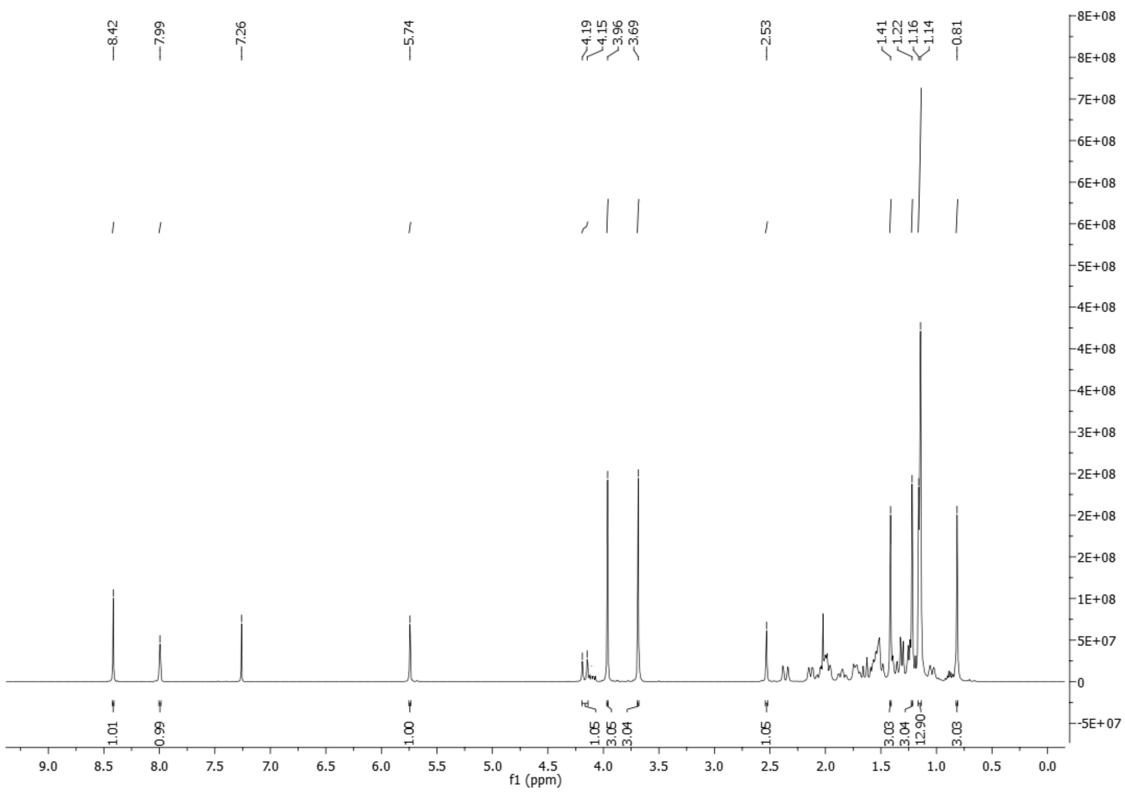

10.2. ^{13}C NMR spectrum for compound 21 recorded in $CDCl_3$

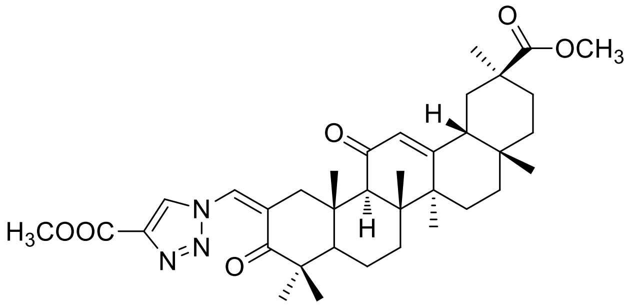

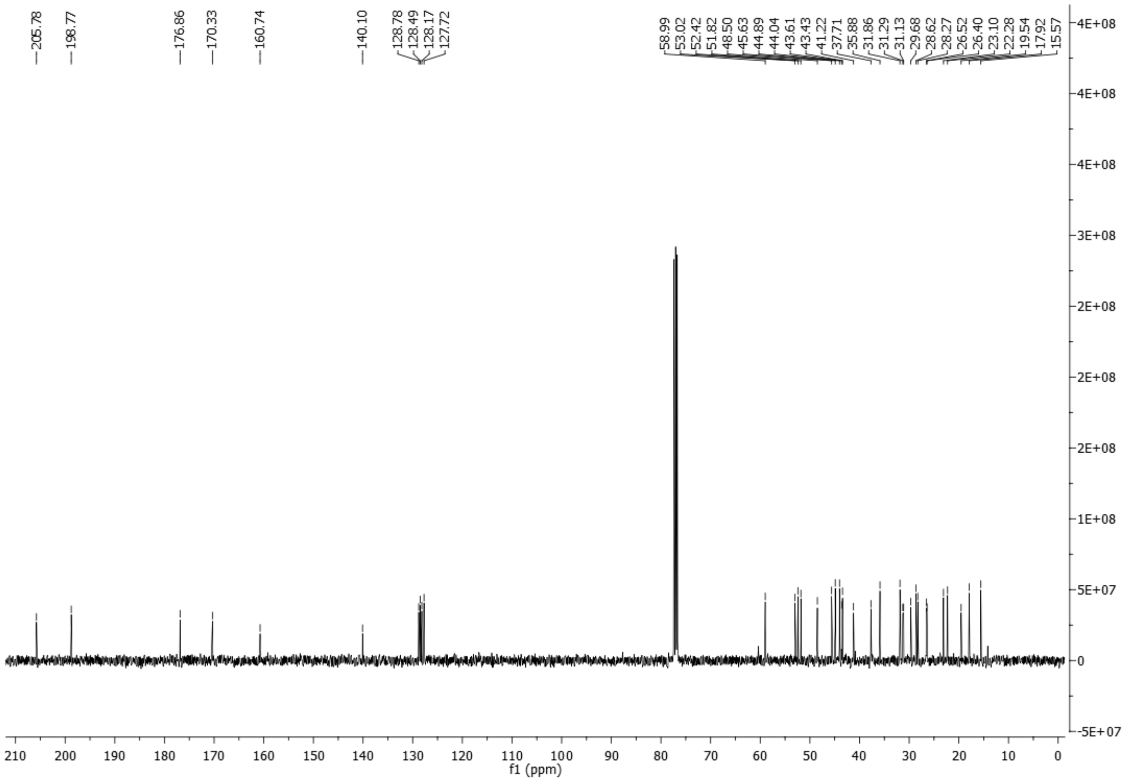

10.3. DEPT-135 spectrum for compound 21 recorded in $CDCl_3$

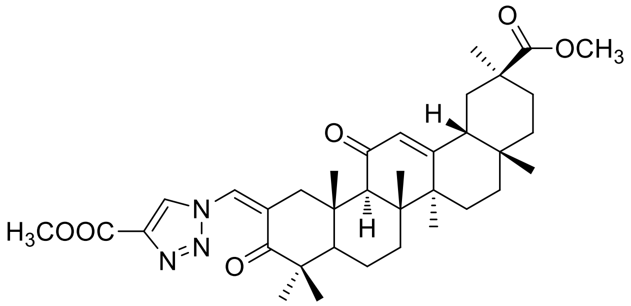

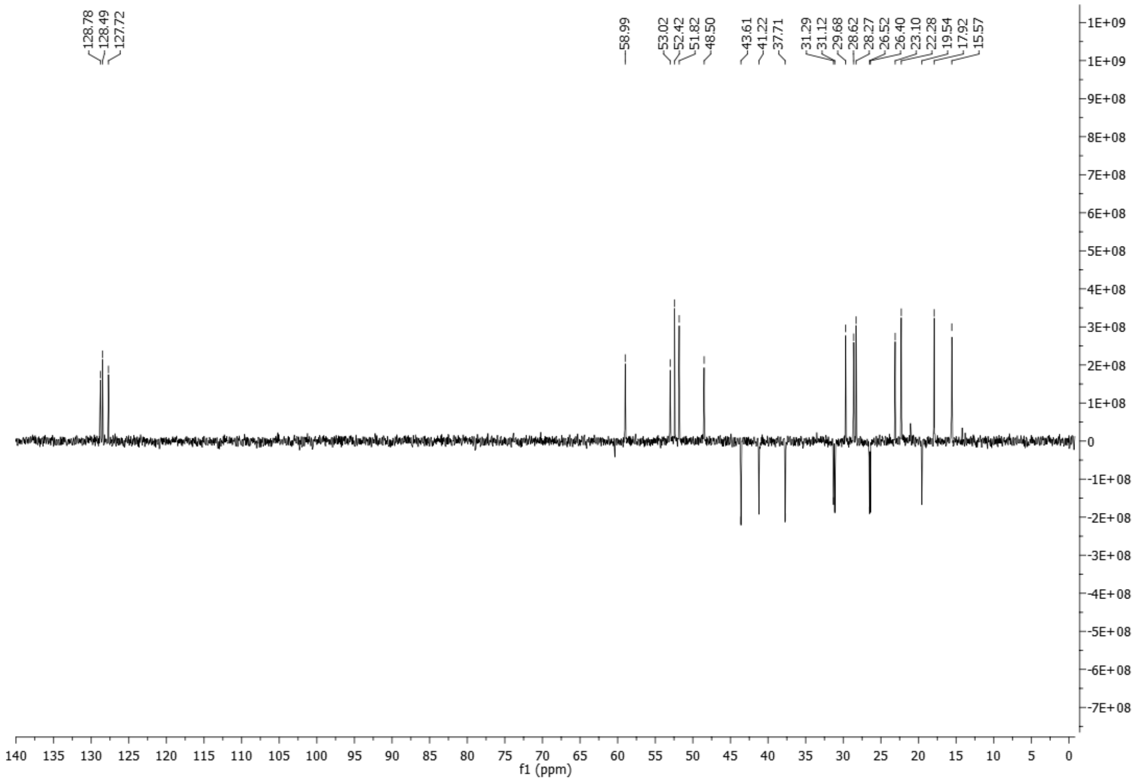

11. NMR data for compound 22

11.1. ^{1}H NMR spectrum for compound 22 recorded in $CDCl_3$

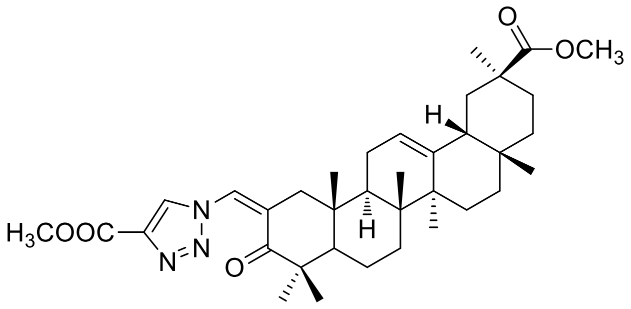

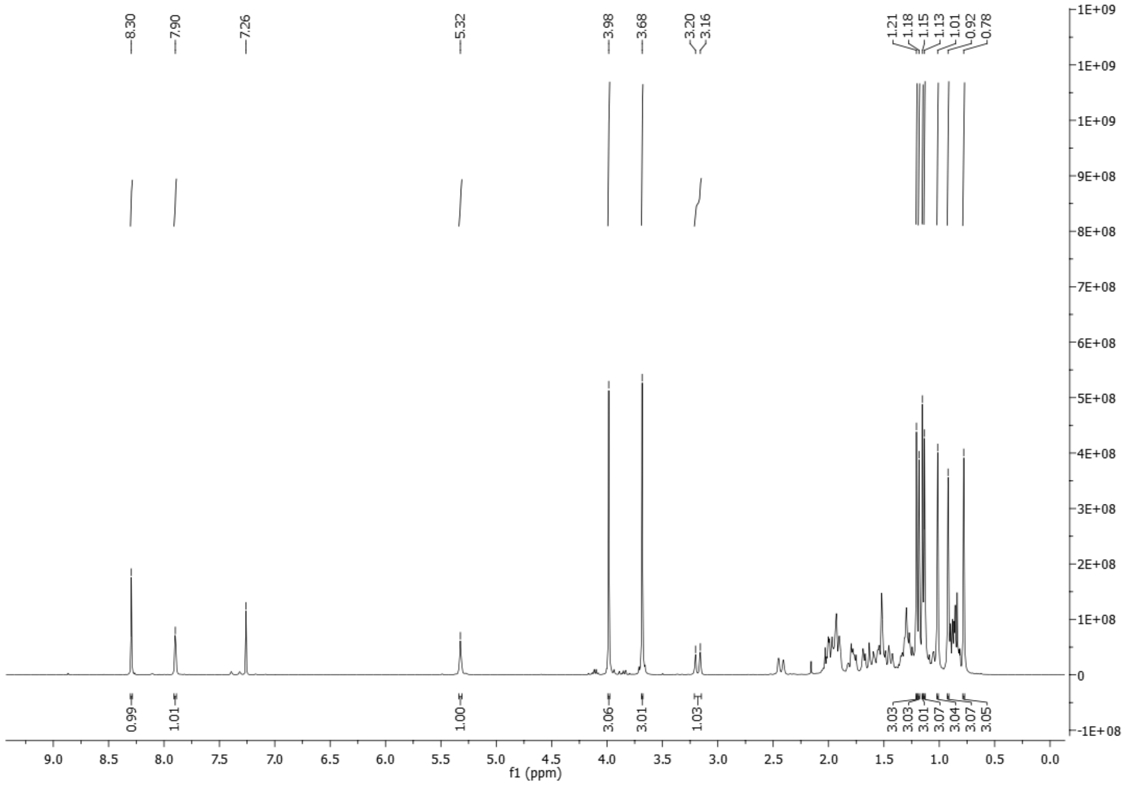

11.2. ^{13}C NMR spectrum for compound 22 recorded in $CDCl_3$

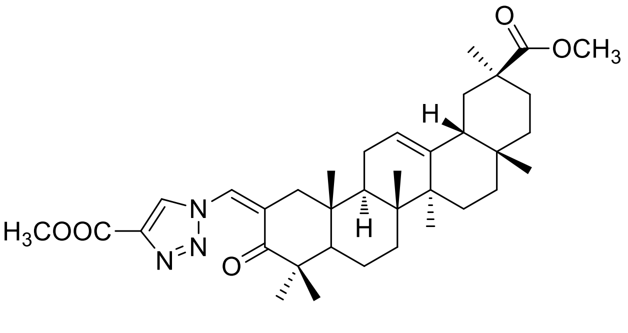

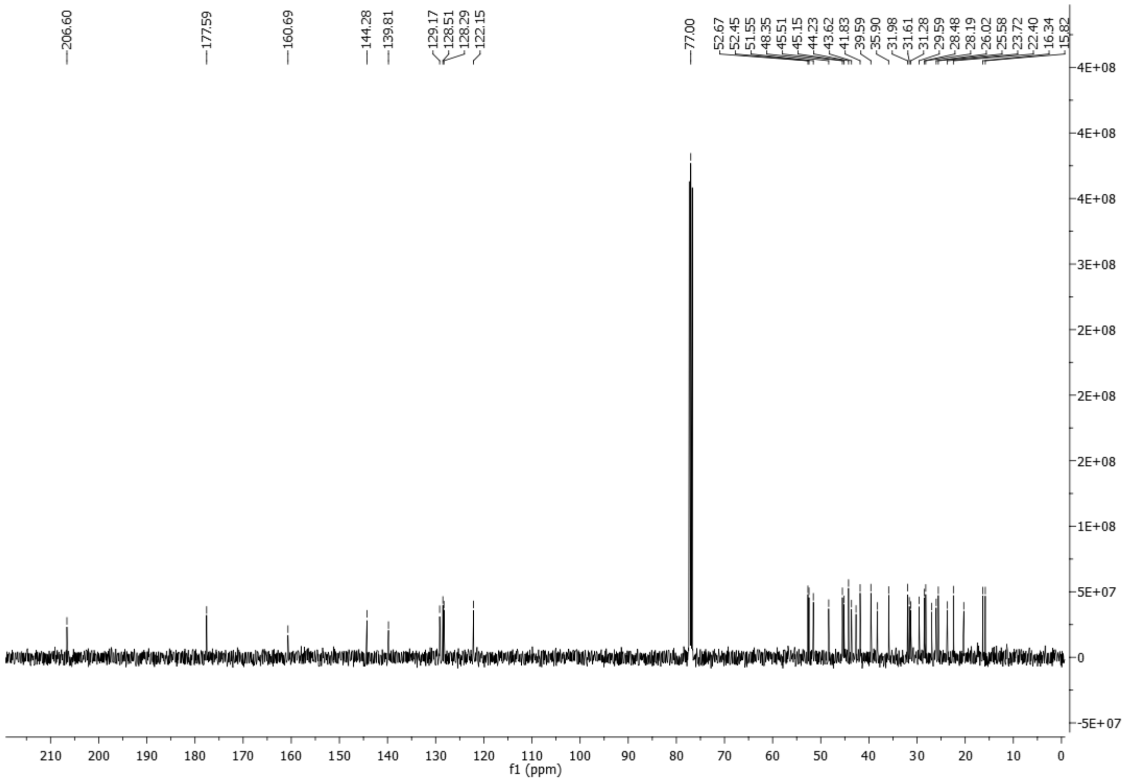

11.3. DEPT-135 spectrum for compound 22 recorded in $CDCl_3$

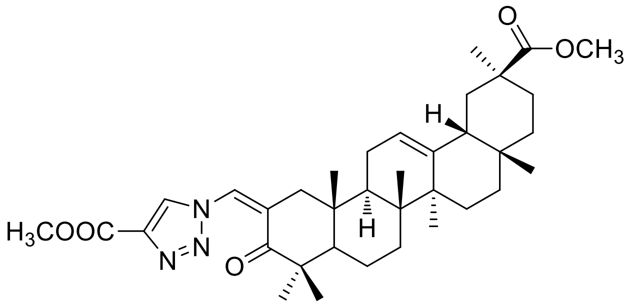

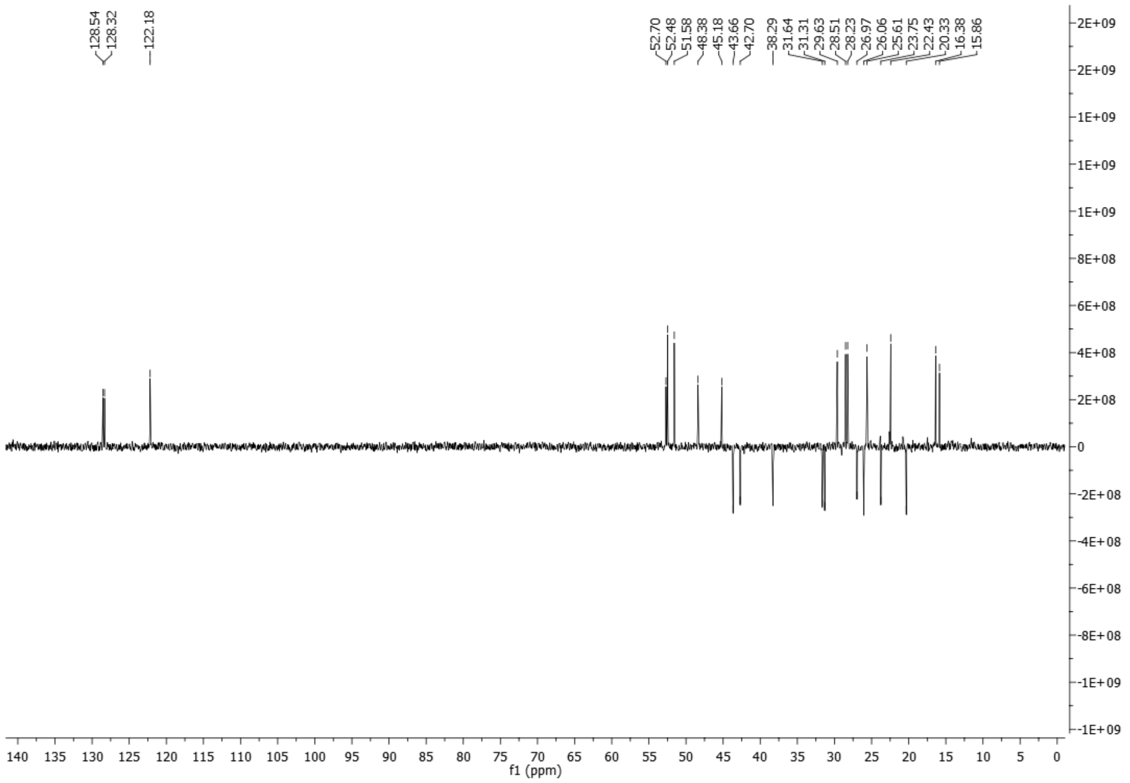

11.4. HMQC NMR spectrum for compound 22 recorded in $CDCl_3$

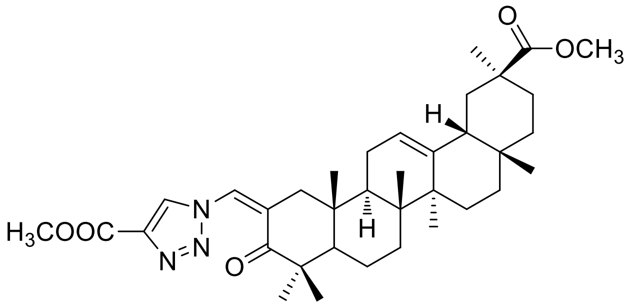

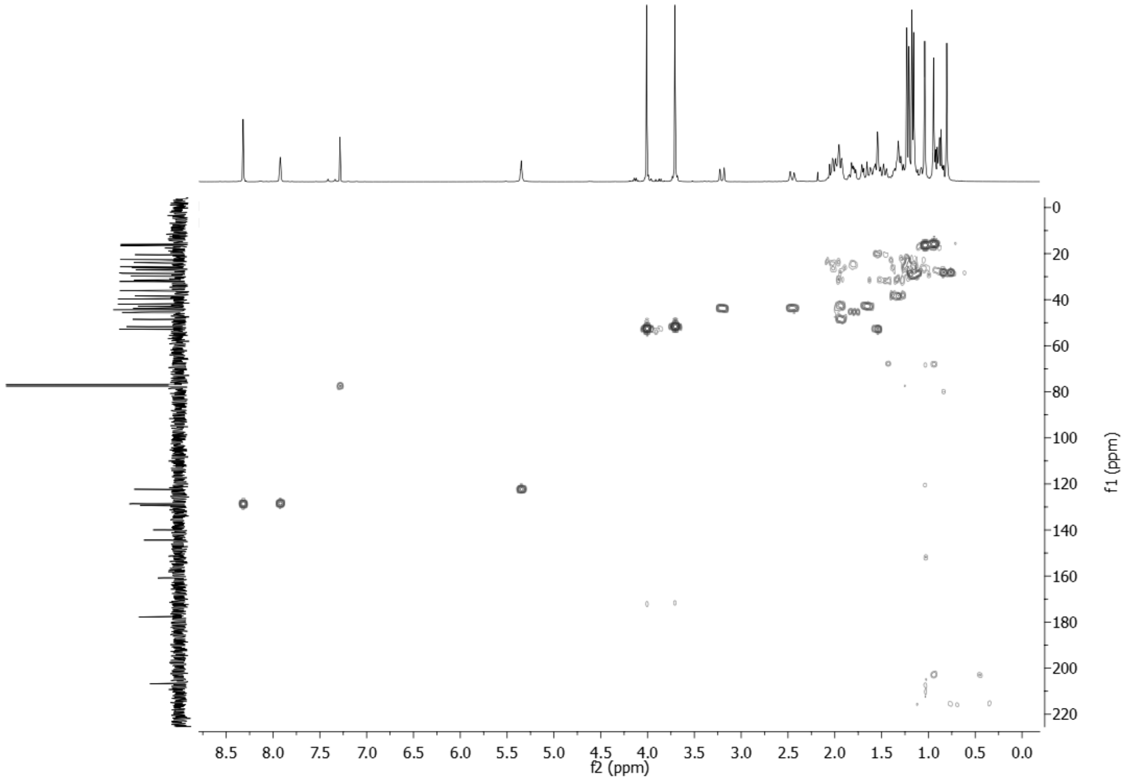

11.5. HMBC NMR spectrum for compound 22 recorded in $CDCl_3$

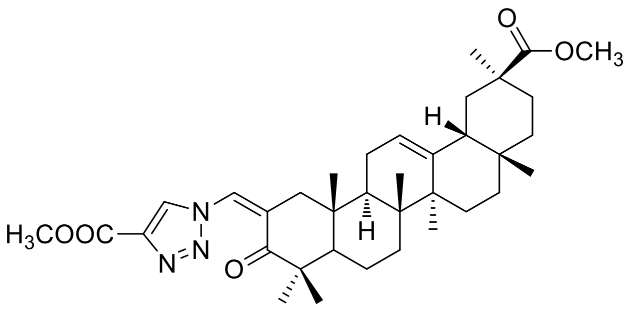

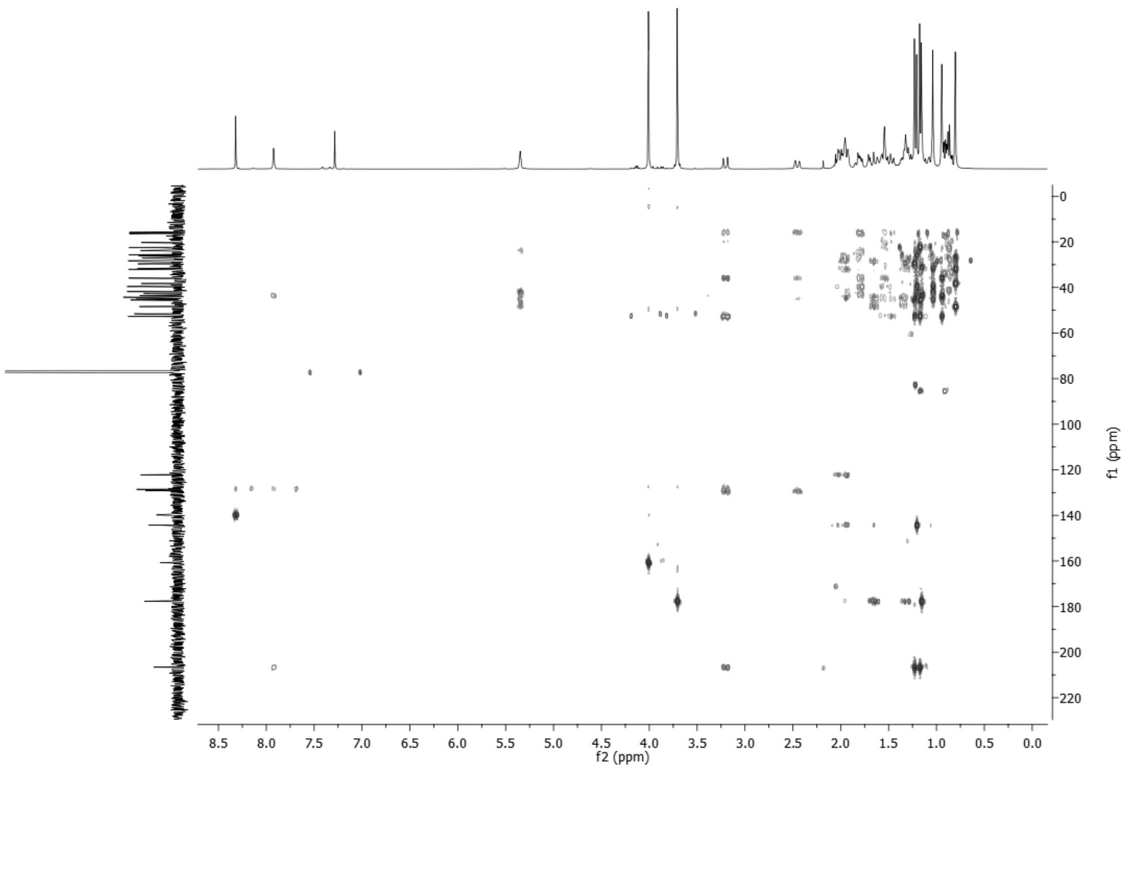

Supplement: Supplementary file 1 [file molecules-24-00766-s001.pdf]
